# *“Cute Companion”* or *“Awful Thing”*: Understanding Individual Differences in Attitudes to Social Robots as Pets

**DOI:** 10.1007/s12369-026-01453-4

**Published:** 2026-11-02

**Authors:** Shaun Macdonald, Cristina Fiani, Salma ElSayed, Mark McGill

**Affiliations:** 1 Glasgow Interactive Systems Section, University of Glasgow, University Avenue, Glasgow, Scotland G128QQ UK; 2 Design and Informatics, Abertay University, Bell Street, Dundee, Scotland DD11HG UK

**Keywords:** Individual differences, Social robots, Robot pets, Survey

## Abstract

For users unable to keep pets due to logistical, financial, or health reasons, social robots could offer a substitute. However, these robots remain uncommon in domestic use, and it is unclear how individual user traits or robot attributes impact receptivity or perceptions toward social robots as pets. We conducted a mixed-methods survey exploration (*N* = 163) of respondents’ initial receptivity and preconceptions regarding five robots with varied morphologies to understand whether these perceptions correlated with individual traits. We found that indi-vidual differences in anthropomorphism propensity, pet attachment and lower age all predicted positive initial perceptions of the social robots as domestic com-panions, guiding future expectations. Furthermore, a divide emerged between participants with non-negotiable philosophical objections to robot pets and those with addressable concerns around robot fidelity and agency. We discuss the impli-cations of user perceptions on the current feasibility of domestic robot pets and provide actionable directions for robot development.

**Supplementary Information:**

The online version contains supplementary material available at https://doi.org/10.1007/s12369-026-01453-4.

## Introduction

Keeping pets is a highly prevalent historical and contemporary activity. It has been shown to have many proven benefits, including facilitating social interaction, tackling loneliness, clinically improving mental health and physical health outcomes, and more [1–4]. Unfortunately, there are a multitude of reasons why people may be unable to keep pets, including tenancy restrictions [5], logistical and financial barriers [6], or health and accessibility concerns [7]. Social robots, which interact with humans by following behaviours and rules based on pre-defined social roles, could act as acces-sible, low-maintenance, allergen-free alternative to pets. Robot pets have shown the potential to emulate the benefits of pet interaction, however, they have primarily been applied in care and health [8–10]. Prior work has demonstrated that, within care home and medical settings and with receptive elderly or child users, socially assistive robots such as Nao and Paro can be psychologically and physiologically calming, evoke pleas-ant memories and act as social catalysts [8, 11–13]. The presence of social robots has also been found to be soothing or comforting in laboratory settings [14].

Despite their potential benefits, robot pets are still far from achieving widespread adoption in everyday homes. Even prominent models, such as Sony’s dog robot AIBO, sell at most tens of thousands of copies in Japan [15], a culture which historically has positive perceptions of social robots [16, 17]. Research highlights the inability of these robots to maintain utility or user interest, instead facing a *‘3-month barrier’* [16] to use, being seen as *‘briefly entertaining, but pointless’* [18], and user ratio-nale for adoption being modelled as a result of utilitarian concerns and hedonistic attitudes [19]. It is currently unclear, however, what proportion of people are likely to be receptive towards robots as domestic pets, or how users’ initial perceptions or preconceptions about these robots vary by individual differences in personality traits and demographics. Additionally, domestic robots may embody different morpholo-gies (e.g., animal-like, human-like or machine-like), changing users’ perceptions and expectations of them. While research has explored examples of how robot form fac-tors impact user perceptions, such as in regards to the uncanny valley effect between zoomorphic robots [20], or perceived social presence between different mechanomor-phic robots [21], we lack a clear understanding of how user early receptivity to robot pets varies between different morphologies, and how this may interact with individual differences. Finally, past work has not well explored how prior experiences with real pets may impact expectations and perceptions of robot pets as substitutes, which may also correlate with receptivity. As a result, robot designers and researchers face a gap in knowledge regarding expected user receptivity and perceptions for domestic robot pets, and how this varies across individual differences and different robots.

To address this gap, we present findings from an exploratory study survey of 163 participants from Europe, America and Southeast Asia, constituting a holistic mixed-methods exploration of how users perceive five social robots as potential domestic companions, based on audiovisual impressions. We explore how individual differences, including core demographics, Big Five personality traits [22], propensity to anthro-pomorphism [23], as well as pet attachment and ownership [24], impacted initial perceptions of these social robots. We further investigated how these individual dif-ferences interacted with perceptions of the five robots which comprised a spectrum of morphologies and capabilities, from zoomorphic (animal-like) robots aiming to evoke pet interaction, through anthropomorphic (human-like) and mechanomorphic (machine-like) designs, to an explicitly utility-focused vacuum robot. Respondents rated each robot’s animacy, likeability and intelligence, as well as their receptivity to each robot. We also captured rich qualitative data detailing the rationale for robot perceptions, as well as how these perceptions relate to existing pet experiences, and perspectives on the general concept of robots as pet alternatives. This mixed-methods survey provides researchers and designers with fresh insight into users’ early percep-tions of different social robots as domestic robots, which users are more likely to be receptive, the key desires and concerns they have for these robots, and how this varies across robot morphologies.

We found that younger participants with a greater propensity for anthropomor-phism were more likely to be receptive to social robots as potential pets. These participants, as well as those with higher pet attachment scores, also viewed robots as more likeable, animate and intelligent. However, we found no significant correlations between robot perceptions and the Big Five personality traits or gender. Our thematic analysis revealed an intriguing near-even philosophical divide between respondents. While 46% of respondents held non-negotiable objections to robot pets due to their artificiality, others held reservations that could be addressed by enriching a robot’s social responsiveness, evolving relationships and apparent agency. Users who were already receptive to robots as pet alternatives were motivated by visual appeal, prac-tical accessibility, and the evocative pet-like interactions. We discuss the implications of these findings on the future of domestic robot pet design, then present a series of actionable recommendations that support designers and researchers in identifying potentially receptive users of robot pets and improvement vectors to addressing the expressed needs of potential adopters.

We contribute the following:

1. New understanding of how user demographics, individual traits and pet attachment impact perceptions of five robots with varying morphologies and receptivity to them as domestic pets or companions;
2. Insight into users’ rationale for robot perceptions, divided into philosophical objections and addressable concerns;
3. Discussion and guidance for how designers could make robots more appealing to potential users as domestic pet alternatives.

## Background

### Utility and Hedonism in Robot Perceptions

When designing or deploying a social robot for domestic settings, users cannot be assumed to be receptive or accepting. It is well understood that some users feel a degree of fear or apprehension towards robots, with an estimated 20% of the population experiencing ‘robophobia’, often driven by lack of exposure [25]. Receptivity to robots also varies across cultures, e.g., agreement to use robots in Denmark (95%) has been measured as far higher than in Greece (57%) [25]. Individual differences in negative attitudes towards robots can also predict lower engagement and trust, particularly when the robot is framed as a social entity [26, 27].

When considering receptiveness towards robots in specifically domestic settings, major contributions have been made by de Graaf et al. [19, 28]. They model user acceptance of robots into *utilitarian* factors related to practicality and usability, and *hedonic* attitudes relating to concepts including companionship, sociability and enjoy-ment. Usefulness, or utility, is identified as the key factor to early acceptance and use intention, with long-term sociability, enjoyment and privacy concerns as additional important factors post-adoption. Furthermore, they also linked user expectations of the lifelikeness of the zoomorphic robot Pleo to greater feelings of intimacy and attrac-tion [28]. A survey of participant receptivity to three mechanomorphic social robots with digital assistant capabilities found that they were considered mostly from util-itarian perspectives, with a lack of long-term use cases and privacy risks as major concerns [18]. Meanwhile, Randall et al. approached the issue from a qualitative con-sumer research perspective, finding that early adopters of social household robots were often younger and technology-oriented, while mainstream adopters were price-sensitive, pragmatic and preferred zoomorphic forms [29]. Respondents across these works were primarily motivated by utility in the form of improving their health, productivity or mindfulness.

In their 2011 framework paper, Young et al. described Human Robot Interaction (HRI) as unique in evoking social and emotional responses, even during pure utili-tarian interactions, driven by the nature of robots as active players that occupy and traverse physical and social space [30]. Later work demonstrated how these hedonistic factors can emerge from interaction with utility-focused robots. For example, Grimme et al. explored how a vacuum robot adopted for practical purposes could later be mod-ified with a zoomorphic exterior that conferred pet-like social affordances, eliciting positive responses and pet-like interactions from care home residents [31]. Similarly, Sung et al. documented Roomba users ascribing robots with personality traits or modifying them with zoomorphic covers, resulting in more engagement with social interactions and assistive behaviours [32]. Finally, there has been some work exploring how personality traits shape robot perceptions, with Craenen et al. demonstrating the similarity-attraction effect can apply to social robots [33]. In this video study, 30 users observed the anthropomorphic robot Pepper signalling different personality traits by performing different gestures, tending to give higher Godspeed scale ratings when the robot’s apparent personality traits matched their own.

These works provide a useful framework for understanding how users rationalise their perceptions of robots. What remains unclear, however, is how individual dif-ferences, such as individual propensity for anthropomorphism and personality traits, affect receptivity to robots with different morphologies as domestic companions or pet substitutes.

### Influence of Different Robot Morphologies

Different morphologies, or physical embodiments, influence the interaction between users and robots by offering different social affordances. For example, users may judge that a robot is capable of conversational interaction if it has a human-like morphology, while robots with a soft exterior and animal-like features may invite pet-like affective touch or expectations of non-verbal interaction [34, 35]. If robots then fail to meet these expectations, distrust, misuse or lower acceptance can result [36].

Our approach took direct inspiration from prior works which similarly contrasted initial user perceptions of different robot morphologies. Pu¨tten and Kr¨amer used an online image survey study to cluster 40 robots with primarily anthropomorphic or mechanomorphic forms based on user’s perceived likeability, finding that humanoid robots rated higher [37]. Sasser et al. investigated changes in perceived social pres-ence between three morphologies: the humanoid Ubtech Lynx, mechanomorphic Anki Vector, and the Amazon Echo smart speaker [21]. They observed that each mor-phology offered social affordances that set user expectations and that Vector elicited higher perceived social presence than the humanoid robot, in large part due to its expressive and independent movement, which could be interpreted as a social cue. On the other hand, Bradwell et al. reported on a series of studies that contrasted perceptions of robots deployed in healthcare and social settings with five different morphologies: humanoid, mechanoid, realistic zoomorphic, abstract zoomorphic, and purely functional [38]. Generally, they found that zoomorphic designs were preferred in this context, as users had lower expectations of their capabilities, and they could evoke pleasant affective pet experiences. Meanwhile, mechanomorphic designs were viewed as less social and more functional, inhibiting their ability to elicit emotional responses or provide soothing care. Finally, humanoid robots had a strong social pres-ence but were viewed with distrust and anxiety, which the authors hypothesised to be due to the ‘uncanny valley’ effect.

The uncanny valley refers to a hypothesised effect in which user perceptions of robots with humanoid morphology shift from empathy to revulsion as they near but do not achieve lifelike realism [39]. Work has sought to understand the cause of this effect, such as Saygin et al., who used brain imaging to monitor responses to incon-gruent android movement [40], or to intervene with measures such as explicit external dehumanisation [41]. L¨offler et al. observed a similar uncanny valley effect within zoomorphic robots, suggesting that designers should avoid mixing realistic features (e.g., fur, snout) and unrealistic features (e.g., plastic, visible joints). These prior stud-ies demonstrate the differences in user perceptions that can emerge between robot morphologies. This motivated our investigation, expanding upon the scope of these works to explore how morphology impacts user receptivity and perceptions towards robots as domestic companions or pets, how this interacts with individual differences and documented qualitative rationale.

### Robots as Pets and Anthropomorphism

A key application area for domestic social robots is their potential to act as a substitute for pets. While historically pets served more utilitarian roles, they now serve emotional and hedonic needs [1]. Even if an individual would like to keep a pet, they may be unable to do so due to logistical, financial, hygiene or ecological concerns [6, 7]. This has been a key motivation for the deployment of zoomorphic robots in care settings, where they can emulate animal-assisted therapy, which has many proven benefits [4, 42, 43]. For example, Kramer et al. observed that the AIBO robot dog could emulate the social stimulation of hospital animal visits [44]. Similarly, pet-like robots such as Paro and the Joy For All companion cat have facilitated interpersonal social interaction and pet-like interactions in care home settings [8, 9, 45, 46] and in laboratory studies [14]. Katsuno et al. detail the integration of ‘haptic creatures’ in users’ lives, highlighting instances in which they can *anthropomorphise* these robots as they elicit deep-seated memories of past pet interaction, but also a ‘three-month barrier’ to use driven by a lack of long-term engagement [16]. Anthropomorphism is the attribution of human characteristics such as personhood, emotions or intentions [47], and the propensity for it varies between people [23]. From a relational perspective, this can allow individu-als to fulfil emotional and social needs and ascribe personality to ‘companion objects’ [48], which social and zoomorphic robots explicitly provoke with evocative form fac-tors, interactions and apparent autonomous agency [47]. This was demonstrated by Osawa et al., who observed that the addition of expressive robot faces drove the anthropomorphism of everyday objects [49]. Research also explored how anthropo-morphism related to the use of virtual pets, indicating that it can be facilitated by tailoring the pet’s apparent personality to match the user’s [50]. Lin et al. identified three categories of virtual pet users: *Pet-keepers* who prioritise believable social pet-like interaction, *Animal Teammates* who value partnership and companionship with pets in shared tasks, and *Cool Hunters* driven by collecting virtual animals [51]. What remains unknown, however, is how individual differences such as individual propensity for anthropomorphism has personality traits has on receptivity to robots as domestic pets or companions.

Overall, past research has identified factors that may influence how users perceive social robots. These factors demonstrate variability in perceptions based on percep-tions of the robot or its use. However, it is still unclear how many users are receptive to different social robots as domestic companions or pet substitutes, how their rationale and perceptions vary based on individual differences, and how this interacts with mor-phology. By addressing this gap, we enable designers and researchers to have informed expectations of how receptive users are likely to be to the robot they are developing and deploying and the key factors the field must address for this use case.

## Survey Design & Methodology

We conducted a video survey study to explore how receptive users are to five varied robots as domestic companions, extending prior approaches in three key ways. First, we investigated whether individual differences in Big Five personality traits, pet owner-ship and attachment, propensity to anthropomorphism and attitudes towards robots, correlated with initial attitudes toward domestic social robots. Second, we observed how these individual differences interact with the perception of potential domestic robots with different morphologies and purposes. Finally, we explore participant per-ceptions as well as their rationale using a mixed-methods approach. The survey was approved by the institution’s Ethics Committee, and built using the online platform Qualtrics^1^. Data collection and storage were aligned with the GDPR guidelines. The complete survey can be seen in the Supplemental Materials. The survey procedure and data gathered were as follows:

**Step 1 - Information, Consent, Demographics & Individual Differences**: Participants aged 18 or older were welcomed to the survey, informed of the survey contents and aims, and then asked to sign a consent form. Participants reported their age, gender, education level and employment status. The survey then administered two scales deployed to investigate potential individual differences in personality traits in robot receptivity: the Big Five Inventory 10-Item Scale (BFI-10) [22] and the Indi-vidual Differences in Anthropomorphism Questionnaire (IDAQ) [23]. The BFI-10 is a short survey used to measure the Big Five personality traits: extraversion, agreeable-ness, conscientiousness, neuroticism, and openness. We used the BFI-10 to understand if these traits predict receptivity for robot pets, as past work has connected person-ality traits to technology acceptance [52], while attempting to maintain a relatively low survey completion time per participant and reduce unfinished entries, following pilot testing. We also applied the IDAQ, which measures an individual’s propen-sity to anthropomorphise animals, technology and natural phenomena, to understand whether this trait correlated with robot perceptions or receptivity. Finally, we contex-tualised our findings by measuring participants’ preconceptions toward robots using the Negative Attitude Towards Robots Scale (NARS) [53].

**Step 2 - Experience with Pets**: Participants were then asked about their experience and relationships with pets. First, they were asked if they currently live with one or more pets, or had done so in the past. If they answered no, all the following pet-related questions were skipped. If they answered yes, they were first asked what type of animal the pet was. They were then administered the 26-item Pet Attachment Questionnaire (PAQ) [24], designed to assess attachment anxiety and avoidance in pet relationships, to understand if these factors may influence judgement of robots taking on pet-like roles. Finally, participants filled out two mandatory free-text questions, which asked them to (1) summarise the relationship they had with their pet, highlighting experiences they felt were important or beneficial, and (2) report how emotionally close they felt to this pet, highlighting intimate experiences or interactions.

**Fig. 1:**
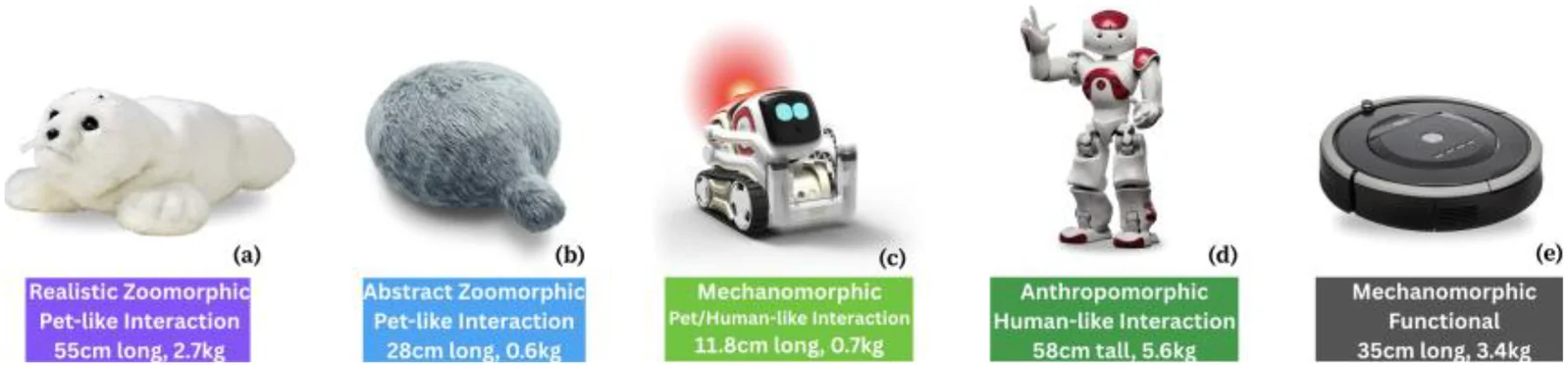
Five different robots across an established range of morphologies [38] were presented to participants: **a**) Paro, **b**) Petit Qoobo, **c**) Cozmo, **d**) NAO, **e**) Roomba

**Step 3 - Receptivity to Different Robots as Domestic Companions or Pet Substitutes**: We sought to understand how users’ initial perceptions of robots and receptivity to robots as domestic companions or pet substitutes varied between individuals and between robots. As in prior works [20, 21, 38], we pre-sented participants with a range of five robots varied by morphology (zoomorphic, anthropomorphic, mechanomorphic) and functionality (pet-like interaction, human-like interaction, purely functional). These robots were: Paro^2^, Petit Qoobo^3^, Cozmo^4^, NAO^5^, Roomba^6^ (robot morphologies and functions are shown on Fig. 1). When selecting morphologies to present to participants, we drew directly upon the schema used by Bradwell et al. and aesthetic categories identified by Mobed et al. [38, 54] to select robots representing realistic zoomorphic, abstract zoomorphic, anthropomorphic and mechanomorphic morphologies and interaction. We specifically chose to present a contrasting pair of realistic (Paro) and abstract (Petit Qoobo) zoomorphic robots, as prior work has found that realistic zoomorphic robots could be more likeable [20] or preferable to older users [38]. Four of the robots we embody morphologies that offer social affordances for pet-like interaction (Paro, Petit Qoobo) or human-like interac-tion (NAO, Cozmo). We chose to contrast these robots against the Roomba for three reasons: (1) to act as a control as a purely functional robot without specific social features, (2) despite a lack of social features, research has shown users personifying these vacuum robots [32, 55, 56], and (3) vacuum robots are the only category that is currently prevalent in domestic contexts (estimated within 9.7% of US households in 2023^7^).

As this was a survey study participants were not able to directly interact with robots, we adopted an established video survey study approach to understand their initial impressions of each robot by presenting them with images and videos [57, 58]. Participants were shown the five robots in a random order. For each robot, partici-pants were shown an image of the robot and a one-minute video that displayed how each robot looked, moved and sounded, as well as core features and communication modalities. In order to observe how their perceptions of these robots varied, they filled out three standardised Godspeed scales to measure perceived animacy, likeabil-ity and intelligence of the robot [59] after they watched each video. It should be noted that robot attributes, particularly intelligence, are difficult to for participants accu-rately assess in a video study without in-person interaction. However, we aimed to understand how their initial perceptions of these attributes varied between robots and respondents. Finally, they rated how receptive they were to the robot as a potential household pet on a 7-point Likert scale, and reported their rationale for this rating in a mandatory free-text field, to help us understand what drives any differences in receptivity.

**Step 4 - Pet-like Interaction and Future Advancements**: Once all five robots had been rated, participants reported if they thought there were positive pet-like experiences they could share with a robot on a 5-point Likert scale. If so, they were asked what kind of robot and experiences they thought of, in a mandatory free-text answer. Finally, they were asked two further free-text questions: (1) what they perceived the key differences were between real pets and robots as domestic pets or companions, and (2) if they could think of any future technological advancements to make robots more suitable as household companions. At the end of the survey, which took *≈* 20 min, participants were entered into a prize draw for a £50 shopping voucher.

### Recruitment & Participants

We utilised snowball sampling across a wide variety of channels to recruit participants in March 2024. Individuals aged 18 or over were invited to take part in the survey via advertising on (1) University channels in the UK, Thailand and Canada, (2) social media channels (Facebook, LinkedIn, BlueSky and X), (3) online discussion boards (subreddits including r/robotics and r/samplesize) and (4) physical posters on local community boards in [anonymised]. Automated survey responses were monitored via two attention check questions, and Qualtrics straightlining detection, which resulted in the exclusion of 15 responses from the dataset, leaving a total of 163 completions. 68 (42%) identified as female, 80 (49%) as male, 9 (5.5%) as non-binary and the remaining 6 (3.7%) preferred not to say. 108 (66%) of participants were below 35 years old, while 55 (34%) were 35 or older (see Table 1 for a full demographic breakdown. Based on the latitude and longitude of survey submissions, the majority of responses came from the UK (66%), followed by Thailand (20%), the USA (5.5%), Germany (3.7%), Sweden (1.8%), while remaining 3.1% came from five other countries. Our sample was educated, with 50 (31%) holding a Bachelors degree, 47 (29%) holding a graduate degree, 37 (23%) partway through university with no degree and 23 (14%) having finished secondary school. Finally, half of the participants were students (82, 50%), while 66 (40%) were employed, 6 (3.7%) were retired, 3 (1.8%) were unemployed and 6 (3.7%) reported ‘other’.

**Table 1:** Participant demographics data

Gender	*n* (%)	Age Bracket	*n* (%)	Location	*n* (%)	Education	*n* (%)	Employment	*n* (%)	Pets?	*n* (%)
Male	80 (49%)	18–24	63 (39%)	UK	108 (66%)	Graduate Degree	47 (29%)	Student	82 (50%)	Yes	137 (84%)
Female	68 (42%)	25–34	45 (28%)	Thailand	32 (20%)	Bachelors Degree	50 (31%)	Full Time	48 (29%)	No	26 (16%)
Non-Binary	9 (5.5%)	35–44	25 (15%)	USA	9 (5.5%)	In University	37 (23%)	Part Time	18 (11%)		
Not given	6 (3.7%)	45–54	10 (6.1%)	Germany	6 (3.7%)	Secondary School	23 (14%)	Retired	6 (3.7%)		
		55–64	12 (7.4%)	Sweden	3 (1.8%)			Unemployed	3 (1.8%)		
		65+	8 (4.9%)	Other	5 (3.1%)			Other	6 (3.7%)		

### Data Analysis

#### Quantitative Analysis

We conducted quantitative analysis of the subjective robot Likert scale ratings, includ-ing the participant as a random variable. Shapiro-Wilk tests confirmed that animacy, likeability, intelligence and receptivity data (*p <* 0.001) were not normally distributed.

Thus, we used the Aligned Ranked Transform (ART) [60] to allow us to conduct two-factor parametric tests. First, we investigated the impact of different robots presented to participants on their perception ratings. After this, we explored whether individual differences impacted these ratings, and if they had any significant interactions with the robot presented not explained by the main effect. We conducted this statistical analysis using R Studio Version 2025.05.1 + 513 and the *ART*, *emmeans* and *eff size* packages. Descriptive statistics, significance testing, *post hoc* contrasts and Cohen’s D effect sizes are discussed in Sect. 4.1.

#### Qualitative Analysis

To analyse free-text answers, we applied thematic analysis [61]. Two researchers conducted an initial inductive coding pass on an identical 25% data subset. Both researchers then met to synthesise their codes into an initial codebook^8^. One researcher used the agreed codebook to re-code the entire dataset, making alterations as required following discussion with the second researcher. Both researchers then conducted axial coding to group related codes into categories through collaborative discussion, which resulted in a final codebook of 152 codes, grouped in 7 categories and 22 sub-categories, which can be seen in the Supplimental Materials. Both researchers then used this codebook to construct and review four descriptive themes (see Sect. 4.4).

## Results

### Individual Difference Summary Statistics

#### Personality Traits (BFI-10) and Anthropomorphism (IDAQ)

When examining mean BFI-10 personality trait values (on a scale of 1–5) we found the distribution of personality traits of our survey respondents to be similar to that of Rammstedt’s original reference group [22]. Our sample exhibited slightly higher Openness (3.73, *sd* = 0.94)), Agreeableness (3.44, *sd* = 0.90) and Neuroticism (3.23, *sd* = 1.02), moderate Conscientiousness (3.26, *sd* = 0.94), and slightly low Extraver-sion (2.66, *sd* = 1.01), with similar standard deviations. Participants’ mean summative IDAQ score (range 0–150) was 50.53 (*sd* = 22.1), with scores ranging from 8 to 114.

#### Negative Attitude Towards Robots (NARS)

Our sample scored a mean score of 18.1 (*sd* = 3.61) on the first NARS subscale (max score = 30), which indicates slight negative attitudes toward *interaction with robots*. Their mean score on Subscale 2 (max = 25) was 13.6 (*sd* = 4.08), which indicated neu-tral attitudes towards *robots in social spaces*. Finally, our sample scored a mean 8.39 (*sd* = 2.75) on the third subscale (max = 15), suggesting slight concern regarding *emo-tional interaction with robots*. Overall, these scores suggest our sample held slightly negative attitudes toward robots when compared with prior work [53, 63, 64].

#### Pet Ownership and Attachment

84% of participants reported having some current or past experience living with, or having lived with, one or more pets. Of these 137 respondents with domestic pet experience, 56.2% reported living with a cat, 52.6% with a dog, 31.4% with fish, 23.4% with a rodent, 16.1% with a bird, 11.7% with a reptile and 11.7% with another unlisted species. Our sample scores 22.7 (*sd* = 13.4) on the Pet Attachment Question-naire avoidance scale, which has a score range of 13–91, suggesting relative comfort with emotional closeness to pets. Respondents’ mean PAQ anxiety score was 31.8 (*sd* = 16.4), indicating moderate anxiety about pet wellbeing and absence.

### Robot Perception and Receptivity Across All Participants

**Fig. 2:**
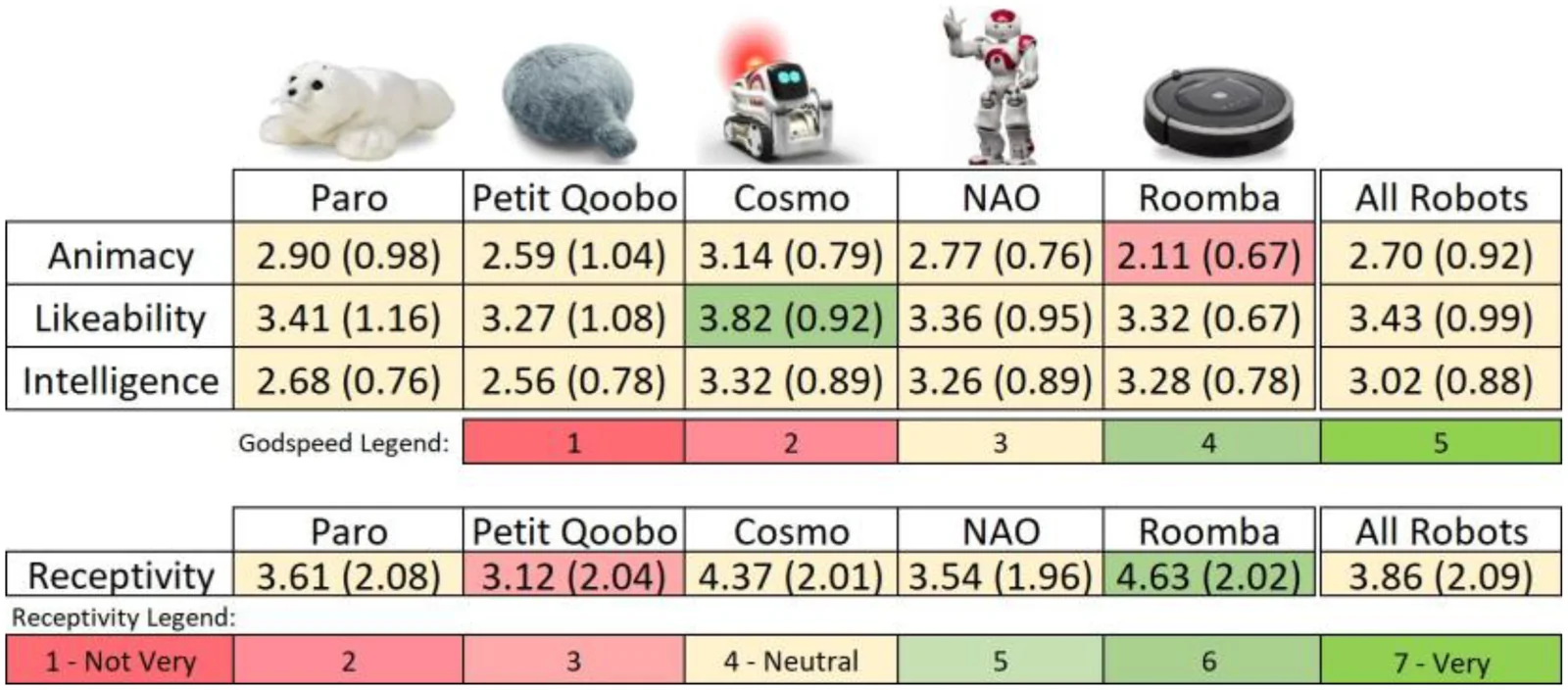
Participant ratings per robot for three Godspeed scales: animacy, likeability and intelligence; and for how receptive they would be to that robot as a household companion. Mean numerical Likert scale and standard deviation are shown per cell

Participants’ mean ratings (with standard deviations) for robot animacy, likeability and intelligence based on their audiovisual impressions of each robot, as well as their receptiveness to each robot as a pet alternative, are reported in Fig. 2. Participant ratings for perceived robot likeability, intelligence, animacy and receptivity, based on initial video impressions, were all positively correlated with strong coefficients (*r >* 0.5). The importance of robots displaying animacy was highlighted by the strong correlation between animacy and likeability (*r* = 0.71). The full correlation matrix between these ratings can be seen in Table 2.

At the end of the survey, participants were asked to rate whether they thought that there were positive pet-like experiences they could share with each robot on a 5-point Likert scale, based on their initial impressions. Responses were spread, with 14.1% responding ’Definitely Yes’, 36.8% responding ’Probably Yes’, 16.0% responding they were ‘Unsure’, 17.2% responding ‘Probably Not’, and the final 15.9% responding ’Definitely Not’. Single-factor ANOVAs found no significant impact of any individual differences on this rating (*p >* 0.5).

**Animacy**: We found that mean animacy ratings ranged from low to neutral ratings (2.11–3.14). A single-factor ANOVA found a significant effect of robot on animacy ratings (*F* (1, 4) = 64.3, *p <* 0.001). *Post hoc* pairwise t-testing with Tukey P-value adjustment (*α* = 0.05) blocked by participant was then conducted. This found that the Roomba had significantly lower animacy ratings (*m* = 2.11) than all other robots, with large effect sizes (0.8 *< d*, *p <* 0.001). Additionally, Cozmo had positive contrasts with Nao, Paro and Petit Qoobo, and Paro was rated higher than Petit Qoobo, all with medium effect sizes (0.3 *< d <* 0.8, *p <* 0.05).

**Fig. 3:**
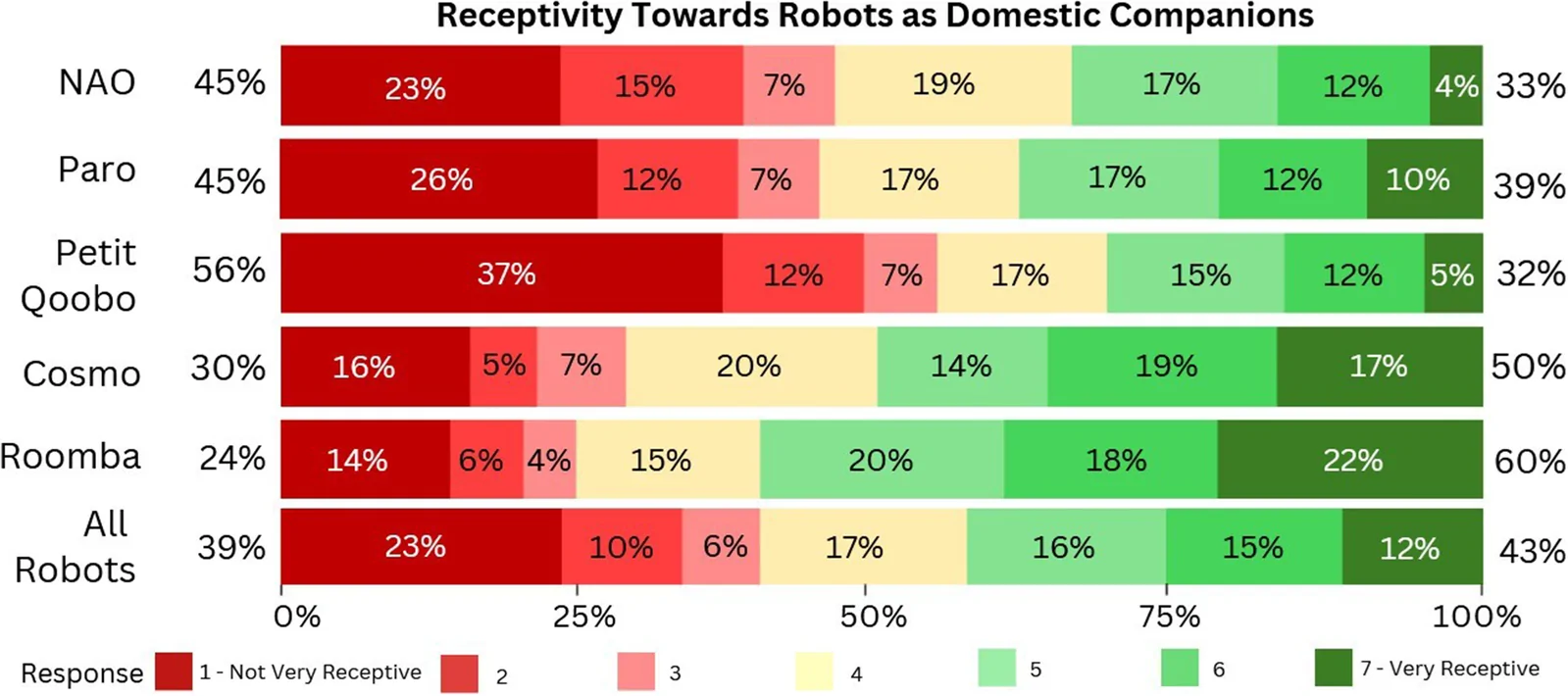
Proportion of Likert scale ratings for robot receptivity for each of the five robots presented

**Table 2:** Correlation matrix of Godspeed scale robot perceptions and robot receptivity, displaying r coefficients for correlations between each variable

Receptivity	Receptivity	Likeability	Animacy	Intelligence
Receptivity	-	0.65	0.51	0.51
Likeability	0.65	-	0.71	0.61
Animacy	0.51	0.71	-	0.50
Intelligence	0.51	0.61	0.50	-
Heatmap Legend:	-1 to -0.7	-0.7 to -0.3	-0.3 to 0	
	0 to 0.3	0.3 to 0.7	0.7 to 1	

**Likeability**: Four robots received neutral mean likeability ratings (3.27–3.41) that were close to the neutral scale value of 3, while Cozmo received higher ratings (3.82). A single factor ANOVA also found a significant difference between robots on likeability ratings (*F* (1, 4) = 18.0, *p <* 0.001). *Post hoc* contrasts revealed that Cozmo was rated as significantly more likeable (*m* = 3.82) than every other robot, with medium effect sizes (0.3 *< d <* 0.8, *p <* 0.05).

**Intelligence**: Mean intelligence ratings for all robots were in a low to middling range (2.56–3.28). The robot had a main effect on intelligence ratings (*F* (1, 4) = 73.2, *p <* 0.001). Pairwise testing indicated that participants considered all three non-zoomorphic robots, Cozmo (*m* = 3.32), Roomba (*m* = 3.28) and NAO (*m* = 3.26) to be significantly more intelligent than both zoomorphic robots, Paro (*m* = 2.68) and Petit Qoobo (*m* = 2.56). Additionally, all six of these contrasts had large effective sizes (0.8 *< d*, *p <* 0.001).

**Receptivity**: A breakdown of Likert scale responses (see Fig. 3) showed that par-ticipants were divided in their initial receptivity to robots, with 39% rating their receptivity across robots as low (1–3 out of 7), 43% reporting receptive answers (5–7) and 17% giving neutral answers (4). Participants tended to be more strongly neg-ative than positive, however, with 23% of participants were strongly non-receptive versus 12% strongly receptive. We found significant differences in receptivity between different robots (*F* (1, 4) = 23.7, *p <* 0.001). *Post hoc* contrasts revealed users were sig-nificantly more receptive to Cozmo and Roomba than the other three robots. Cozmo positively contrasted with Paro, while Roomba positively contrasted with Paro, Petit Qoobo and NAO, all with medium effect sizes (0.3 *< d <* 0.8, *p <* 0.05). Additionally, Cozmo positively contrasted in receptiveness with Petit Qoobo with a large effect size (0.8 *< d*, *p <* 0.001).

### Impact of Individual Differences on Robot Perception

#### Impact of Demographic Factors

We explored the impact upon initial robot perceptions of the following demographic factors: age, gender and pet ownership. For each of these demographics, we conducted four two-factor ANOVA to search for main effects, or interactions with different robots, on each robot rating. Only one demographic factor had a significant impact on robot ratings: age had a significant main effect on all four robot rating measures, as well as a significant interaction with the robot presented on animacy ratings (see Table 3).

**Fig. 4:**
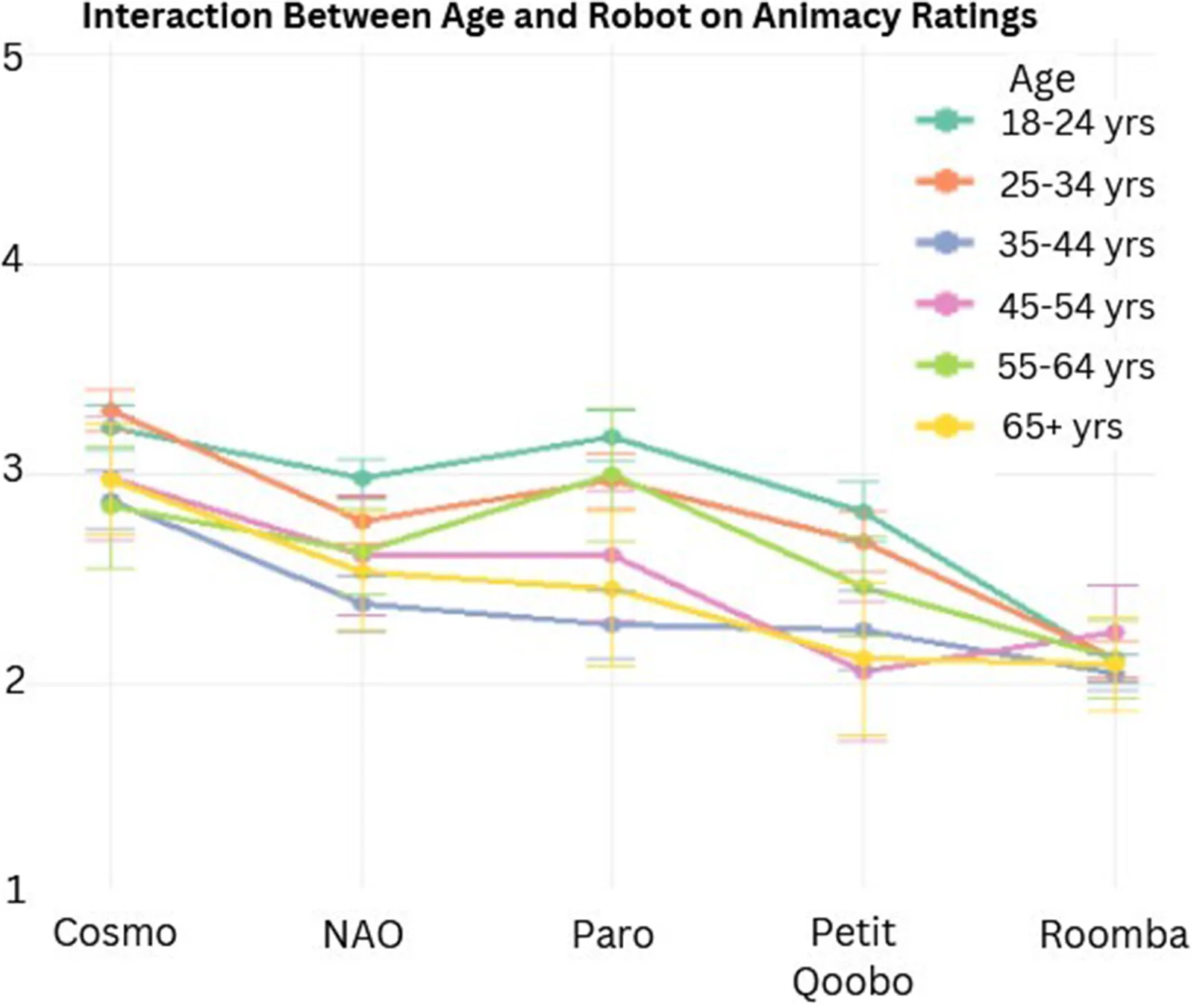
Interaction plot showing how animacy ratings varied depending on both the robot shown and age group. Younger age groups tended to give higher animacy ratings for all four social robots, but not for the Roomba

*Post hoc* analysis found significant contrasts between the 18–24 and 35–44 age groups in all four robot ratings (*p <* 0.05). The younger group gave robots higher ratings for likeability, animacy and intelligence, with large effect sizes (0.8 *< d*, *p <* 0.001), and were also more receptive, with a medium effect size (0.3 *< d <* 0.8, *p <* 0.05). These differences did not apply to animacy ratings of the Roomba, which all age groups rated similarly (see Fig. 4).

**Table 3:** Main effects and interactions of age and robot presented on animacy likeability, intelligence and receptivity ratings

Factor		Animacy Likeability Intelligence Receptivity							
	*df*	*F*	*p*	*F*	*p*	*F*	*p*	*F*	*p*
Age	5	3.22	***<*** **0.05**	3.83	***<*** **0.05**	3.94	***<*** **0.05**	4.00	***<*** **0.05**
Robot	4	36.3	***<*** **0.001**	9.45	***<*** **0.001**	43.0	***<*** **0.001**	13.1	***<*** **0.001**
Age: Robot	20	1.81	***<*** **0.05**	1.06	0.38	1.02	0.43	0.47	0.97

#### Impact of Big Five Personality Traits

**Fig. 5:**
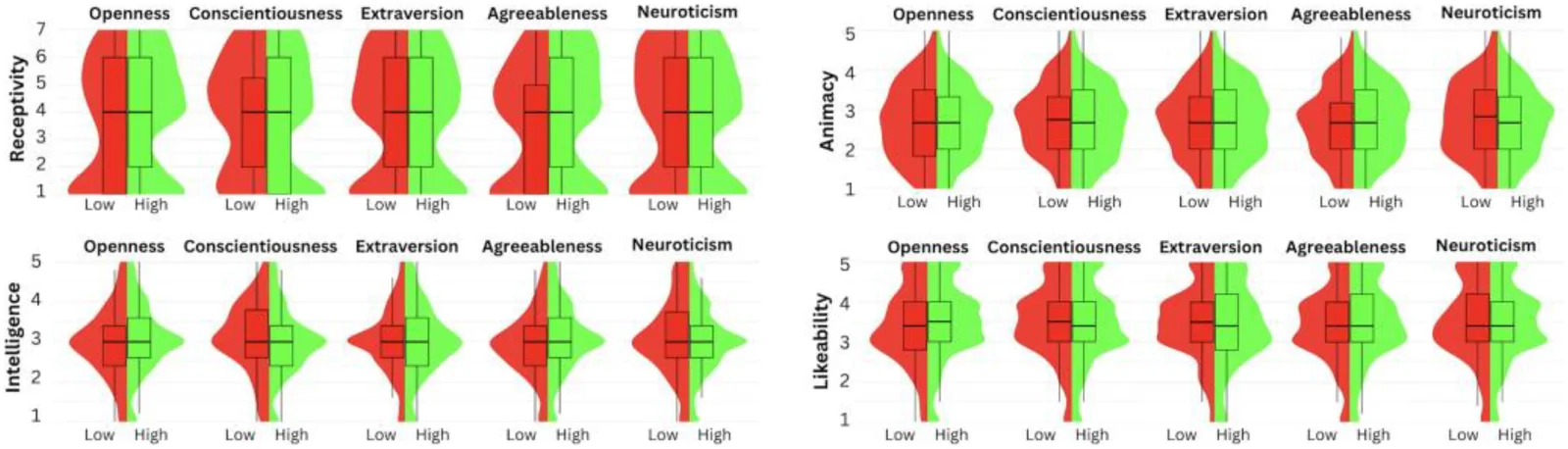
Violin plots displaying the distribution of robot perception and receptivity ratings grouped by low and high median groups of each Big Five personality trait. No significant differences between low and high groups were found

In order to explore the impact of personality traits within our sample, we first binned openness, conscientiousness, extraversion, agreeableness and neuroticism ratings into high and low categories using a median split [65]. The distribution of robot perception and receptivity scores for low and high splits of each trait are shown on Fig. 5.

To assess if any of these traits had a significant impact on robot perceptions, we then conducted four non-parametric two-factor ANOVAs for each trait to search for main effects or interactions with different robots. We found no significant effects, suggesting that Big Five personality traits have no clear impact on robot perceptions or receptivity to them.

#### Impact of Propensity to Anthropomorphism

**Fig. 6:**
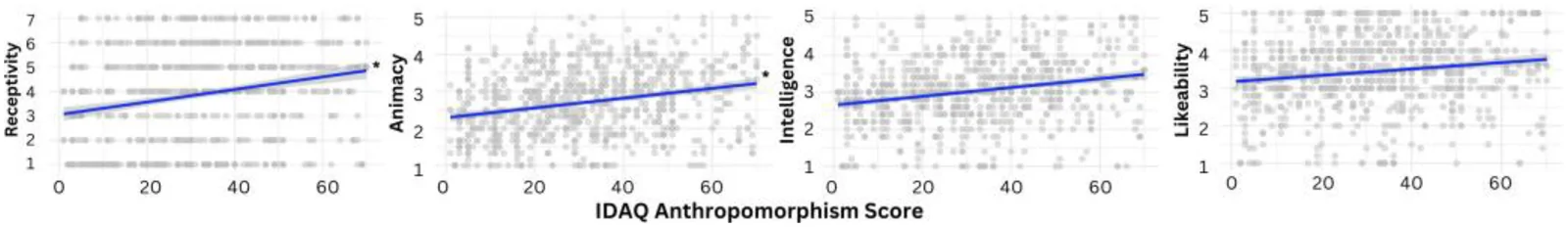
Line and scatter plots showing the distributions of robot receptivity and perception scores across different IDAQ scores. IDAQ scores had significant positive correlations with animacy and receptivity

Four two-factor ANOVAs were used to explore if IDAQ scores had main effects or significant interactions with different robots on robot ratings. The distribution of these scores and lines of best fit are shown on Fig. 6. We found that IDAQ scores had a main effect on animacy (*p* < 0.05) and receptivity (*p* < 0.05) ratings, as well as a significant interaction effect with different robots on likeability (*p* < 0.05).

*Post hoc* correlation analysis found a significant positive correlation between indi-vidual propensity for anthropomorphism and perceived robot animacy (*p* < 0.001), accounting for approximately 6.2% of variance (*R*^2^ = 0.062). Similarly, we found a significant positive association between IDAQ scores and receptivity to robots as companions (*r*(813) = 0.21, *p* < 0.001), explaining approximately 4.3% of variance (*R* = 0.043). To further explore the interaction effect we conducted a simple slopes analysis, which found that IDAQ scores significantly predicted likeability ratings for both zoomorphic robots, Paro (β = 0.009, *p* < 0.05) and Petit Qoobo (β = 0.010, *p* < 05).

#### Impact of Negative Attitudes Towards Robots (NARS)

To understand how an individual’s negative attitudes towards robots may specifically predict their perceptions of different robots as domestic companions, we searched for main effects or significant interactions of each NARS subscale per each Godspeed scale and receptivity to robot companions, resulting in 12 total two-factor ANOVAs (3 subscales x 4 dependent robot ratings). Results are shown in Table 4.

**Subscale 1 (Robot Interaction Situations)**: We found no main effect on our dependent variables of subscale 1, which measures negative attitude toward situa-tions that involve interacting with robots. However, we found significant interactions between this subscale and the robot presented on animacy and likeability (*p* < 0.05). Digging deeper via single slopes *post hoc* analysis revealed that higher scores signifi-cantly predicted lower animacy scores for three robots: Cozmo (β = −0.036, *p* < 0.05), NAO (β = −0.037, *p* < 0.05) and Petit Qoobo (β = −0.064, *p* < 0.001). Regarding likeability, higher scores predicted lower likeability for NAO (β = −0.061, *p* < 0.05) and Petit Qoobo (β = −0.063, *p* < 0.05).

**Subscale 2 (Robots in Social Spaces)**: This subscale, which measures negative attitudes toward robot influence on social spaces, had a significant main effect on robot likeability, intelligence and receptivity ratings. *Post hoc* correlation analysis showed a small negative correlations with participant ratings for robot intelligence (*p* < 0.05, *R* = 0.013), robot likeability (*p* < 0.001, *R* = 0.040) and receptivity to robots (*p* < 0.001, *R* = 0.031). Additionally, we found a significant interaction on likeability between Subscale 2 and the robot presented. *Post hoc* analysis found that higher scores predicted lower likeability ratings for NAO (β = −0.065, *p* < 0.05), Paro (β = −0.065, *p* < 0.05) and Petit Qoobo (β = −0.061, *p* < 0.05).

**Subscale 3 (Emotional Robot Interaction)**: This final subscale measures negative attitudes toward emotional robot interaction and had a significant main effect on animacy, likeability, intelligence and receptivity. *Post hoc* analysis showed that higher scores had small but significant negative correlations with animacy (*p* < 0.05, *R* = 0.059), intelligence (*p* < 0.05, *R* = 0.033), likeability (*p* < 0.001, *R* = 0.066) and receptivity (*p* < 0.001, *R* = 0.048), explaining an average 5.1% of variance

**Table 4:** Main effects and interactions of the three NARS subscales and robot presented on animacy likeability, intelligence and receptivity ratings

Factor		Animacy Likeability Intelligence Receptivity							
	*df*	*F*	*p*	*F*	*p*	*F*	*p*	*F*	*p*
NARS S1	17	1.35	0.17	1.65	0.06	0.85	0.64	1.42	0.14
Robot	4	31.8	***<*** **0.001**	8.81	***<*** **0.001**	36.5	***<*** **0.001**	11.1	***<*** **0.001**
S1:Robot	68	1.50	***<*** **0.05**	1.66	***<*** **0.05**	1.00	0.47	1.28	0.07
NARS S2	17	1.52	0.09	2.89	***<*** **0.001**	2.18	***<*** **0.05**	1.99	***<*** **0.05**
Robot	4	45.3	***<*** **0.001**	12.6	***<*** **0.001**	51.1	***<*** **0.001**	16.7	***<*** **0.001**
S2:Robot	68	1.12	0.23	1.50	***<*** **0.05**	0.91	0.67	1.09	0.29
NARS S3	12	2.59	***<*** **0.05**	2.23	***<*** **0.05**	2.17	***<*** **0.05**	2.31	***<*** **0.05**
Robot	4	36.9	***<*** **0.001**	10.3	***<*** **0.001**	43.7	***<*** **0.001**	14.3	***<*** **0.001**
S3:Robot	48	1.08	0.32	1.20	0.18	0.91	0.64	0.92	0.62

#### Impact of Pet Attachment (PAQ)

Finally, we conducted eight two-factor ANOVA to investigate if either subscale of the PAQ, avoidance or anxiety, had a significant main effect on any of the four dependent robot ratings and whether this interacted with robot type. Variance in avoidance subscale scores had no impact on robot ratings. However, anxiety subscale scores had a significant interaction with the robot presented on perceived animacy (*F* = 1.47, *p <* 0.05), likeability (*F* = 1.40, *p <* 0.05) and intelligence (*F* = 1.31, *p <* 0.05). Specifically we found via *post hoc* analysis that participants with higher anxiety about pet wellbeing and absence gave significantly higher animacy (β = *−*0.014, *p <* 0.05), likeability (β = *−*0.016, *p <* 0.05) and intelligence (β = *−*0.016, *p* < 0.05) ratings to Cozmo, and higher animacy scored for Petit Qoobo (β = −0.015, *p* < 0.05).

### Qualitative Themes of Participant Rationale

#### Theme 1: The Philosophical Divide Between Real and Artificial

A notable near-even philosophical divide in robot receptiveness emerged between respondents. While a slight majority (54%) of respondents were either currently recep-tive to these robots, based on their audiovisual impressions, or felt robots could become more suitable with requisite improvements (discussed in Theme 2 and Theme 3), 46% of respondents reported that social robots’ nature as artificial, programmed and simulated made them inherently unsuitable, unworthy or uninteresting as domes-tic companions. Rationale for this concern manifested in many ways. Some expressed a biologically essentialist view, stating that robots are *“just electronics”* (P1), *“just a machine”* (P101), or *“still a bunch of wires and circuits”* (P129). The nature of robots as programmed entities, rather than having true agency was also a key concern among these participants, with P44 stating *“a robot doesn’t have emotions or inten-tions”*. For P33, this lack of sentience meant “the relationship with them from my perspective is not really meaningful”, while P148 felt that *“robots should not replace real-life”*. Contrasting robots and real pets threw these issues into sharp relief, with P11 stating *“one is alive and the other is a robot”* and P124 writing *“no, a pet has to be real and living”*. P108 related this back to the agency, writing that a *“real pet is deliberate and gains a relationship, the robot is programmed or made to do so”*. These comparisons also touched upon the meaning and value lent to real pet relationships by their organic lifecycles: *“you will experience real heartbreak if you outlive it. So the emotional rewards that I feel have stronger meaning”* (P159). Finally, in terms of emo-tional responses to this concern, these respondents were again divided. P28 seemed almost regretful after rating their receptivity to Paro as 2 on the 7-point scale: *“I think I’d have a hard time escaping the ”machine in here” feeling. It does look cute though”*. Some respondents had extremely negative responses, such as P20 on Paro:

I can’t find words to describe how awful this thing is. A total waste of time and resources. Vomit inducing tat. A real pet is real, with real emotional connection and a living, breathing thing. A robot is a very poor mechanical substitute. Why choose fake when you can have real? - P20.

Meanwhile, P155 summarised almost all of these philosophical concerns in one comment on Petit Qoobo:

The point of companionship is the connection and mutual bond with another living crea-ture. The idea of trying to mimic this with a robot is genuinely horrifying to me. Something that you can switch off, or program to your preferences and that cannot have any sem-blance of autonomy and comes with no moral responsibility should never be described as a companion. These are toys at best, or sociopath creation devices at worst.

Overall, our findings indicate that approximately half of users surveyed may currently hold non-negotiable philosophical concerns about robots as domestic com-panions or companions, which may be difficult to address by technical or design improvements.

#### Theme 2: Current Receptiveness to Robot Companions is Driven by Appealing Form, Interactivity and Lifestyle Fit

Respondents who were already receptive to the robots shown as pet alternatives primarily reported one of three key factors in their rationale: form factor, interactivity and activities they could share with it. For Cozmo and both zoomorphic robots, their form factor was a key aspect of their appeal. Some described a robot as cute, such as P50: *“Paro is very cute and could see it as fun household pet”*, and P63 described Petit Qoobo as *“so cute and lively”*. P46 further linked cuteness to evoking animals:

Paro is very cute!!! It is almost the same as real animal!!! She is very smart and sensitive as when we touch her head, she will close her eyes, It looks very real to me - P46.

Others also noted the importance of evoking animals, such as P77 who wrote *“since it looks more like an animal/pet, I feel like it can be a companion like a pet”*. Not all participants felt this was the ideal form factor, however, with P158, writing that Paro *“might be an uncanny valley due to its closer resemblance to a real pet”*. P3 raised further nuance while discussing Petit Qoobo *“it does not try to look like a specific animal, so no uncanny valley”*. Cozmo’s mechanical appearance had its own appeal for P16, who felt it was *“very charming, and not trying to emulate a real creature”*, and for P96: *“I love the little mechanical guy, he’s a funky little being”*.

The second key factor that drove initial receptivity was the potential for interac-tion. This was often related to the two most complex social robots, Cozmo and NAO. P17 felt Cozmo was *“play companion and for fun interactions”*, while P164 wrote that NAO *“has a life of its own as it is able to respond, see, and interact back”*. P140 related their perceived cuteness and interactiveness of Cozmo to their receptiveness, writing: *“a lovely cute wee companion to have. I’d love one of these guys around the house, so many ways to interact with them and they really seem like they’re learning as you get to know them”*. The two zoomorphic robots were also discussed in rela-tion to how they evoked animal interaction. Regarding Petit Qoobo, P156 responded that“*I would like the idea of having one around, not for any particular purpose, but more like a pet I would interact with*”, while P46 noted that *“when we touch Paro’s head, he closes his eyes as [a] real animal”*. This led to the final factor participants cited when discussing the positive reception of robots: envisioning interaction in their daily lives. This could be in the home, as described by P18: *“I am very receptive to Paro as a household companion that serves as a form of easy pet, something to cud-dle and keep on your lap”*, while P9 wrote that they *“love to take him [Cozmo] into university and study with him on my desk”*. However, 84 respondent statements were coded with ’For Specific Users’, indicating the user felt only the robot was suitable for specific users, most often as toys for children, rather than themselves. There were also those that related it to specific life circumstances, such as P57: *“if I was living alone I could imagine it being a nice tactile experience when I am craving a hug or physical company e.g. while watching a movie’* ’.

#### Theme 3: Increasing Robot Agency, Affection and Fidelity are Key Factors for Future Receptiveness to Domestic Robot

While 54% of respondents did not feel that these robots were inherently unsuitable as companions, it was still more common for the viability of these robots to be ques-tioned rather than accepted. We observed three key categories of improvements that participants suggested were needed to bridge this gap: agency, affection and fidelity. First, those with experience of real pets contrasted the *“free will”* (P91) of animals with the perceived lack of agency in robots, with P31 stating that *“a real pet feels things and can act autonomously”*. P9 related further agency to unpredictability, writ-ing: *“Real pets are unpredictable and are living beings who are trusting humans with their care. Robots are likely more predictable but where’s the fun in that?”*. This desire for unpredictability was also felt by P85, who wrote that robots should have *“unin-tentional movements like real pets. Sometimes they have, like, sudden emotion spikes I feel that are nice to ensure that it is more fun to have around, and to really feel that it is around not waiting for us to interact with it”*. This concern about relation-ships with robots being more one-sided was echoed by others, with P28 writing that *“real pets don’t always want interacted with”* and P48 noting that *“a robot pet can be switched off”*. P119 commented on the value of responsibility in relationships: *“I feel less responsible for the robot pets. I feel like I can interact with them only whenever I want to, whereas a real pet is an alive companion which I need to take care of no matter what my needs or conditions are.”*.

Based on their impressions from the audiovisual survey, many respondents were unconvinced by the robots’ capacity to show *“empathy and affection”* (P25) and sought an affective relationship that *“can evolve and change”* (P93), rather short-term inter-actions that feel *“gimmicky”* (P7). For example, when discussing NAO P115 wrote that *“despite being an incredible piece of technology, it lacks the warmth and affection I would want from a companion”*. This was contrasted with the affection and social outreach offered by real pets, highlighted by P6: *“she would always be happy to see me”* and P11: *“when I get home she’ll be there waiting for me and gives me joy when I open the door and we have are both so pleased to see each other”*. For P106 this was a key feature, writing that robots could serve as pet substitutes *“when it comes to me just to be with me”*.

Finally, participants expressed a need for the robots presented to have more appeal-ing physical attributes and improved capabilities. A prominent motivator for this was the desire for soft, affecting touch, demonstrated by P17: *“addressing the warmth and softness of a companion robot would be good”*, and P2: *“cuddling with a soft robot would be nice”*. Increased reactivity during social touch was also mentioned: *“respond-ing to touch with affectionate gestures. This would provide companionship and stress relief”* (P5). Meanwhile, the current level of physical fidelity can reinforce the view that *”robot pets mostly come off as toys rather than pets”* (P123). A particularly desir-able element among respondents was improving robots’ capacity for independent *“fluid movement”* (P75), echoing findings by Sasser et al. [21]. When considering the static Petit Qoobo, P161 wondered if *“something that combines the cuteness of the Qoobo with the motility of a Roomba might be a winning formula. It’s not a real animal, but it could be a real pet. Sort of.”*.

#### Theme 4: Robots Framed by Practicality and Purpose

This survey explicitly asked participants to rate the five robots as pet alternatives, based on their early impressions. While not universal, participants prominently framed robots around the utility they may offer, which was reflected by the single most preva-lent qualitative code - *Useful/Serves a Practical Purpose*- which P130 exemplified when writing *“robots are machines, tools, not sentient beings”*. Perceived usefulness was the primary rationale for why users were more receptive to Roomba than the other robots: *“Roomba cleans, which is something I would rather not do”* (P21), even though it was often seen as *“hardly a companion”* (P25) or *“there isn’t anything to form a bond with”* (P50). Despite being viewed primarily as a functional object, there were respondents who nevertheless found themselves emotionally attached to Roomba. This could be prompted by various means, such as via individual anthropomorphisa-tion: *“it’s a cute wee robot, knows how to do its job very well and would be nice having it wandering around. It doesn’t speak but I’d probably speak to it anyway, like say* ”Hi Roomba” *when I saw it going about its business”* (P140). It could result from wider cultural influence: *“the pervasiveness of memes surrounding Roombas has habituated me slightly to the idea of the Roomba as a living creature”* (P140). P28 described facil-itating this through personalisation: *“I can see myself putting stickers or something on it to make it feel more friendly”*.

Roomba was also not the only robot evaluated in a utilitarian way, as respondents described various practical purposes that the social robots could fulfil. P123 noted that Cozmo could be an educational asset, writing that ‘*“seems like a good tool to introduce kids into coding”*, while P57 speculated how NAO assist with *“daily life tasks”* such as *“reminding me of tasks to complete or making lists”*. Providing companionship itself was also viewed through a utilitarian lens by some respondents, such as P43: *“it could be useful sometimes to not feel completely alone”*. This lens also drove concerns about robots perceived to lack utility, illustrated by comments on NAO by P11: *“what is the purpose of it”*, and P20: *“it’s another glorified toy. Completely useless. It can’t do anything useful for me. A time waster”.* In these cases, the robots may be viewed as static objects or *“more of a decoration”* (P5).

Respondents also framed the robots within their applicability to specific user groups, particularly children or elderly users, reflecting applications from prior research [8, 38].

I am not sure I am ready for this type of interaction, however, I could see the benefits for children and perhaps the elderly. - P88.

Participants often related the robots they found toy-like to child use. For example regarding NAO, P42 wrote *“for a child it would be great toy”*, while P157 wrote that Paro *“would be great for kids, friendly and fun”*. P100 speculated that robots *“could be teachers for young children or companions for elderly people in specific circumstances”*, relating this to an experience of *“bringing my dog to visit my elderly grandmother in a care home”*. Similarly P30 wrote that Paro *“would be perfect for older and lonely people”*. This prospect deeply concerned P155, however: *“the idea that would farm out the emotional labour of caring for vulnerable people to a machine is emblematic of everything that is wrong with late capitalist society”*.

Framing the presented robots through their practicalities also provoked compar-isons with real pets. Several participants speculated that robot pets would be useful for those who cannot keep pets, due to for example *“working hours”* (P11) or *“if I lived alone in a place where I could not have a pet, or adequately care for one”* (P159). P23 highlighted another consideration of caring for a pet versus a robot, noting that *“the legal responsibility for a real pet is not a joke and can have legal consequences”*. The most positive of these comparisons viewed robots as accessible ways to benefit from pet-like interaction: *“it could represent all the good things about a pet, the love and affection it could give you but without the person needing the responsibility of actu-ally taking care of a pet”* (P101). Furthermore, robot pets could circumvent anxieties around pet health, as described by P24: *“[I] felt horrible and helpless when they were ill”* and P94: *“I would have a mental breakdown if my pet dog would get sick […] my days would be ruined and I would near about throw up from stress”*. This is not a strict upside, however, as others described deriving specific value from the nature of car-ing for an organic living pet: *“if he’s been unwell and we’ve cared for him, he always becomes more affectionate for a time. That specific behaviour is quite touching”* (P33).

Consideration of robot practicalities also manifested in technological and privacy anxieties. Regarding privacy, P41 described Cozmo as a *“privacy and security nightmare”* and P26 concurred, writing that *“people won’t think their* “pet” *could prove to be a hazard with an internet connection, facial recognition, and visual data about its environment”*. Beyond this specific concern, however, P114 expressed a wider dislike of technological change that these robots represented:

I really hate the idea of AI and robots becoming part of our day-to-day lives. While it could be seen as a natural progression of mankind’s … well, progress, there is something in me that truly detests all of this technology, full stop. It’s not interesting or cool to me, just uncomfortable and undesirable. - P114.

## Discussion and Implications on the Future of Domestic Robot Companions

Following analysis of our results, we present eight key takeaways that contextu-alise our findings with those of prior work and provide actionable considerations for robot designers and researchers regarding how domestic robots may be perceived by prospective users.

### Individual Differences that Predict Receptivity to Domestic Robot Companions

Our findings indicate that a user’s propensity towards anthropomorphism and nega-tive attitudes towards these robots predicts their early perceptions of robots as pets. While not unexpected, demonstrating this relationship for robot pets could allow future studies to screen participants and better interpret results. It could be considered surprising, on the other hand, to observe no significant differences in the perceptions of robots based on personality traits. For instance, high neuroticism has been shown to be associated with higher technology anxiety, which may have predicted lower recep-tivity [66]. While prior work has demonstrated that personality traits can impact the perception of a single robot explicitly exhibiting their own personality traits [33], we find no evidence that these traits impact initial perceptions of different morphologies, or receptivity to robots as domestic pets or companions.

We did find interactions between individual differences and robot morphology that motivate further investigation. Regarding pet experience, prior work is inconclusive. Both positive and negative correlations between pet experience and robot perception have been observed, with possible explanations including evoking past experiences [67], or worse evaluations resulting from higher user expectations [68]. Our survey found that pet attachment correlated with the perceived animacy of specific robots: the mobile and active mechanomorphic Cozmo, and the abstract zoomorphic Petit Qoobo.

We observed that younger adults were significantly more receptive to all four social robots and rated them as having higher animacy, likeability and intelligence. While this finding bears similarity to earlier results on social robots [69], such as Kertesz et al.’s exploration of long-term users of Sony’s domestic dog robot AIBO, which found that younger users rated the capabilities of these robots as higher, while older users regarding them more often as toys [70]. This has important implications for the adoption of robots as domestic companions, as currently research in this area is still heavily weighted toward socially assistive applications in elderly care settings. However, the lower upkeep costs, greater practicality, and ability to keep robots even when living as a tenant, could be significant adoption factors for younger users^9^, which we argue warrants further investigation of this use case.

### Importance of Apparent Intelligence, Independence and Adaptation

Our findings suggest that improving the ability for these robots to convincingly convey personhood, affection, independence, and long-term emotional growth are key targets for future domestic robot adoption. This reinforces prior work reporting that partic-ipants felt that robots possessing “*thoughts, feelings and emotions*” were essential to their acceptance in domestic environments [71], and that existing robots suffered from becoming *“repetitive”* or *“boring”* over time [70].

Our results highlight multiple avenues for robots to display these attributes prospective robot users. Facilitating expressive and independent movement shows strong potential in conveying internal agency and intelligence. Even Roomba, which features little to no social interaction, but does perform independent movement, received higher intelligence ratings than all four social robots. Independent and expres-sive movement would, alongside improved emotional expression capabilities, facilitate the ability for robots to initiate social interactions with users, another prominent rewarding pet interaction that participants raised. Free movement better enables robots engage in *social proxemics*, whereby information, affinity and interaction are conferred between social partners based on their physical distance and position from each other [72]. The importance of this effect has been investigated between humans and animals, with Briones et al. finding that social distance correlates with assessments of trust and aggression between humans and dogs [73], and has also been found to mediate user comfort, perceptions of intelligence and social affinity in Human-Robot Interaction [47, 74].

How best to emulate pet-like emotional intelligence, personality, and long-term adaptation is also still an open question. One approach could be to focus on the development of pet-like Computational Models of Emotion (CMEs). The majority of existing CMEs for AI agents are designed to emulate human-like interaction and intelli-gence for AI agents [75]. Therefore, significant uncharted space remains to explore how CMEs can be designed that emulate and exhibit pet-like intelligence and interaction, with limited existing examples [76]. Alternatively, the emergent nature of Generative AI models could be used to simulate unpredictable but understandable pet-like role-playing, in a similar manner to how recent work have explored using AI agents to role-play as household objects [77, 78].

### Philosophical Divide may Limit the Receptivity to Robots as Domestic Companions

Our results suggest the existence of a near-even philosophical divide in initial robot pet receptivity across respondents that may limit widespread domestic adoption of social robots similar those presented in the survey. Only 50.9% of respondents felt they could potentially share pet-like experiences with robots, while 46% of respondents commented that robots could never be truly suitable for this role due to their artificial nature as simulations. This constitutes a barrier to these robots serving hedonic utility for these users, such as emotional support or companionship. Instead, they were only seen as useful if they served a practical purpose or acted as toys for children. As such, this survey suggests that robot designers may expect at most just over 50% of users with cultural backgrounds similar to our sample to be currently open to adopting such social robots as pet alternatives, based on early impressions.

While the objections of some sceptical respondents was grounded in the innate nature of robots as not a living being, many others cited that the key elements they felt were robots could not possess were genuine personality, intelligence, agency or affection. This represents a call to action for designers, as it remains to be seen if this objection will hold or erode as the apparent emotional capabilities and intelligence of social robots improves. Advancements in the fidelity of conversational agents has been shown to drive increased anthropomorphism of those systems [79, 80], and we may similarly expect that improving the ability for robots to express evolving, independent personalities and naturalistic pet-like interactions may change current perceptions and increase the proportion of receptive users. Finally, our work revealed initial indications of how familiarity with Roomba, through social exposure and online content, improved user receptivity. This effect that would be valuable for future work to further explore.

### Practical or Hedonic Usefulness is Key to Robot Perceptions

Users most commonly viewed the five social robots through a utilitarian lens, in line with their etymological roots (‘*forced labour* ’ in Czech, 1920), and echoing prior work [19]. This utility manifested in various forms, including their potential practical house-hold uses, reflected in the high levels of receptivity for the Roomba as a cleaner, or Cozmo as an educational or entertaining toy for children. Others valued possible hedo-nic utility that robots like Paro or Petit Qoobo could offer through their visual appeal, soft-touch interaction, or evocation of positive pet experiences.

**Fig. 7:**
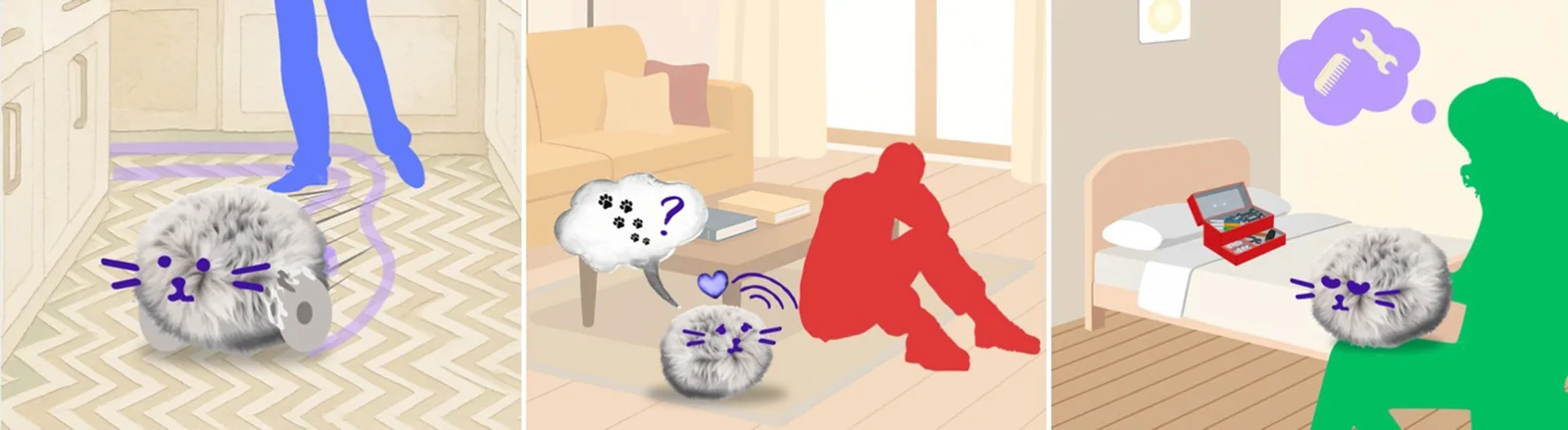
Concept illustrations of three key requirements and design challenges for future domes-tic robot companions, drawn from our findings. Left: Expressive, independent movement using mechanomorphic elements. Centre: Emotional intelligence, engagement and encouraging positive lifestyle choices. Right: Encoding play or maintenance as accessible nurturing interactions

Being understood and evaluated in terms of utility is not necessarily an barrier for robots to serve as domestic companions or pets, however, as real pets are also often viewed in this way. Historically, pets served as practical companions, while in mod-ern times they serve hedonic uses by support psychological needs such as autonomy, competence, and relatedness [1, 81]. Furthermore, our respondents discussed how pets provided them with routine, exercise, supportive emotional interaction, and helped teach them responsibility. This echoes the patterns of virtual pet use identified by Lin et al., who observed some users seeking practical collaboration, while others prioritised believable social interaction [51]. Robots have the potential to act as pet alternatives if they can offer similar practical or emotional utility to users. Future work that designs and evaluates ways for robots to offer pet-like utility in domestic environments would be valuable (see Fig. 7), such as recent research by Bork et al. [82] that explores how zoomorphic robots can encourage user activity through pet-like social intrusions. How-ever, designers may wish to be cautious of robot utility that aligns too closely with existing unemotional appliances, as this may limit user preconceptions of a robot’s domestic role, with Roomba often framed as a tool rather than a viable companion by respondents. Indeed, a prior study of long-term AIBO users found these users primar-ily sought for their robots to offer emotional bonding and social interaction, rather than act as gadgets or assistants [70].

### Robot Morphologies Confer Perceptual Trade-Offs Between Capability and Social Affordance

While zoomorphic designs strongly communicate pet-like social affordances, we found the expressive independence offered by other morphologies was a distinct advantage when driving positive early perceptions of robots as domestic pets or companions. Per-ceptions of robot animacy, intelligence and likeability were broadly neutral across robot morphologies, with the exception of Cozmo (high animacy and receptivity), Roomba (low animacy but highest receptivity) and Petit Qoobo (low receptivity). While the two zoomorphic robots were highlighted by some respondents for their visual appeal or ability to evoke pet interaction, we did not observe the preference for this morphology seen in earlier studies [10, 21]. This may reflect the higher expectations of everyday users held for robots to exhibit independence and interaction in domestic settings, when compared to applications of zoomorphic robots in care settings. This presents a challenge for designers, as while zoomorphic form factors are effective at commu-nicating pet-like social affordances, they place limitations on the robot’s capabilities, in particular obstructing expressivity and independent movement. Participants placed strong value on these capabilities in real pets and repeatedly made note of these appar-ent limitations during their initial impressions of these robots. By contrast, Cozmo’s mechanomorphic form factor allows it to make full use of expressive screen-based faces and the mobility granted by its wheels to facilitate expressiveness, independent move-ment and apparent agency, all of which motivated positive early perceptions (see Fig. 7). However, mechanomorphism also came with a crucial trade-off, as both Cozmo and NAO were routinely perceived as toy-like, rather than credible pet alternatives. The best way to bridge these two morphologies to capitalise on the benefits of both remains unclear. This presents a challenge for future designers to explore whether hybrid morphologies can effectively reconcile expressivity and capability with famil-iar pet elements, as well as how to best align robot form factors with diverse user expectations.

### Conflicts of User Control and Companionship

Personalising the appearance or behaviours of social robots has previously been high-lighted as an effective way to drive their adoption and acceptance [83, 84]. In particular, customising utilitarian robots with zoomorphic social affordances has shown potential, with past work demonstrating how the addition of animal-like visuals, or conceptual-ising functions like charging as ‘feeding’, can facilitate pet-like interaction with robot vacuums [31, 85]. This was also echoed in our work, as while respondents primarily viewed the functional robot Roomba through a lens of practical utility, some expressed the desire to treat it as a social entity and enhance this through visual customisation. Unlike utilitarian robots, however, where customisation may enhance social engage-ment for utilitarian robots, our findings suggest attempts to personalise robots may instead undermine their perceived authenticity and agency. Our respondents rarely showed desire to customise social robots, and instead expressed concerns that robots built to adapt to their user’s preferences and needs would result in less meaningful and ‘one-sided’ relationships, noting that real pets are real, unpredictable living beings, while robots lack free will, existing so much at the behest of the user that they ‘can be turned off’. As reflected in prior work on believable agents [86], not allowing robots to deviate from user control or express dysfunction and flaws could erode their ability to express convincing personhood, independence and agency, which our participants repeatedly identified as key requirements for robot pets to convincingly adopt pet-like roles. This departure from prior results may reflect the experience gap between embodied interaction and our participants initial audiovisual perceptions. However, we advise designers to use caution when importing existing personalisation approaches from social or utilitarian robots to pet-like robots. Instead, the critical design chal-lenge is to understand how personalisation can coexist with the critically important user perceptions of independence and unpredictability. We call on future research to explore these implications and if a virtuous balance can be found.

### Practicality Makes Robots More Accessible, But Less Pet-Like

Our findings highlight a paradox: while robots are free from the obstructive burdens of care that real pets need, respondents perceive that the absence of these responsibilities may erode what makes pet relationships valuable for owners. A primary advantage robots could have as domestic pet alternatives is that their practical advantages could make them accessible when compared to real pets, particularly for users who face financial, mobility or housing restrictions. However, respondents emphasised that the finite lives of pets, the responsibility of caring for them and improving their quality of life are all crucial factors that drive emotional connection and enjoyment during pet ownership. This also aligns with the effort heuristic, whereby people assign higher value to interactions, objects or relationships that require greater personal effort and investment [87]. These caring responsibilities also supported users in maintaining ben-eficial lifestyle elements, such as routines, exercise and social links. Nevertheless, there are people who cannot keep pets for whom the lower maintenance requirements of robots could be very beneficial. This poses a challenge for future work to reconcile these two competing demands by exploring how to design optional care tasks that foster a sense of responsibility for robots without undermining their practical benefits for users who need them (see Fig. 7). A promising direction from recent research sug-gests that the act of repairing or maintaining social robots can strengthen the user’s relationship with them [88], although the accessibility of these actions depends on the robot, task and user.

### Robot Pet Challenges Motivate Accelerating Design and Development Exploration

Many respondents, even when not opposed to the concept of domestic robots, empha-sised that unless they can move, interact and express themselves with the more apparent autonomy and emotional depth, they perceive them as unsuitable as domes-tic pet substitutes. Respondents raised the need to improve current robots’ ability to move and act independently and expressively, interact with their environment and hold believable, evolving emotions, echoing findings from prior work with affective agents [51, 71, 89, 90]. For many robots, achieving this would require significant exploratory design, iteration and evaluation of robots’ affective displays, interaction, movement and intelligence. This represents a significant challenge, as building and modifying robots is a technically obstructive process requiring significant hardware and software expertise. In order to accelerate progress and innovation in the field of domestic robots, we recommend that designers and researchers to adopt novel methodologies that allow for the rapid prototyping of these robot. One promising approach is utilising virtual reality simulations [91, 92] or extending the capabilities of existing robots with aug-mented reality [76, 93] in order to quickly trial new affective expressions, interactions or emotion modelling without disrupting the robot’s appearance or requiring physical modification.

## Limitations

We utilised a survey in order to better observe trends in individual differences and to capture a large number of participant perspectives on robots as domestic companions. As a result, we designed this work to capture participants responses and judgements of robots based on videos, images and descriptions of these robots. However, it does not capture participants impressions of face-to-face interaction, in which elements of the robots’ physical embodiment would impact perception (e.g., presence, movement, touch), making robot attributes such as intelligence difficult to accurately assess. Thus, our findings can only be understood as a reflective of the factors that drive initial impressions and receptivity to these robots, and user perceptions following lived lon-gitudinal experiences of these robots in a home environment may differ. Furthermore, while we investigated perceptions of varied robot morphologies in this work, we chose to set the number of robots to five to control survey completion times, limiting the direct applicability of our findings to these robots.

We chose to distribute the survey across the networks of three universities, one in the United Kingdom, one in Canada and one in Thailand, with the aim of observing cultural differences. However, logistics and snowball sampling resulted in a dispropor-tionate subset of British participants (66%) (see Table 1), which limits the applicability of these results across international samples. This is significant as past works have shown that different cultural samples can have significantly different perceptions of social robots [25, 94, 95]. Additionally, while we administered the NARS to assess par-ticipants’ preconceptions towards robots, we did not observe technology and media literacy due to scope, which could have further impacted participant perceptions or contextualised their rationale, as suggested by prior work [96].

The majority of our respondents (84%) had current or pet experience. While this was not unexpected, as data suggests 62% of households in the UK own pets^10^, our findings may be less representative of populations with lower pet experience. Finally, we recruited a sample of 163 participants and used shorter versions of established tools, such as the BFI−10 and three of the five Godspeed scales, both of which should contextualise our findings as more exploratory in nature, rather than conclusive. Our choice of measures was made in order to maintain the length of this survey at ≈ 20–25 min to avoid fatigue and reduce aborted submissions.

## Opportunities for Future Work

There are several clear ways in which future work could expand upon the scope of this work in order to learn more about the factors that guide users’ preconceptions and impressions of domestic social robots. First, it would remain valuable for future surveys to utilise strict cross-cultural sampling in order to provide a clearer under-standing of how the relationship between individual differences and robot preferences varies across cultures. This sampling could also expand the demographic and individ-ual factors recorded and analysed to include technology literacy, media literacy and more detailed socio-economic and cultural differences. Second, additional robot mor-phologies could also be explored to paint a more complete picture of user receptivity to different robots, particularly robots such as Miro or AIBO that blend zoomorphic and mechanomorphic forms. Third, if logistically possible, it would be valuable to increase inferential power while exploring this topic by administering further scales to assess participant perceptions or individual differences, such as the full-length Big Five Inventory, and by recruiting a larger and broader sample. Finally, it would be valuable for future work to compare findings from the broader sample in this survey with those of smaller follow-up studies that involve user in-person robot interaction and longitu-dinal real-world use, to understand if and how early impressions and preconceptions correlate to lived user experiences.

## Conclusion

Domestic robots could serve as an accessible pet alternative and provide emotional support and lifestyle benefits for individuals who choose not to keep pets for logis-tical or personal reasons. However, they have failed to achieve widespread adoption, and it is unclear what factors drive individual receptivity and perceptions of different robot pets. To address this gap and support designers in developing and deploying robot pets, we presented a mixed-methods survey of 163 participants to investigate individual differences in user perceptions of a set of five robots varied by morphology, as well as a thematic analysis of user rationale. Age, pet attachment and individual propensity to anthropomorphism were found to correlate with positive perceptions of social robots as pets, while the Big Five personality traits had no significant effects. A philosophical divide emerged in users’ rationale for these perceptions, as nearly half of the respondents held non-negotiable objections to robot pets based on their inherent artificiality. We detail which attributes of the presented robots drove cur-rent receptivity, and the key improvements needed for robots to serve as viable pet alternatives for everyday domestic users. Finally, we discuss the implications of the work on future robot research and design, highlighting tensions presented by robot morphology, practical accessibility and personalisation.

## Electronic Supplementary Material

Below is the link to the electronic supplementary material.

Supplementary Material 1: PDF copy of the survey administered in the study.

Supplementary Material 2: Qualitative Codebooks generated through analysis of participant responses.

Supplementary Material 3: Anonymised qualitative and quantitative data collected and analysed in the study.

## Data Availability

All data generated or analysed during this study are included in this published article and its supplementary information files. This includes the full anonymised dataset from the original survey in tall and wide formats, as well as formatted qualitative transcripts and category statistics.

## Footnotes

1 Qualtrics https://www.qualtrics.com/.

2 Paro https://www.paroseal.co.uk/.

3 Petit Qoobo https://qoobo.info/index-en/.

4 Cozmo https://anki.bot/pages/cozmo.

5 NAO https://us.softbankrobotics.com/nao.

6 Roomba www.irobot.com.

7 Gitnux - Robot Vacuum Industry Statistics https://gitnux.org/robot-vacuum-industry-statistics/

8 We took a fully qualitative approach to validity on advice from the creators of thematic analysis to not calculate numerical Inter-Coder Reliability [62, p.278], acknowledging the influence of the researcher on the process

9 UK Government Statistics show that 70% of 16–24-year-olds are private renters, falling to less than 20% by 45–54. https://www.gov.uk/government/statistics/chapters-for-english-housing-survey−2023-to−2024-headline-findings-on-demographics-and-household-resilience/chapter−1-profile-of-households-and-dwellings.

10 Statista - Share of households owning a pet in the United Kingdom (UK) from 2012 to 2026 https://www.statista.com/statistics/308235/estimated-pet-ownership-in-the-united-kingdom-uk/.

## References

[1] Gray PB, Young SM (2011) Human–Pet dynamics in cross-cultural perspective. Anthrozo¨os. 24(1):17–30. Publisher: Routledge eprint: 10.2752/175303711X12923300467285. Accessed 2025-01-07

[2] McConnell AR, Brown CM, Shoda TM, Stayton LE, Martin CE (2011) Friends with benefits: on the positive consequences of pet ownership. J Personal Soc Psychol 101(6):1239 10.1037/a0024506 21728449

[3] Polheber JP, Matchock RL (2014) The presence of a dog attenuates cortisol and heart rate in the Trier Social Stress Test compared to human friends. J Behav Med 37(5):860–867. 10.1007/s10865-013-9546-1 24170391

[4] Young J, Pritchard R, Nottle C, Banwell H (2020) Pets, touch, and COVID-19: health benefits from non-human touch through times of stress. J Behav Econ Policy 4(2):25–33

[5] McCarthy L, Simcock T (2025) Pets and private renting: a rapid evidence review of the barriers, benefits, and challenges. Int J Hous Policy 25(1):119–146. Publisher: Routledge eprint: 10.1080/19491247.2024.2308711. Accessed 2025-07-23

[6] Schwarz PM, Troyer JL, Walker JB (2007) Animal house: Economics of pets and the household. BE J Economic Anal Policy 7(1)

[7] Pomba C, Rantala M, Greko C, Baptiste KE, Catry B, Van Duijkeren E, Mateus A, Moreno MA, Py¨or¨al¨a S, Ruˇzauskas M et al (2017) Public health risk of antimicrobial resistance transfer from companion animals. J Antimicrob Chemother 72(4):957–968 10.1093/jac/dkw481 27999066

[8] Hudson J, Ungar R, Albright L, Tkatch R, Schaeffer J, Wicker ER (2020) Robotic Pet Use Among Community-Dwelling Older Adults. The journals of gerontology. Ser B Psychol Sci social Sci 75(9):2018–2028. 10.1093/geronb/gbaa119 PMC7566965 32789476

[9] Chang WL, Sˇabanovi´c S, Huber L (2013) Use of seal-like robot PARO in sensory group therapy for older adults with dementia. ACM/IEEE Int Conf Hum -Robot Interact 101–102. 10.1109/HRI.2013.6483521. ISBN: 9781467330558

[10] Bradwell HL, Edwards K, Shenton D, Winnington R, Thill S, Jones RB (2021) User-Centered Design of Companion Robot Pets Involving Care Home Resident-Robot Interactions and Focus Groups With Residents, Staff, and Family: Qualitative Study. JMIR Rehabilitation Assist Technol 8(4):30337. 10.2196/30337. Accessed 2025-06-26 PMC8593804 34723825

[11] Wada K, Shibata T (2007) Living with seal robots - Its sociopsychological and physi-ological influences on the elderly at a care house. IEEE Trans Robot 23(5):972–980. 10.1109/TRO.2007.906261

[12] Jeong S, Zisook M, Plummer L, Breazeal C, Weinstock P, Logan DE, Goodwin MS, Graca S, O’Connell B, Goodenough H, Anderson L, Stenquist N, Fitzpatrick K (2015) A social robot to mitigate stress, anxi-ety, and pain in hospital pediatric care. In: Proceedings of the Tenth Annual ACM/IEEE International Conference on Human-Robot Interaction Extended Abstracts - HRI’15 Extended Abstracts (1), pp 103–104 10.1145/2701973.2702028. ISBN: 9781450333184

[13] Foster ME, Ali S, Litwin S, Parker J, Petrick RPA, Smith DH, Stinson J, Zeller F (2020) Using AI-Enhanced Social Robots to Improve Children’s Healthcare Experiences. In: Wagner AR, Feil-Seifer D, Haring KS, Rossi S, Williams T, He H, Ge S, S. (eds) Social Robotics. Springer, Cham, pp 542–553. 10.1007/978-3-030-62056-145

[14] Borgstedt J (2023) Investigating the potential of life-like haptic cues for socially assistive care robots. In: Companion of the 2023 ACM/IEEE International Conference on Human-Robot Interaction. ACM, Stockholm Sweden, pp. 745–747. 10.1145/3568294.3579972. Accessed 2024-06-06

[15] Time - Sony’s New Aibo Robot Dog Is Nearly $3,000, But It Can Tell How You’re Feeling. https://time.com/5376649/sony-aibo-robot-dog-america/?utmsource=chatgpt.com. Accessed 2025-08-30

[16] Katsuno H, White D (2022) Haptic creatures: tactile affect and human– robot intimacy in Japan. In: Consumer Culture Theory In Asia, pp. 242–262 10.4324/9781003111559-18

[17] Br¨ohl C, Nelles J, Brandl C, Mertens A, Nitsch V (2019) Human–Robot Collab-oration Acceptance Model: Development and Comparison for Germany, Japan, China and the USA. Int J Social Robot 11(5):709–726. 10.1007/s12369-019-00593-0. Accessed 27 Aug 2025

[18] Schneiders E, Papachristos E, Berkel NV, Jacobsen RM (2022) Briefly enter-taining but pointless: perceived benefits & risks of social robots in the home. In: CHI EA ’23 Proceedings of the 2023 CHI Conference on Human Factors in Computing Systems. 10.1145/3544549.3585696

[19] Graaf MMA, Somaya BA, Dijk JAGM (2019) Why would I use this in my home? A model of domestic social robot acceptance. Human–Comput Interact 34(2):115–173. Publisher: Taylor & Francis eprint: 10.1080/07370024.2017.1312406. Accessed 19 Jun 2025

[20] L¨offler D, D¨orrenb¨acher J, Hassenzahl M (2020) The Uncanny Valley Effect in Zoomorphic Robots: The U-Shaped Relation Between Animal Likeness and Likeability. In: Proceedings of the 2020 ACM/IEEE International Conference on Human-Robot Interaction. HRI ’20. Association for Comput-ing Machinery, New York, NY, USA, pp. 261–270. 10.1145/3319502.3374788

[21] Sasser JA, McConnell DS, Smither JA (2024) Investigation of Relation-ships Between Embodiment Perceptions and Perceived Social Presence in Human–Robot Interactions. Int J Social Robot 16(8):1735–1750. 10.1007/s12369-024-01138-w. Accessed 2024-10-01

[22] Rammstedt B, John OP (2007) Measuring personality in one minute or less: A 10-item short version of the big five inventory in english and german. J Res Pers 41(1):203–212

[23] Waytz A, Cacioppo J, Epley N (2010) Who sees human? the stability and importance of individual differences in anthropomorphism. Perspect Psychol Sci 5(3):219–232 10.1177/1745691610369336 PMC4021380 24839457

[24] Zilcha-Mano S, Mikulincer M, Shaver PR (2011) An attachment perspective on human–pet relationships: Conceptualization and assessment of pet attachment orientations. J Res Pers 45(4):345–357

[25] Dzedzickis A, Vaiˇciu¯nas G, Lapkauskaite˙ K, Virˇzonis D, Buˇcinskas V (2024) Recent advances in human–robot interaction: robophobia or synergy. J Intell Manuf. 10.1007/s10845-024-02362-x. Accessed 2024-11-04

[26] Hinz N-A, Ciardo F, Wykowska A (2019) Individual Differences in Attitude Toward Robots Predict Behavior in Human-Robot Interaction. In: Salichs MA, Ge SS, Barakova EI, Cabibihan J-J, Wagner AR, Castro-Gonz´alez A, He H (eds) Social Robotics. Springer, Cham, pp 64–73. 10.1007/978-3-030-35888-47

[27] Matthews G, Lin J, Panganiban AR, Long MD (2020) Individual Differences in Trust in Autonomous Robots: Implications for Transparency. IEEE Trans-actions Human-Machine Syst 50(3):234–244. 10.1109/THMS.2019.2947592. Accessed 2025-06-18

[28] Graaf MMA, Allouch SB (2017) The Influence of Prior Expectations of a Robot’s Lifelikeness on Users’ Intentions to Treat a Zoomorphic Robot as a Companion. Int J Social Robot 9(1):17–32. 10.1007/s12369-016-0340-4. Accessed 2024-04-25

[29] Randall N, Sˇabanovi´c S (2024) Designing robots for marketplace success: a case study with technology for behavior and habit change. Int J Social Robot 16(3):461–487. 10.1007/s12369-023-01093-y. Accessed 2024-12-18

[30] Young JE, Sung J, Voida A, Sharlin E, Igarashi T, Christensen HI, Grinter RE (2011) Evaluating human-robot interaction: Focusing on the holistic interaction experience. Int J Soc Robot 3(1):53–67. Publisher: Kluwer Academic Publishers. 10.1007/s12369-010-0081-8

[31] Grimme S, Kollakidou A, Zarp C, Hornecker E, Kru¨ger N, Graf P, Marchetti E (2023) Floor cleaners as helper pets: projecting assistive robots’ agency on zoomorphic affordances. SN Comput Sci 4. 10.1007/s42979-023-01769-2 PMC10148613 37152861

[32] Sung J-Y, Guo L, Grinter RE, Christensen HI (2007) My roomba is rambo: intimate home appliances. In: Krumm J, Abowd GD, Seneviratne A, Strang T (eds) UbiComp 2007: Ubiquitous Computing. Springer, Berlin, Heidelberg, pp 145–162. 10.1007/978-3-540-74853-39

[33] Craenen B, Deshmukh A, Foster ME, Vinciarelli A (2018) Do we really like robots that match our personality? the case of big-five traits, godspeed scores and robotic gestures. In: 2018 27th IEEE International Symposium on Robot and Human Interactive Communication (RO-MAN), IEEE, pp. 626–631.

[34] Wainer J, Feil-seifer DJ, Shell DA, Mataric MJ (2006) The role of physi-cal embodiment in human-robot interaction. In: ROMAN 2006 - The 15th IEEE International Symposium on Robot and Human Interactive Commu-nication, pp. 117–122 10.1109/ROMAN.2006.314404. ISSN: 1944–9437. https://ieeexplore.ieee.org/abstract/document/4107795. Accessed 2025-06-20

[35] Hoffmann L, Bock N, Pu¨tten AM (2018) The peculiarities of robot embod-iment (emcorp-scale): development, validation and initial test of the embodiment and corporeality of artificial agents scale. In: Proceed-ings of the 2018 ACM/IEEE International Conference on Human-Robot Interaction. HRI ’18, pp. 370–378. Association for Computing Machin-ery, New York, NY, USA 10.1145/3171221.3171242. Accessed 20 Jun 2025

[36] Deng E, Mutlu B, Mataric MJ (2019) Embodiment in Socially interactive robots. Found Trends^®^ Rob 7(4):251–356. Publisher: Now Publishers, Inc. 10.1561/2300000056. Accessed 20 Jun 2025

[37] Pu¨tten AM, Kr¨amer NC (2012) A survey on robot appearances. In: Proceed-ings of the Seventh Annual ACM/IEEE International Conference on Human-Robot Interaction. HRI ’12, Association for Computing Machin-ery, New York, NY, USA, pp. 267–268. 10.1145/2157689.2157787

[38] Bradwell HL, Winnington R, Thill S, Jones RB (2021) Morphology of socially assistive robots for health and social care: A reflection on 24 months of research with anthropomorphic, zoomorphic and mechanomorphic devices. In: 2021 30th IEEE International Conference on Robot and Human Interactive Communication, RO-MAN 2021, pp. 376–383 10.1109/RO-MAN50785.2021.9515446

[39] Mori M, MacDorman KF, Kageki N (2012) The uncanny valley [from the field]. IEEE Rob Autom Magazine 19(2):98–100. 10.1109/MRA.2012.2192811

[40] Saygin AP, Chaminade T, Ishiguro H, Driver J, Frith C (2012) The thing that should not be: predictive coding and the uncanny valley in perceiving human and humanoid robot actions. Soc Cognit Affect Neurosci 7(4):413–422. 10.1093/scan/nsr025. Accessed 2025-06-20 PMC3324571 21515639

[41] Yam KC, Bigman Y, Gray K (2021) Reducing the uncanny valley by dehumanizing humanoid robots. Comput Hum Behav 125:106945. 10.1016/j.chb.2021.106945. Accessed 2025-06-20

[42] Barker S, Dawson K (1998) The effects of animal-assisted therapy on anxiety ratings of hospitalized psychiatric patients. Psychiatric Services 49(6). 10.1176/ps.49.6.797. ISBN: 1075-2730 9634160

[43] Calvert MM (1989) Human-pet interaction and loneliness: a test of concepts from roy’s adaptation model. Nurs Sci Quart 2(4):194–202. Publisher: SAGE Publications Inc. 10.1177/089431848900200409. Accessed 07 Jan 2025 2594276

[44] Kramer SC, Friedmann E, Bernstein PL (2009) Comparison of the effect of human interaction, animal-assisted therapy, and AIBO-assisted therapy on long-term care residents with dementia. Anthrozoos 22(1):43–57. 10.2752/175303708X390464

[45] Iham¨aki P, Heljakka K (2021) Robot pets as serious toys- activating social and emotional experiences of elderly people. Information Systems Frontiers (August). Publisher: Information Sys-tems Frontiers. 10.1007/s10796-021-10175-z PMC8364409 34413702

[46] Shibata T, Kawaguchi Y, Wada K (2009) Investigation on people living with Paro at home effects of sex difference and owners’ animal preference. In: Proceedings - IEEE International Workshop on Robot and Human Interactive Communication. IEEE. pp 1131–1136. 10.1109/ROMAN.2009.5326201. ISBN: 9781424450817

[47] Saunderson S (2022) Why We See Humanity in Everyday Things https://shanesaunderson.com/2022/04/06/why-we-see-humanity-in-everyday-objects/. Accessed 28 Apr 2025

[48] Niess J, Wo´zniak PW (2020) Embracing Companion Technologies. In: Proceed-ings of the 11th Nordic Conference on Human-Computer Interaction: Shaping Experiences, Shaping Society. NordiCHI ’20. Association for Comput-ing Machinery, New York, NY, USA, pp. 1–11. 10.1145/3419249.3420134. Accessed 11 Feb 2025

[49] Osawa H, Mukai J, Imai M (2007) Graduate School of Science and Technology, Keio University, 3-14-1 Hiyoshi, Kohoku-Ku, Yokohama 223–8522, Japan, Faculty of Science and Technology, Keio University, 3-14-1 Hiyoshi, Kohoku-Ku, Yoko-hama 223–8522, Japan: Anthropomorphization Framework for Human-Object Communication. J Adv Comput Intell Intell Inf 11(8):1007–1014. 10.20965/jaciii.2007.p1007. Accessed 28 Apr 2025

[50] Ye H, You R, Lou K, Wen Y, Yi X, Tong X (2024) I Keep Sweet Cats In Real Life, But What I Need In The Virtual World Is A Neurotic Dragon: Virtual Pet Design With Personality Patterns. In: Proceedings of the Eleventh International Symposium of Chinese CHI. CHCHI ’23, Association for Comput-ing Machinery, New York, NY, USA, pp. 210–224. 10.1145/3629606.3629626. Accessed 10 Apr 2025

[51] Lin C, Faas T, Dombrowski L, Brady E (2017) Beyond cute: exploring user types and design opportunities of virtual reality pet games. In: Proceedings of the 23rd ACM Symposium on Virtual Reality Software And Technology. VRST ’17. Association for Computing Machin-ery, New York, NY, USA, pp. 1–10. 10.1145/3139131.3139132. Accessed 10 Feb 2025

[52] Lakhal S, Khechine H (2017) Relating personality (big five) to the core constructs of the unified theory of acceptance and use of technology. J Computers Educ 4(3):251–282

[53] Nomura T, Suzuki T, Kanda T, Kato K (2006) Measurement of negative atti-tudes toward robots. Interact Stud Social Behav Communication Biol Artif Syst 7(3):437–454

[54] Mobed DAO, Wodehouse A, Maier A (2024) The aesthetics of robot design: towards a classification of morphologies. Proc Des Soc 4:2413–2422. 10.1017/pds.2024.244

[55] Forlizzi J (2007) How robotic products become social products: an ethnographic study of cleaning in the home. In: Proceedings of the ACM/IEEE International Conference on Human-robot Interaction. HRI ’07, Association for Computing Machinery, New York, NY, USA. pp. 129–136. 10.1145/1228716.1228734

[56] Forlizzi J, DiSalvo C (2006) Service robots in the domestic environment: a study of the roomba vacuum in the home. In: Proceedings of the 1st ACM SIGCHI/SIGART Conference on Human-robot Interaction. HRI ’06, Association for Computing Machinery, New York, NY, USA. pp. 258–265. 10.1145/1121241.1121286

[57] Ghafurian M, Lakatos G, Dautenhahn K (2022) The zoomorphic miro robot’s affective expression design and perceived appearance. Int J Soc Robotics 14(4):945–962. Publisher: Springer Netherlands. 10.1007/s12369-021-00832-3 PMC8723815 35003385

[58] Marky K, Macdonald S, Abdrabou Y, Khamis M (2023) In the quest to protect users from {Side-Channel} attacks–a {User-Centred} design space to miti-gate thermal attacks on public payment terminals. In: 32nd Usenix Security Symposium (usenix Security 23), pp. 5235–5252

[59] Bartneck C, Kuli´c D, Croft E, Zoghbi S (2009) Measurement instruments for the anthropomorphism, animacy, likeability, perceived intelligence, and perceived safety of robots. Int J Social Robot 1:71–81

[60] Wobbrock JHJ, Leah DG, Findlater (2011) The aligned rank transform for nonparametric factorial analyses using only anova procedures. CHI 2011 1–5

[61] Braun V, Clarke V (2006) Using thematic analysis in psychology. Qual Res Psychol (3):77–101. 10.1017/CBO9781107415324.004. arXiv: 1011.1669v3 ISBN: 9788578110796

[62] Nomura T, Kanda T, Suzuki T, Kato K (2008) Prediction of human behavior in human–robot interaction using psychological scales for anxiety and negative attitudes toward robots. IEEE Trans Robot 24(2):442–451. 10.1109/TRO.2007.914004

[63] Graaf MMA, Ben Allouch S (2013) The relation between people’s attitude and anx-iety towards robots in human-robot interaction. In: 2013 IEEE RO-MAN, pp. 632–637 10.1109/ROMAN.2013.6628419

[64] Braun V, Clarke V (2013) Successful Qualitative Research: A Practical Guide for Beginners. SAGE, London

[65] Weiß M, Baumeister H, Cohrdes C, Deckert J, Gru¨ndahl M, Pryss R, Hein G (2022) Extraversion moderates the relationship between social media use and depression. J Affect Disorders Rep 8:100343

[66] Berner J, Dallora AL, Palm B, Sanmartin Berglund J, Anderberg P (2023) Five-factor model, technology enthusiasm and technology anxiety. Digit Health 9:20552076231203602 10.1177/20552076231203602 PMC10515511 37744749

[67] Chiu C-J, Hsieh S, Li C-W (2021) Needs and preferences of middle-aged and older adults in taiwan for companion robots and pets: survey study. J Med Internet Res 23(6):23471 10.2196/23471 PMC8386361 34347621

[68] Jo H, Park D-H (2024) A multi-faceted examination of social robots adoption: Influ-ences of perceived enjoyment, social attraction, and pet experience. IEEE Access

[69] Chien S-E, Chu L, Lee H-H, Yang C-C, Lin F-H, Yang P-L, Wang T-M, Yeh S-L (2019) Age difference in perceived ease of use, curiosity, and implicit negative attitude toward robots. J Hum -Robot Interact 8(2). 10.1145/3311788

[70] Kert´esz C, Turunen M (2019) Exploratory analysis of sony aibo users. AI Soc 34(3):625–638

[71] Graaf M, Allouch S, Dijk JAGM (2016) Long-term acceptance of social robots in domestic environments: insights from a user’s perspective. In: AAAI Spring symposiaAAAI Spring Symposia

[72] Mandal FB (2014) Nonverbal communication in humans. J Hum Behav social Environ 24(4):417–421

[73] Briones EM, Marshall PH (2022) The perception and effect of aggressive dog breeds on human social interactions. Human-Animal Interaction Bulletin (2022)

[74] Ahmed E, Genc¸ C¸, Spors ., Hamari V, Buruk J (2026) O. Roaming with a robot: Analyzing the experiences and understanding the dimensions of designing human-robot walking interactions. In: Proceedings of the 2026 CHI Conference on Human Factors in Computing Systems, pp. 1–18

[75] Ojha S, Vitale J, Williams M-A (2021) Computational emotion models: a thematic review. Int J Social Robot 13(6):1253–1279

[76] Macdonald S, ElSayed S, McGill M (2025) AZRA: extending the affective capabil-ities of zoomorphic robots using augmented reality. In: Companion of the 2025 ACM/IEEE International Conference on Human-Robot Interaction (RO-MAN 2025)

[77] Wang Y, Lu Y, Yan S, Shen X (2025) If my apple can talk: Exploring the use of everyday objects as personalized ai agents in mixed reality. In: Proceedings of the Extended Abstracts of the CHI Conference on Human Factors in Computing Systems, pp. 1–9

[78] Iwai N, Matulic F (2025) Bringing Everyday Objects to Life in Aug-mented Reality with AI-Powered Talking Characters. In: Proceedings of the Extended Abstracts of the CHI Conference on Human Factors in Com-puting Systems. CHI EA ’2. Association for Computing Machin-ery, New York, NY, USA, pp. 1–7. 10.1145/3706599.3719978. Accessed 27 Apr 2025

[79] Yao X, Xi Y (2025) From assistants to digital beings: Exploring anthropomorphism, humanness perception, and ai anxiety in large-language-model chatbots. Social Sci Comput Rev. 08944393251354976

[80] Peter S, Riemer K, West JD (2025) The benefits and dangers of anthropomorphic conversational agents. Proc Natl Acad Sci 122(22):2415898122 10.1073/pnas.2415898122 PMC12146756 40378006

[81] Kanat-Maymon Y, Antebi A, Zilcha-Mano S (2016) Basic psychological need fulfillment in human–pet relationships and well-being. Personality Indi-vidual Differences 92:69–73. 10.1016/j.paid.2015.12.025. Accessed 07 Jan 2025

[82] Bork A, Kr¨oger C, Zˇemberi I, Hurrelbrink L, Madam Sampangiramu S, Litvin Y, Ringe R, D¨anekas B, Malaka R (2025) Doggoroomie-examining zoomor-phic robot interactions for promoting active behavior in a comfortable setting. In: Mensch und Computer 2025-Workshopband, Gesellschaft fu¨r Informatik eV. pp. 10–18420

[83] Alves-Oliveira P, Bavier M, Malandkar S, Eldridge R, Sayigh J, Bj¨orling EA, Cakmak M (2022) FLEXI: A Robust and Flexible Social Robot Embodi-ment Kit. In: Proceedings of the 2022 ACM Designing Interactive Systems Conference. DIS ’22. Association for Computing Machin-ery, New York, NY, USA, pp. 1177–1191. 10.1145/3532106.3533541. 10 Jan Accessed 2025

[84] Ahmad MI, Mubin O, Orlando J (2017) A systematic review of adaptivity in human-robot interaction. Multimodal Technologies and Interaction 1(3). Publisher: MDPI AG. 10.3390/mti1030014

[85] Joshi S, Kamino W, Sˇabanovi´c S (2024) Social robot accessories for tailoring and appropriation of social robots. Int J Social Robot. 10.1007/s12369-023-01077-y. Accessed 2025-01-10

[86] ElSayed S, King D (2017) Affect and believability in game characters - A review of the use of affective computing in games. In: 18th International Conference on Intelligent Games and Simulation, GAME-ON 2017, pp. 90–97

[87] Kruger J, Wirtz D, Van Boven L, Altermatt TW (2004) The effort heuristic. J Exp Soc Psychol 40(1):91–98. 10.1016/S0022-1031(03)00065-9. Accessed 25 Mar 2025

[88] Kamino W, Sˇabanovi´c S, Jung MF (2024) The lifecycle of social robots: Obsoles-cence and values in repair. In: 2024 33rd IEEE International Conference on Robot and Human Interactive Communication (ROMAN), IEEE. pp. 2147–2154

[89] Cavallo F, Semeraro F, Fiorini L, Magyar G, Sinˇc´ak P, Dario P (2018) Emo-tion modelling for social robotics applications: a review. J Bionic Eng 15(2):185–203. 10.1007/s42235-018-0015-y

[90] Emmerich K, Ring P, Masuch M (2018) I’m glad you are on my side: How to design compelling game companions. CHI PLAY 2018 - Proceedings of the 2018 Annual Symposium on Computer-Human Interaction in Play. pp 153–162. 10.1145/3242671.3242709. ISBN: 9781450356244

[91] Macdonald S, Bretin R, ElSayed S (2024) Evaluating transferable emotion expres-sions for zoomorphic social robots using vr prototyping. In: 2024 IEEE Inter-national Symposium on Mixed and Augmented Reality (ISMAR), pp. 1087–1096 10.1109/ISMAR62088.2024.00125. ISSN: 2473 – 0726. https://ieeexplore.ieee.org/abstract/document/10765384. Accessed 07 Dec 2024

[92] Groechel T, Shi Z, Pakkar R, Mataric MJ. (2019) Using Socially Expressive Mixed Reality Arms for Enhancing Low-Expressivity Robots. In: 28th IEEE Inter-national Conference on Robot and, Communication RO-MAN 2019, pp. 1–8. IEEE, ???. 10.1109/RO-MAN46459.2019.8956458. arXiv: 1911.09713

[93] Suzuki R, Karim A, Xia T, Hedayati H, Marquardt N (2022) Augmented Real-ity and Robotics: A Survey and Taxonomy for AR-enhanced Human-Robot Interaction and Robotic Interfaces. In: Conference on Human Factors in Com-puting Systems - Proceedings. Association for Computing Machinery, ??? . arXiv: 2203.03254. 10.1145/3491102.3517719

[94] Haring KS, Silvera-Tawil D, Takahashi T, Velonaki M, Watanabe K (2015) Perception of a humanoid robot: a cross-cultural comparison. In: 2015 24th IEEE International Symposium on Robot and Human Interactive Communication (RO-MAN), IEEE. pp. 821–826.

[95] Haring KS, Mougenot C, Ono F, Watanabe K (2014) Cultural Differences in Perception and Attitude towards Robots. Int J Affect Eng 13(3):149–157. 10.5057/ijae.13.149

[96] Nomura T, Horii S (2020) Influences of media literacy and experiences of robots into negative attitudes toward robots in japan. In: 2020 29th IEEE International Conference on Robot and Human Interactive Communication (RO-MAN), IEEE. pp. 286–290

