# Supplementary material for: “Cute Companion” or “Awful Thing”: Understanding Individual Differences in Attitudes to Social Robots as Pets
Source: Int J Soc Robot. 2026 Nov 2;18(8):115. doi: 10.1007/s12369-026-01453-4 (PMC13727923; doi:10.1007/s12369-026-01453-4)
Supplement: Supplementary file 1 [file 12369_2026_1453_MOESM1_ESM.pdf]

Information Sheet

Participant Information

You are being invited to take part in a research study. Before you decide it is important for you to understand why the research is being done and what it will involve. Please take time to read the following information carefully and discuss it with others if you wish. Ask us if there is anything that is not clear or if you would like more information. Take time to decide whether or not you wish to take part.

Purpose of the study:

The purpose of this study is to investigate the factors which lead individuals to be more or less receptive to pets, robots and robots that serve as pets or household companions. To this end we will ask you about your demographic and cultural background, ask you questions designed to assess specific ways in which you currently perceive the world around you, pets and robots. The results of this study's investigation will be used to inform the design of future robots which could serve as companions for those who may be unable or

unwilling to keep pets for various practical or personal reasons, but who might still wish for, or benefit from, pet like interaction and companionship.

Do I have to take part?

The participation in the study is voluntary. You may decide whether or not to take part. If you do decide to take part, you will be asked to fill out an online consent form. If you decide to take part, you are still free to withdraw at any time and without giving a reason, in which case any answers you gave will be deleted.

What will happen to me if I take part?

You will be tasked with answering a survey with four main sections. The first section will ask you about your demographics, how you view robots, pets, yourself and the world around you.

The second section will ask you about your emotion and life experiences with pets.

The third section will ask you to watch a short video about five different robots. After each video you will be asked about your perceptions of the robot and about how receptive you would be to that robot as a household companion or pet.

The fourth section will ask you to reflect on your willingness to consider robots as pets or household companions.

Once you complete the fourth section the survey is completed and will have the opportunity to submit your email for future participation or contact the researcher to ask more questions.

What are the possible disadvantages and risks of taking part?

This survey will take approximately 20 minutes of your time to complete.

What are the possible benefits of taking part?

By completing this survey, you will be given the opportunity to enter a contact email and, if so, you will be entered into a prize draw for an Amazon voucher worth £50 or equivalent value. Once the survey closes, one random respondent will receive this voucher. You will also have the opportunity to find out more about this study and the research it is helping to inform.

Will my taking part in this study be kept confidential?

All information which collected, or responses provided, during the survey will be kept strictly confidential. You will be identified by an ID number which in no way will be able to be connected to you. Please note that assurances on confidentiality will be strictly adhered to unless evidence of serious harm, or risk of serious harm, is uncovered. In such cases the University may be obliged to contact relevant statutory bodies/agencies.

What will happen to the results of the research study?

The results will be held and owned by the researcher and the ANONYMISED. The results can be outlined to the participants whenever they become available. The participants won't be identified in any report or publication.

Who is organising and funding the research?
This research is funded by the ANONYMISED.

Who has reviewed the study?

The project has been approved by the ANONYMISED.

Contact for Further Information:

You can contact the researcher of this study ANONYMISED via the following methods:

Email: ANONYMISED

Thank you for volunteering to take part in this study.

Consent Form

Please read the following statements and indicate at the end if you agree to them. If you do not agree, your participation in the study will end.

- I confirm that I have read and understood the Participant Information page.

- I understand that my participation is entirely anonymous and that no personal information will be collected from me.

- I confirm that I am at least 18 years old.

- I understand that my ratings and written thoughts will be recorded and anonymised for analysis.

- I understand that if am a student, no course credits will be awarded for completing this survey.

- I understand that my participation is voluntary and that I am free to withdraw at any time before completing the survey. If I withdraw, all of my data will be deleted.

- I understand that the anonymised research data will be archived in a completely anonymous way in an online database and/or data repository. It may also be published as supplementary material to a scientific article and may be accessed by other researchers as well as by the general public, in line with current data sharing practices.

- I understand that upon completing the survey I will be given the opportunity to provide an email

address be entered in prize draw for a £50 Amazon voucher distributed once the survey closes.

Please select whether you acknowledge and agree to the prior statements.

- I agree with all statements
- I disagree with one or more statements

Demographic Information

How old are you?

- Under 18
- 18-24 years old
- 25-34 years old
- 35-44 years old
- 45-54 years old
- 55-64 years old
- 65+ years old

How do you describe yourself?

- Male
- Female
- Non-binary / third gender
- [ ] Prefer to self-describe
- Prefer not to say

What is the highest level of education you have completed?

- Some Primary
- Completed Primary School
- Some Secondary
- Completed Secondary School
- Vocational or Similar
- Some University but no degree
- University Bachelors Degree
- Graduate or professional degree (MA, MS, MBA, PhD, JD, MD, DDS)
- Prefer not to say

How many children under 18 live with you?

What best describes your employment status over the last three months?

- Working full-time
- Working part-time
- Unemployed and looking for work
- A homemaker or stay-at-home parent
- Student
- Retired
- Other

What best describes your cultural background?

Do you currently live with one or more pets, or have you lived with pets in the past?

- Yes
- No

What Kind of Pet

If so, what kind of pets are/were they?

Reptile	Fish	Invertebrate	Other	Bird	Cat	Dog	Rodent
☐	☐	☐	☐	☐	☐	☐	☐

BFI-10

How well do the following statements describe your personality?

	Disagree Strongly	Disagree a little	Neither agree nor disagree	Agree a little	Agree strongly
I see myself as someone who is reserved.	○	○	○	○	○
I see myself as someone who is generally trusting.	○	○	○	○	○
I see myself as someone who tends to be lazy.	○	○	○	○	○
I see myself as someone who is relaxed, handles stress well.	○	○	○	○	○
I see myself as someone who has few artistic interests.	○	○	○	○	○
I see myself as someone who is outgoing, sociable.	○	○	○	○	○
I see myself as someone who tends to find fault with others.	○	○	○	○	○
I see myself as someone who does a thorough job.	○	○	○	○	○
I see myself as someone who gets	○	○	○	○	○

nervous easily.

I see myself as someone who has an active imagination. ○ ○ ○ ○ ○

NARS

Please indicate your agreement with the following statements.

	Strongly disagree	Somewhat disagree	Neither agree nor disagree	Somewhat agree	Strongly agree
I would feel uneasy if robots really had emotions.	○	○	○	○	○
Something bad might happen if robots developed into living beings.	○	○	○	○	○
I would feel relaxed talking to robots.	○	○	○	○	○
I would feel uneasy if I was given a job where I had to use robots.	○	○	○	○	○
If robots had emotions, I would be able to make friends with them.	○	○	○	○	○
I feel comforted being with robots that have emotions.	○	○	○	○	○
The word "robot" means nothing to me.	○	○	○	○	○
Please select 'Strongly Agree' to show you are paying attention to this question.	○	○	○	○	○
I would feel nervous					

operating a robot in front of other people.	○	○	○	○	○
I would hate the idea that robots or artificial intelligences were making judgments about things.	○	○	○	○	○
I would feel very nervous just standing in front of a robot.	○	○	○	○	○
I feel that if I depend on robots too much, something bad might happen.	○	○	○	○	○
I would feel paranoid talking with a robot.	○	○	○	○	○
I am concerned that robots would be a bad influence on children.	○	○	○	○	○
I feel that in the future society will be dominated by robots.	○	○	○	○	○

IDAQ

Next, we will ask you to rate the extent to which you believe various stimuli (e.g. technological or mechanical items, wild and domestic animals, and natural things) possess certain capacities. On a 0-10 scale (where 0 = "Not at All" and 10 = "Very much"), please rate the extent to which the stimulus possesses the capacity given.

By "has a mind of its own" we mean able to do what it wants.
By "has free will" we mean able to choose and control its own actions.
By "has intentions" we mean has preferences and plans.
By "can experience emotion" we mean it has feelings.
By "has consciousness" we mean able to be aware of itself and its thoughts and feelings.

	0 - Not at all	1	2	3	4	5	6	7	8	9	10 - Very Much
To what extent does technology—devices and machines for manufacturing, entertainment, and productive processes (e.g. cars, computers, television sets)—have intentions.	○	○	○	○	○	○	○	○	○	○	○
To what extent											

does the average fish have free will.	○	○	○	○	○	○	○	○	○	○	○
To what extent does the average mountain have free will?	○	○	○	○	○	○	○	○	○	○	○
To what extent does a television set experience emotions?	○	○	○	○	○	○	○	○	○	○	○
To what extent does the average robot have consciousness.	○	○	○	○	○	○	○	○	○	○	○
To what extent do cows have intentions?	○	○	○	○	○	○	○	○	○	○	○
To what extent does a car have free will?	○	○	○	○	○	○	○	○	○	○	○
To what extent does the ocean have consciousness?	○	○	○	○	○	○	○	○	○	○	○
To what extent does the average computer have a mind of its own.	○	○	○	○	○	○	○	○	○	○	○
Please select '0 - Not at all' to show you are paying attention to this question.	○	○	○	○	○	○	○	○	○	○	○
To what extent does a cheetah experience emotions?	○	○	○	○	○	○	○	○	○	○	○

To what extent does the environment experience emotions?	○	○	○	○	○	○	○	○	○	○	○
To what extent does the average insect have a mind of its own?	○	○	○	○	○	○	○	○	○	○	○
To what extent does a tree have a mind of its own?	○	○	○	○	○	○	○	○	○	○	○
To what extent does the wind have intentions?	○	○	○	○	○	○	○	○	○	○	○
To what extent does the average reptile have consciousness?	○	○	○	○	○	○	○	○	○	○	○

PAQ and Pet Experiences

We are interested in your experiences or relationship with a specific pet. If you have more than one pet, please select the one that you have or had the closest relationship with.

Respond to each statement by indicating how much you agree or disagree with it, using the following rating scale 1 - 7, where 1 = Disagree Strongly, 4= Neutral/ Mixed and 7 = Agree Strongly.

	1 - Disagree Strongly	2	3	4 Neutral/ Mixed	5	6	7 - Agree Strongly
Being close to my pet is pleasant for me	○	○	○	○	○	○	○
I'm often worried about what I'll do if something bad happens to my pet	○	○	○	○	○	○	○
I prefer not to be too close to my pet	○	○	○	○	○	○	○
Sometimes I feel that I force my pet to show more commitment and desire to be close to me	○	○	○	○	○	○	○
I prefer to keep some distance from my pet	○	○	○	○	○	○	○
If I can't get my pet to show interest in me, I get upset or	○	○	○	○	○	○	○

me, I get upset or angry

Often my pet is a nuisance to me	○	○	○	○	○	○	○
Signs of affection from my pet bolster my self-worth	○	○	○	○	○	○	○
I feel distant from my pet	○	○	○	○	○	○	○
I often feel that my pet doesn't allow me to get as close as I would like	○	○	○	○	○	○	○
I'm not very attached to my pet	○	○	○	○	○	○	○
I get angry when my pet doesn't want to be close to me as much as I would like to	○	○	○	○	○	○	○
If necessary, I would be able to give away my pet without any difficulties	○	○	○	○	○	○	○
I get frustrated when my pet is not around as much as I would like it to be	○	○	○	○	○	○	○
I have no problem parting with my pet for a long duration	○	○	○	○	○	○	○
I need shows of affection from my pet to feel there is	○	○	○	○	○	○	○

- someone who accepts me as I am
- I get uncomfortable when my pet wants to be close to me
- I feel frustrated if my pet doesn't seem to be available for me when I need it
- I get nervous when my pet gets too close to me
- Without acts of affection from my pet I feel worthless
- I want to get close to my pet, but I keep pulling away
- I am worried about being left alone without my pet
- I try to avoid getting too close to my pet
- I need expressions of love from my pet to feel valuable
- When I'm away from my pet for a long period of time, I hardly think about it.
- I need a lot of

reassurance from my pet that it loves me ○ ○ ○ ○ ○ ○ ○

Considering the relationship you have or had with this pet, please summarise experiences that you feel are or were important or beneficial for you.

How emotionally close do you or did you they feel with this pet? Do/did any specific experiences or interactions feel especially emotionally close or intimate?

Robot Receptivity - NAO

NAO is a humanoid robot which can see, speak, listen, move and dance.

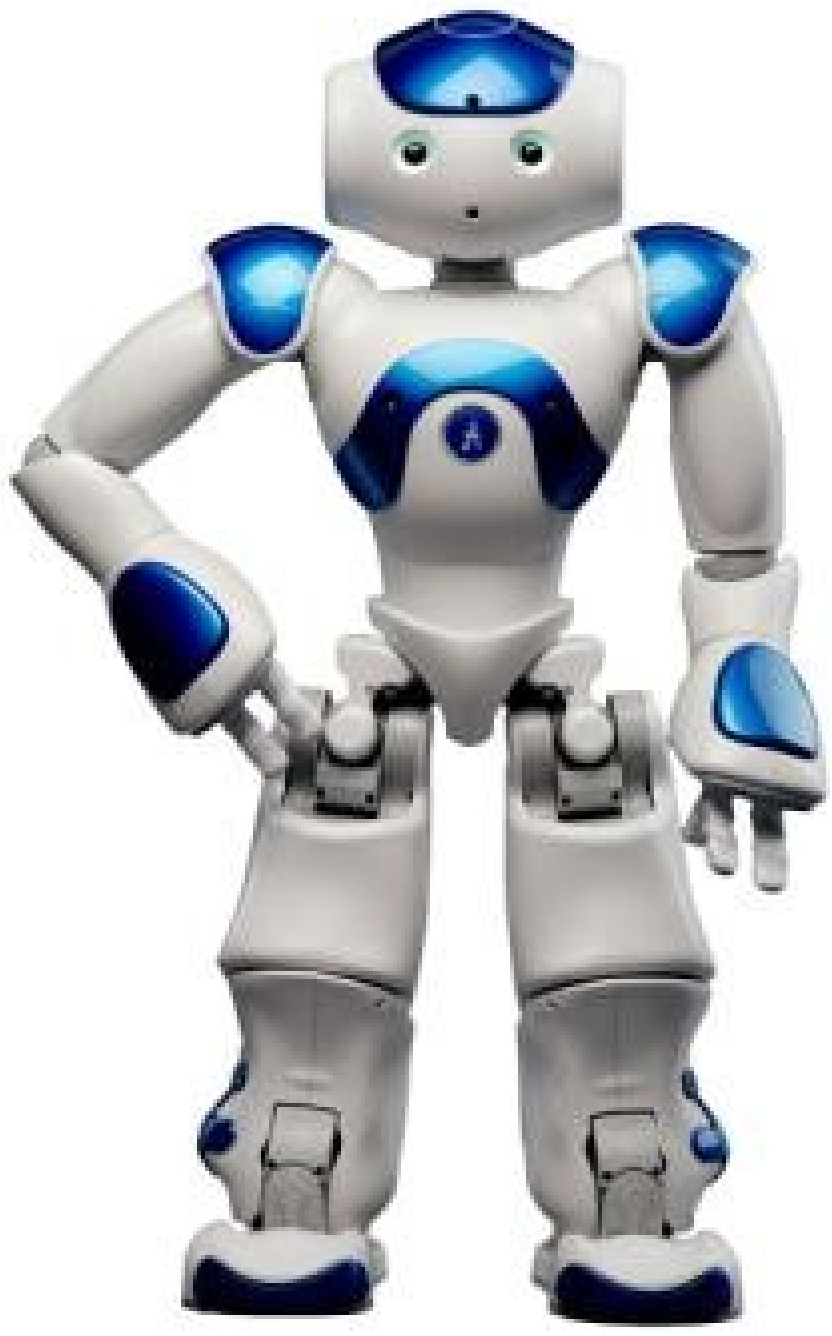

Watch the video to see NAO's features.

https://www.youtube.com/watch?v=HKFHUrx23ts&t=8s

Please rate your impression of the robot on these scales:

1 - Dead	2	3	4	5 - Alive
1 - Stagnant	2	3	4	5 - Lively
1 - Mechanical	2	3	4	5 - Organic
1 - Artificial	2	3	4	5 - Lifelike
1 - Inert	2	3	4	5 - Interactive

1 - Apathetic	2	3	4	5 - Responsive
○	○	○	○	○

1 - Dislike	2	3	4	5 - Like
○	○	○	○	○

1 - Unfriendly	2	3	4	5 - Friendly
○	○	○	○	○

1 - Unkind	2	3	4	5 - Kind
○	○	○	○	○

1 - Unpleasant	2	3	4	5 - Pleasant
○	○	○	○	○

1 - Awful	2	3	4	5 - Nice
○	○	○	○	○

1 - Incompetent	2	3	4	5 - Competent
○	○	○	○	○

1 - Ignorant	2	3	4	5 - Knowledgeable
○	○	○	○	○

1 - Irresponsible	2	3	4	5 - Responsible
○	○	○	○	○

1 - Unintelligent	2	3	4	5 - Intelligent
○	○	○	○	○

1 - Foolish	2	3	4	5 - Sensible
○	○	○	○	○

Rate how receptive you are to this robot as a potential household companion from 1 - 7.

1 - Not very receptive	2	3	4 - Neutral / Unsure	5	6	7 - Very Receptive
○	○	○	○	○	○	○

Why did you give this rating?

Robot Receptivity - Paro

Paro is a robot modelled after a baby Harp Seal which moves, responds to touch, blinks and makes sound.

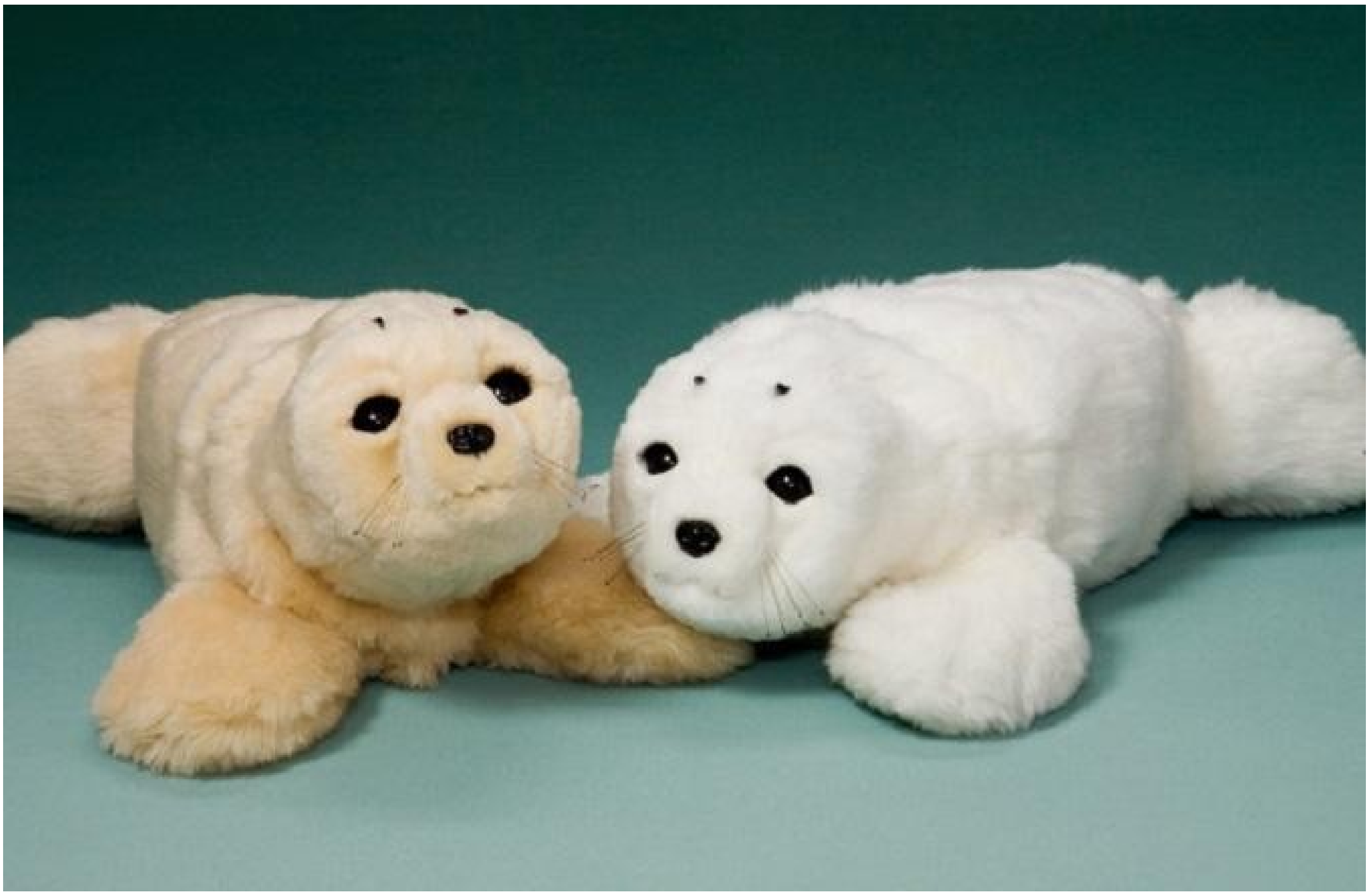

Watch the video to see Paro's features.

https://www.youtube.com/watch?v=b1HbBqZrpe8

Please rate your impression of the robot on these scales:

1 - Dead	2	3	4	5 - Alive
1 - Stagnant	2	3	4	5 - Lively
1 - Mechanical	2	3	4	5 - Organic
1 - Artificial	2	3	4	5 - Lifelike

1 - Inert	2	3	4	5 - Interactive
○	○	○	○	○

1 - Apathetic	2	3	4	5 - Responsive
○	○	○	○	○

1 - Dislike	2	3	4	5 - Like
○	○	○	○	○

1 - Unfriendly	2	3	4	5 - Friendly
○	○	○	○	○

1 - Unkind	2	3	4	5 - Kind
○	○	○	○	○

1 - Unpleasant	2	3	4	5 - Pleasant
○	○	○	○	○

1 - Awful	2	3	4	5 - Nice
○	○	○	○	○

1 - Incompetent	2	3	4	5 - Competent
○	○	○	○	○

1 - Ignorant	2	3	4	5 - Knowledgeable
○	○	○	○	○

1 - Irresponsible	2	3	4	5 - Responsible
○	○	○	○	○

1 - Unintelligent	2	3	4	5 - Intelligent
○	○	○	○	○

1 - Foolish	2	3	4	5 - Sensible
○	○	○	○	○

Rate how receptive you are to this robot as a potential household companion from 1 - 7.

1 - Not very receptive	2	3	4 - Neutral / Unsure	5	6	7 - Very Receptive
○	○	○	○	○	○	○

Why did you give this rating?

____

Robot Receptivity - Qoobo

Petit Qoobo is an abstract zoomorphic robot with an

expressive moving tail and a soft heartbeat.

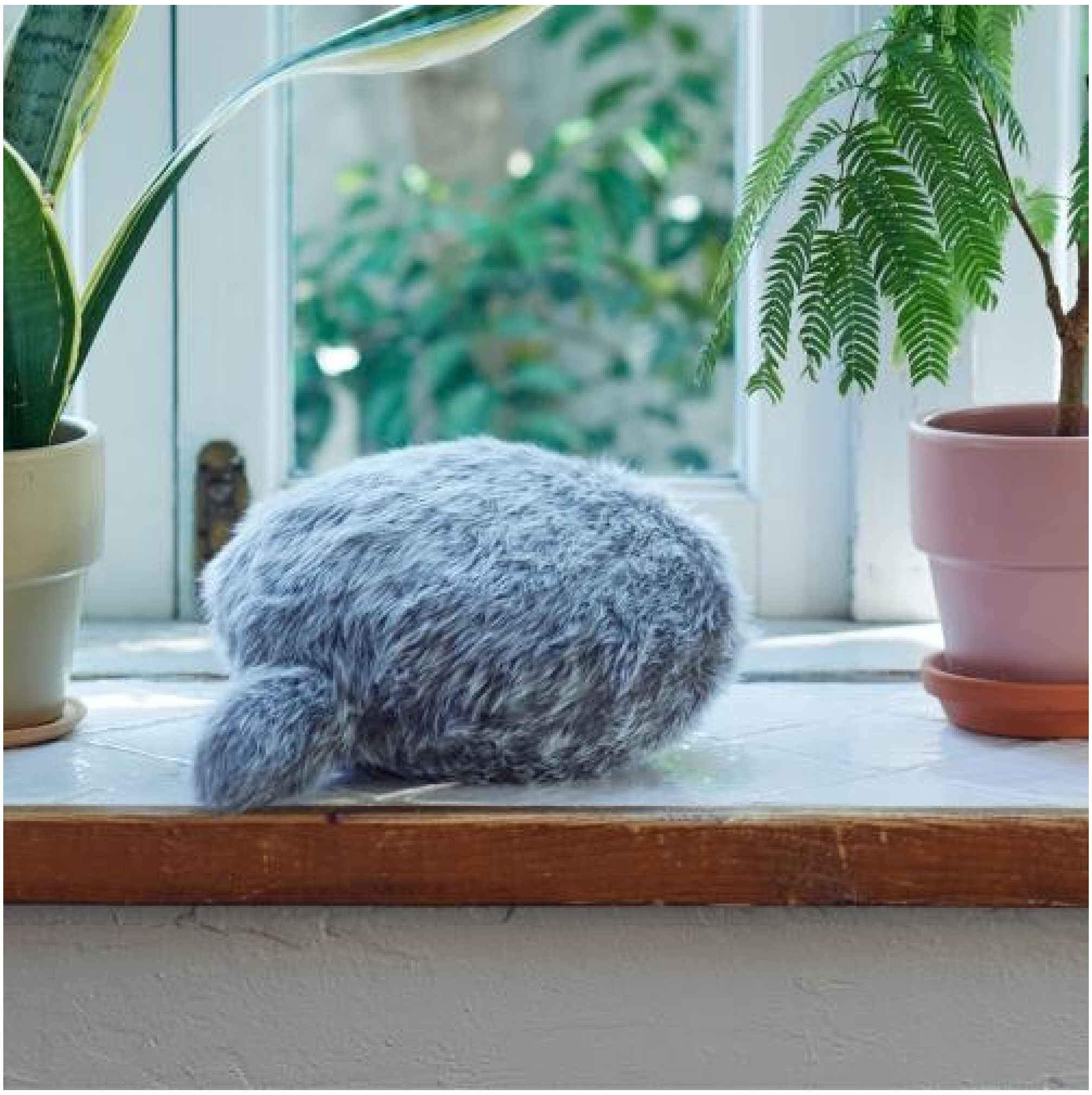

Watch the video to see Petit Qoobo's features.

https://www.youtube.com/watch?v=YxNMR7wM-ZI

Please rate your impression of the robot on these scales:

1 - Dead	2	3	4	5 - Alive
1 - Stagnant	2	3	4	5 - Lively
1 - Mechanical	2	3	4	5 - Organic
1 - Artificial	2	3	4	5 - Lifelike

1 - Inert	2	3	4	5 - Interactive
○	○	○	○	○

1 - Apathetic	2	3	4	5 - Responsive
○	○	○	○	○

1 - Dislike	2	3	4	5 - Like
○	○	○	○	○

1 - Unfriendly	2	3	4	5 - Friendly
○	○	○	○	○

1 - Unkind	2	3	4	5 - Kind
○	○	○	○	○

1 - Unpleasant	2	3	4	5 - Pleasant
○	○	○	○	○

1 - Awful	2	3	4	5 - Nice
○	○	○	○	○

1 - Incompetent	2	3	4	5 - Competent
○	○	○	○	○

1 - Ignorant	2	3	4	5 - Knowledgeable
○	○	○	○	○

1 - Irresponsible	2	3	4	5 - Responsible
○	○	○	○	○

1 - Unintelligent	2	3	4	5 - Intelligent
○	○	○	○	○

1 - Foolish	2	3	4	5 - Sensible
○	○	○	○	○

Rate how receptive you are to this robot as a potential household companion from 1 - 7.

1 - Not very receptive	2	3	4 - Neutral / Unsure	5	6	7 - Very Receptive
○	○	○	○	○	○	○

Why did you give this rating?

[ ]

Robot Receptivity - Cosmo

Cozmo is a non-zoomorphic robot pet with expressive

facial expressions, movement and the ability to play simple games.

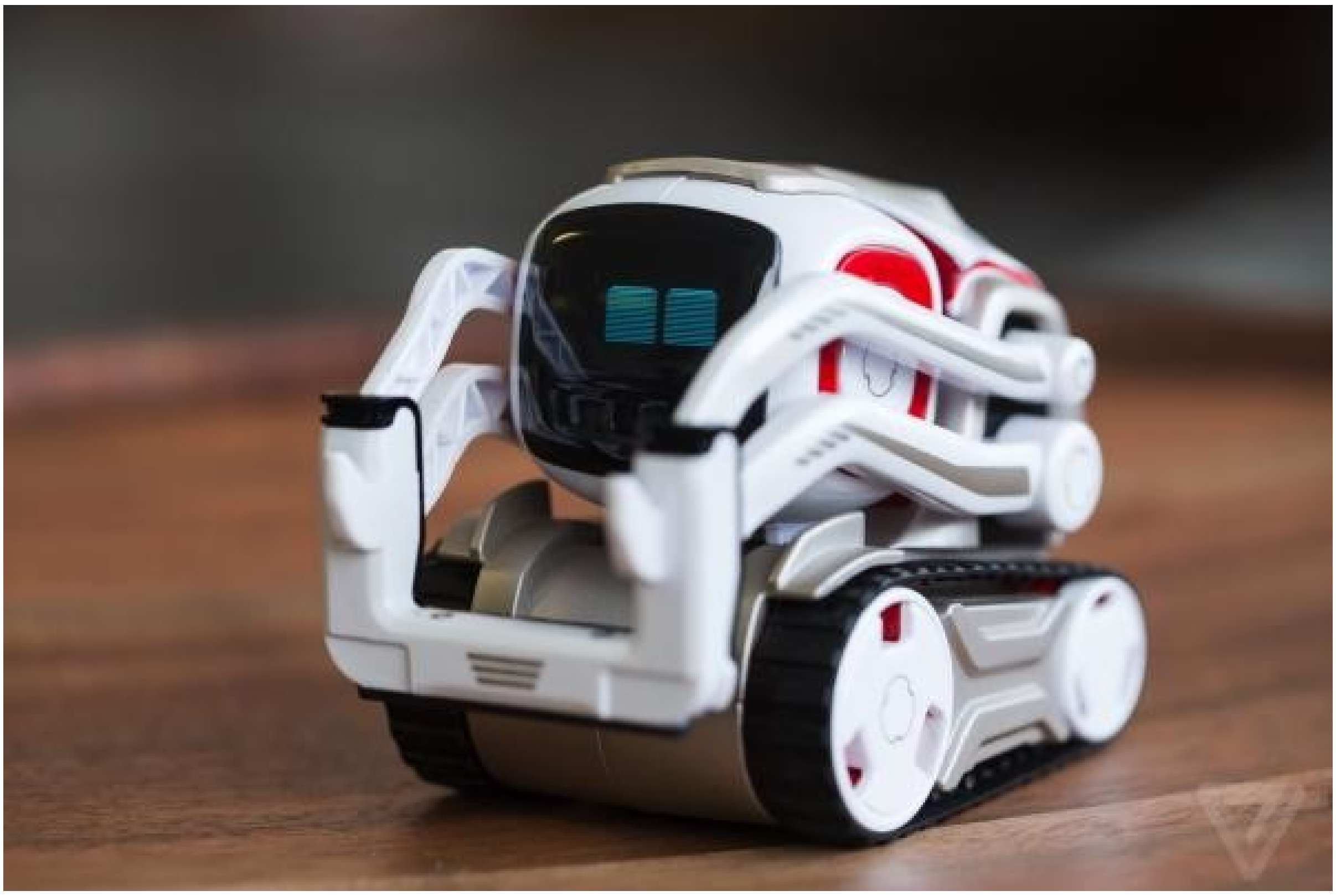

Watch the video to see Cozmo's features.

https://www.youtube.com/watch?v=BWymDFX8PlY

Please rate your impression of the robot on these scales:

1 - Dead	2	3	4	5 - Alive
1 - Stagnant	2	3	4	5 - Lively
1 - Mechanical	2	3	4	5 - Organic
1 - Artificial	2	3	4	5 - Lifelike

1 - Inert	2	3	4	5 - Interactive
○	○	○	○	○

1 - Apathetic	2	3	4	5 - Responsive
○	○	○	○	○

1 - Dislike	2	3	4	5 - Like
○	○	○	○	○

1 - Unfriendly	2	3	4	5 - Friendly
○	○	○	○	○

1 - Unkind	2	3	4	5 - Kind
○	○	○	○	○

1 - Unpleasant	2	3	4	5 - Pleasant
○	○	○	○	○

1 - Awful	2	3	4	5 - Nice
○	○	○	○	○

1 - Incompetent	2	3	4	5 - Competent
○	○	○	○	○

1 - Ignorant	2	3	4	5 - Knowledgeable
○	○	○	○	○

1 - Irresponsible	2	3	4	5 - Responsible
○	○	○	○	○

1 - Unintelligent	2	3	4	5 - Intelligent
○	○	○	○	○

1 - Foolish	2	3	4	5 - Sensible
○	○	○	○	○

Rate how receptive you are to this robot as a potential household companion from 1 - 7.

1 - Not very receptive	2	3	4 - Neutral / Unsure	5	6	7 - Very Receptive
○	○	○	○	○	○	○

Why did you give this rating?

Robot Receptivity - Roomba

Roomba is a functional cleaning robot which can move

around the home and vacuum automatically.

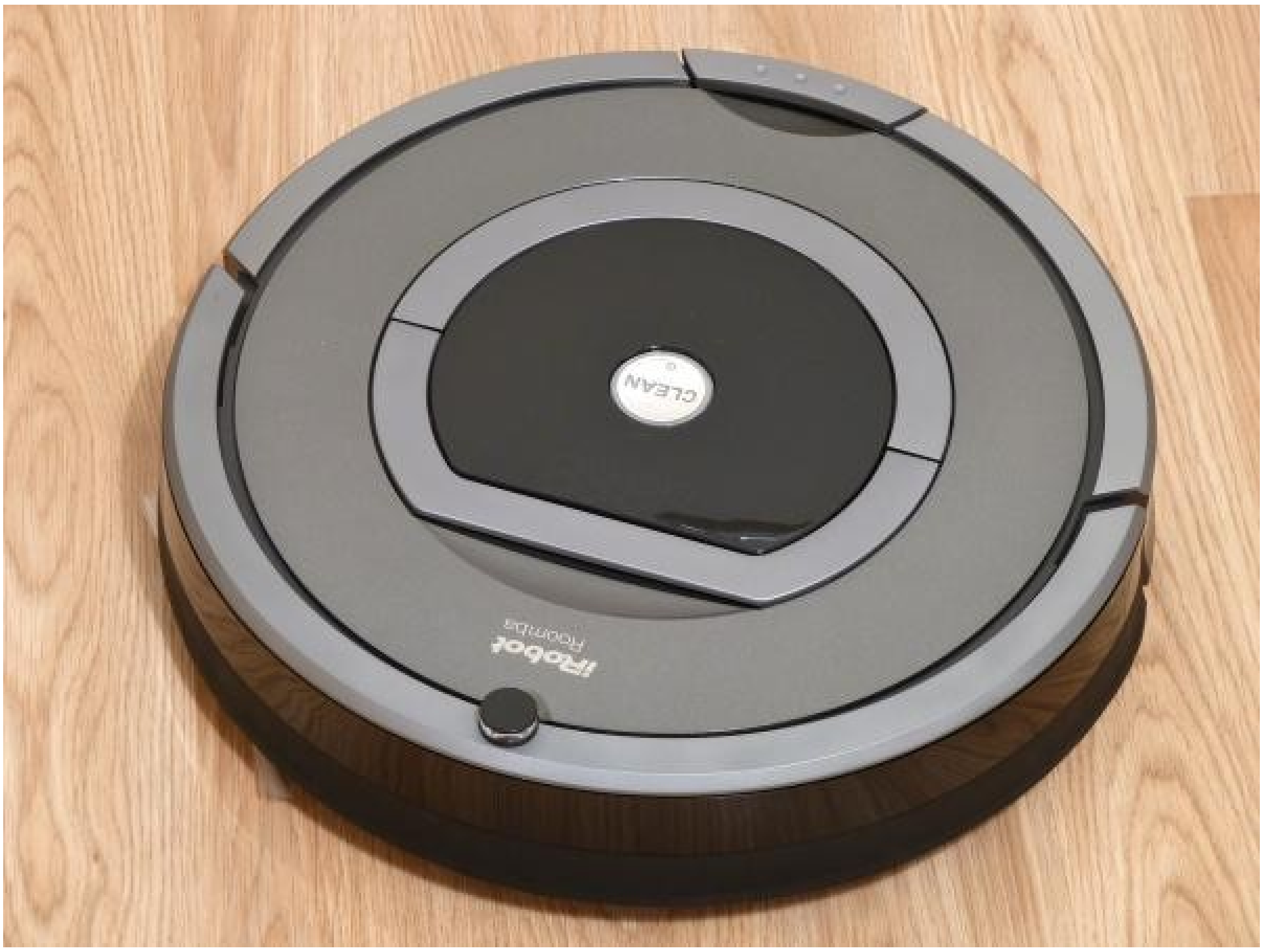

Watch the video to see Roomba's features.

https://www.youtube.com/watch?v=sbr1hfgwJ0A

Please rate your impression of the robot on these

scales:

1 - Dead	2	3	4	5 - Alive
1 - Stagnant	2	3	4	5 - Lively
1 - Mechanical	2	3	4	5 - Organic
1 - Artificial	2	3	4	5 - Lifelike

1 - Inert	2	3	4	5 - Interactive
○	○	○	○	○

1 - Apathetic	2	3	4	5 - Responsive
○	○	○	○	○

1 - Dislike	2	3	4	5 - Like
○	○	○	○	○

1 - Unfriendly	2	3	4	5 - Friendly
○	○	○	○	○

1 - Unkind	2	3	4	5 - Kind
○	○	○	○	○

1 - Unpleasant	2	3	4	5 - Pleasant
○	○	○	○	○

1 - Awful	2	3	4	5 - Nice
○	○	○	○	○

1 - Incompetent	2	3	4	5 - Competent
○	○	○	○	○

1 - Ignorant	2	3	4	5 - Knowledgeable
○	○	○	○	○

1 - Irresponsible	2	3	4	5 - Responsible
○	○	○	○	○

1 - Unintelligent	2	3	4	5 - Intelligent
○	○	○	○	○

1 - Foolish	2	3	4	5 - Sensible
○	○	○	○	○

Rate how receptive you are to this robot as a potential household companion from 1 - 7.

1 - Not very receptive	2	3	4 - Neutral / Unsure	5	6	7 - Very Receptive
○	○	○	○	○	○	○

Why did you give this rating?

Future Speculation

Do you think there are positive pet-like experiences which you could share with a robot?

- Definitely not
- Probably not
- Unsure
- Probably yes
- Definitely yes

If so, what kind of robot, and what of positive pet-like experiences do you think you could share?

What do you perceive as the key differences between a real pet and robot pet?

Can you think of any future technological advancement that could make robots more suitable as household pets?

Prize Draw

Thank you for completing the study! If you wish to entered into the prize draw for a £50 Amazon Voucher, please enter a valid email address below:

Powered by Qualtrics
