# Supplementary material for: “Cute Companion” or “Awful Thing”: Understanding Individual Differences in Attitudes to Social Robots as Pets
Source: Int J Soc Robot. 2026 Nov 2;18(8):115. doi: 10.1007/s12369-026-01453-4 (PMC13727923; doi:10.1007/s12369-026-01453-4)
Supplement: Supplementary file 2 [file 12369_2026_1453_MOESM2_ESM.pdf]

Appendix A Qualitative Codebooks

Receptivity toward Robots Codebook			
Category	*Sub-Category*	*Code*	*Count*
Useful			335
2-4	Are they useful?		185
		Concerns - Don't want/need the companion	20
		Practicalities - Lacks Use/Purpose	56
		Practicalities - Useful/Serves a Practical Purpose	109
2-4	What are they useful for?		150
		Needs - Robot Pet Interaction	13
		Upsides - help with loneliness/social presence	14
		Upsides - Comforting/When I'm Stressed	16
		Concerns - For specific users (Kids/Elderly)	67
		Concerns - More like Object/Ornament/Tool	40
Robots as Pets			508
2-4	Impressions		210
		Positive emotional association/response	29
		Negative emotional association/response	37
		Not sure/not enough info	26
		Upsides - Viable companion	47
		Concerns - Not viable companion	71
2-4	Inherent Differences Between Robot and Pet		176
		Not real vs real	55
		Upsides - Practicality / more Accessible than a pet	18
		Concerns - Robots unemotional/unaffectionate/uncaring, not like pets	34
		Concerns - Lacks Agency/Life	29
		Concerns - Emotionally Manipulative/Exploitative/Clingy	7
		Concerns - Pet Relationships More Meaningful/Payoff	16
		Concerns - One way relationship	4
		Concerns - Real pets are more unpredictable/complex	13
2-4	Existing Pet-Like Benefits		122
		Upsides - Robot is responsive/expressive/interaction	60
		Upsides - Robot has Agency/Life	20
		Upsides - Comfortable with/sharing space with robot	17
		Upsides - Family Friendly	4
		Upsides - Evoked Real Pet Experience	21
Practicalities			69
		Concerns - Privacy / Misuse	11
		Concerns - Cost/Expensive	6
		Concerns - Stigmatised Use	2
		Practicalities - Questionable Reliability/Functionality	37
		Practicalities - Not Early Adopter/Tech Discomfort	10
		Practicalities - Live Service/Tech Support Anxieties	1
		Practicalities - Hard to maintain	2
Assigned Robot Attributes			547
	Associations		192
		Attributes - Is Pet-like	15
		Attributes - Not pet-like	29
		Attributes - Is/too Toy-like	86
		Attributes - Is Plushie-like	12
		Upsides - Design/Tech is Interesting/Advanced	50
	Form Factor		112
		Attributes - Is/too human-like	20
		Attributes - Is/too Mechanical-like	43
		Attributes - Is Animal-like	9
		Attributes - Size	8
		Upsides - Abstract / Simple / Not Too Complex	16
		Concerns - Dislikes Zoomorphic Robots	3
		Concerns - Annoying Sound	13
	Affective Impressions		243
		Attributes - Cute	80
		Attributes - Not Cute	5
		Attributes - Appealing	25
		Attributes - Not Appealing	6
		Attributes - Friendly	12
		Attributes - Not Friendly	2
		Attributes - Fun	43
		Attributes - Not Fun	2
		Concerns - Uncanny/weird/creepy	68
Needs to be met			247
	Interaction		143
		Needs - Importance of verbal interaction	7
		Needs - Importance of soft/fluffy touch	35
		Concerns - Short term interaction/Novelty/Gimmicky	44
		Concerns - Limited responsiveness/interaction/evolution	57
	Robot Aspects		104
		Personalise to Personify	8
		Needs - Importance of independent movement	15
		Needs - Importance of a face	34
		Needs - Importance of being animal-like	16
		Needs - Importance of sound	1
		Concerns - Movement is clumsy/stilted/slow	18

Table A1 Qualitative codebook from analysis of participant rationale for robot perception ratings.

Real vs Robot Pets Codebook

Category	*Sub-Category*	*Code*	*Count*
Real Pets			535
2-4	Affective Social Pets		194
		Pet - Social - Social outreach by pet	43
		Pet - Social - Presence is reassuring/comforting	18
		Pet - Social - Mutual Trust	20
			14
		Pet - Social - part of the family	12
		Pet - Social - Companionship / Reduce loneliness	16
		Pet - Social - Emotionally close to pet	40
		Pet - Social - Not very close to pet	15
		Pet - Social - Bonding and closeness evolves	14
		Pet - Social - Adds life to the home	2
2-4	Activities with Pets		109
		Pet - Activity - Affectionate touch / holding	49
		Pet - Activity - Playing	11
		Pet - Activity - Exercise / Walks	10
		Pet - Activity - Training	6
		Pet - Activity - Sleep	16
			3
		Pet - Activity - Relaxing/Winding down	14
2-4	Pet Qualities		52
		General - Two-way affective interaction	19
		Pet - Aspects - Are Individuals / have agency	18
		Pet - Aspects - Emotional Intelligence	8
		Pet - Aspects - Pet is reactive to me	3
		Pet - Aspects - Pet is protective of me	2
		Pet - Social - Pets have advantages over humans	2
2-4	Friction of Pet Ownership		30
		General - Dependency Anxiety	5
		Pet - Friction - Clingy/High Maintenance	8
		Pet - Friction - Anxiety about pet wellbeing	16
		Pet - Friction - Scary Interaction	1
2-4	Impact of Pet Ownership		150
		Pet - Self - Taught me responsibility	8
		Pet - Self - Let me being caring / cultivate	37
		Pet - Self - Rewarding relationship	13
		Pet - Self - See them flourish	18
		Pet - Self - Benefit Mental Health	3
		Pet - Self - Pet is a part of me	3
		Pet - Self - Can be low maintenence	1
		Pet - Self - Makes me laugh/feel happy	12
		Pet - Self - There for me when times are hard	24
		Pet - Self - Mourn/miss pet's absence	17
		Pet - Self - Pet is part of my schedule	10
		Pet - Social - Pet is a social catalyst	4
Robot Pets			689
	Inherent Differences of Robot Pets		179
		Robot Pet - Real / Sentient vs Not Real / Programmed	87
		Robot Pets - Concerns - Real pets involve upkeep, responsibility, scheduling	20
		Robot Pets- Concerns - One-sided Relationship	20
		Robot Pets - Concerns - Value in Real Pets having Finite Lives	10
		Robot Pets - Concerns - Real pets are an organically built relationship/bond	16
		Robot Pet - Aspects - More accessible than real pets	26
	Prospective Robot Pet Activities		31
		Robot Pet - Activity - Playing or Training	15
		Robot Pet - Activity - Relaxing/Winding down	4
		Robot Pet - Activity - Let me be caring	4
		Robot Pet - Activity - Walk/Exercise	5
		Robot Pet - Activity - Sleep	3
	Impressions of Current Robots		53
		Robot Pets - Past Positive Experience	6
		Robot Pets - Social - Could personify robot	5
		Robot Pets - Concerns - Uncanny valley / too realistic / creepy	6
		Robot pets - Concern - Toys, not pets	10
		Robot Pets - Concerns - Its for specific users	8
		Robot Pets - Concerns - Lacking context from real pets	1
		Robot Pets - Concerns - Not Sure What is Needed	7
		Robot Pets - Concerns - Tech and Privacy Concerns	6
		Robot Pets - Aspects - Fun / Entertaining	2
		Robot Pets - Aspects - Friendly / Appealing	2
	Need for Complexity and Fidelity		80
		Robot Pets - Needs - Flaws / Make Mistakes	3
		Robot Pets - Concerns - Gimmicky / Figure Out How It Works	4
		Robot Pets - Concerns - Limited expression / interaction / emotional evolution	32
		Robot Pets - Concerns - Lack unpredictability / personality /complexity	41
	Desirable Robot Outcomes		59
		Robot Pet - Unconditional love / support	4
		Robot pets - Needs - Want them to be useful	25
		Robot Pet - Needs - Part of my schedule	2
		Robot Pet - Social - Social Outreach / Affection	5
		Robot Pet - Social - Companionship	11
		Robot Pet - Social - Presence could be reassuring/comforting	12
	Desirable Robot Form		96
		Robot Pet - Needs - Warm	12
		Robot pets - Needs - Independent/expressive movement	34
		Robot Pet - Needs - Importance of face	2
		Robot Pets - Needs - Importance of sound	1
		Robot Pets - Needs - Personalised to owner preferences	5
		Robot Pets - Concerns - Lacks Physiological Features of Real Pets	4
		Robot Pet - Aspects - More Realistic Pet-like Form Factor	28
		Robot Pet - Aspects - Robot-like Form Factor	7
		Robot Pet - Aspects - Size / Weight	3
	Desirable Robot Interactions		187
		Robot Pet - Needs - Affectionate touch / holding / soft	36
		Robot Pet - Needs - Agency/Interior Lives	33
		Robot Pet - Needs - Evoke Pet-like Interactions	17
		Robot pets - Needs - Create emotional closeness/attachment	9
		Robot Pet - Needs - Improved Interactivity/ responsiveness / recognise emotions	32
		Robot Pet - Needs - be emotionally expressive	10
		Robot Pet - Needs - Affectionate / Evolving Intelligence	35
		Robot Pet - Needs - Social Outreach	6
		Robot Pets - Needs - Importance of verbal interaction	5
		Robot Pet - Social - Talk to the robot	4

Table A2 Qualitative codebook from analysis of participant impressions of robots as pets and in relation to pets.
