# Supplementary material for: “Cute Companion” or “Awful Thing”: Understanding Individual Differences in Attitudes to Social Robots as Pets
Source: Int J Soc Robot. 2026 Nov 2;18(8):115. doi: 10.1007/s12369-026-01453-4 (PMC13727923; doi:10.1007/s12369-026-01453-4)
Supplement: Supplementary file 3 [file 12369_2026_1453_MOESM3_ESM.zip › Data IJSR/Qualitative/AZRAM Survey Qualitative Comparing Robots.docx]

**Rate how receptive you are to NAO as a potential household companion from 1 – 7. Why did you give this rating?**

| **PID** | **R** |  |  | **NAO Receptive Rating Rationale** |
| --- | --- | --- | --- | --- |
| P1 | 3 |  |  | still not real but more human like |
| P2 | 4 |  |  | I'm biased because I know NAO, but since I know that it needs to be programmed for interactions, and that it tends to malfunction a lot, I'm not sure if it would be super interesting to have as a companion. |
| P3 | 3 |  |  | It is just not useful to me, and has no emotions. feels more like a toy |
| P4 | 4 |  |  | I just think it looks like what we expect robots to look like very mechanical |
| P5 | 1 |  |  | It doesn't feel very useful. |
| P6 | 3 |  |  | Too human like. Not like a pet more like a flatmate... |
| P7 | 6 |  |  | It can talk back and interact with what you are saying. |
| P8 | 1 |  |  | It looks like it's trying to be human but it moves slower than a pensioner on the tills at tesco who just realised they needed to pay for their shopping and hunt through a massive bag so they can pay in change, while a massive queue forms behind them |
| P9 | 2 |  |  | This guy falls into a weird void where it's not useful enough to aid in household chores and it's not whimsical enough to be fun. It's demonstrative of some really great progress in robot movement technology, but ultimately it seems like a gimmick with little value to consumers beyond the novelty of these technological developments. |
| P10 | 4 |  |  | I feel like it is more of a toy/curiosity than a companion. |
| P11 | 1 |  |  | What is the purpose of it. Something that is so slow and can recognise a ball and get up if it falls over. It's so slow and the commercial gives no information as to what it is supposed to be. Is it educational or just something as a joke. It has an adult playing with it. I would think even a child would quickly tire of it. In act I doubt they'd even make it to Boxing Day as a Christmas present before becoming bored of it. |
| P12 | 4 |  |  | I gave higher rating because it has a cute looking, but it's behaving like an old person and I doubt he can take care of me or give me good company. And I also feel kind of unsafe when a large amount of personal data like the environment in my house being exposed in that way. |
| P13 | 2 |  |  | While it’s an amazing bit of engineering, it’s not really doing anything particularly interesting or useful. |
| P14 | 1 |  |  | It seems like a toy. The approximation of agency, or of having a life of it's own, is unconvincing like a parlour trick. |
| P15 | 5 |  |  | This robot seems much more...robotic. I feel like I had a robot toy as a child that was about as lifelike as this, and compared to Cozmo this one feels much less charming, much less like a real creature. |
| P16 | 3 |  |  | Visually it looks friendly enough, but the movement and voice are too uncanny for me. |
| P17 | 6 |  |  | well designed, well programmed interactive robot |
| P18 | 6 |  |  | Since I can talk and interact with NAO I feel that it would be quite a good household companion, although mostly for company as based on the video I don't know how useful it would be accompanying me in e.g., household tasks or activities like playing more complicated games. |
| P19 | 4 |  |  | The information shown is insufficient to make that judgement. At the moment I see no reason to exclude the robot from the household, nor any to include it. |
| P20 | 1 |  |  | It’s another glorified toy. Completely useless. It can’t do anything useful for me. A time waster. |
| P21 | 4 |  |  | I am interested to see how the robot works but am unsure how it would be of benefit in a busy household where there is constant activity. |
| P22 | 4 |  |  | It’s all in the programming. |
| P23 | 2 |  |  | The robot is interactive but not fluffy or enjoyable to stroke. I would not like to cuddle this robot at night. |
| P24 | 4 |  |  | I’m not generally an early adopter. Better to wait on the problems being ironed out first. |
| P25 | 2 |  |  | At this stage it seems like a clever toy rather than something that could provide companionship. I would rather have a living pet that can provide joy and affection. |
| P26 | 2 |  |  | Really don't feel comfortable with any robots with internet access. It poses far too great a security risk. Also development of responsive motion really feels like it will inevitably be used by military and law enforcement. |
| P27 | 3 |  |  | I don’t like that its body moves and is responsive but its face is static. I don’t like its jerky movements either, it makes me uneasy for some reason, maybe as if it makes the robot seem unpredictable or not very competent. |
| P28 | 2 |  |  | I wouldn't like this in the home at all. I guess it feels too sharp? Hard plastics and really stilted movement, etc. Something about the way it moves feels like it would consistently fall over. Uncanny? It is undeniably cool technology, though. And the face is a bit cute. But not for me. |
| P29 | 1 |  |  | Interacting with this robot looks like it would require close to but not the same as modeling another mind and engaging with an animal or person. This seems like it would be the worst of both worlds as it will be tedious and tiring in ways similar to interacting with a person or animal but with extra friction due to the limitations of the technology and with less payoff. The capacity of such a system to grow and surprise you with reflections of your inputs and combinations of them with its other experiences and ideas is extremely limited compared to almost any mammal. Especially if it stops receiving software updates, or the company goes out of business and shuts down their authentication servers. |
| P30 | 1 |  |  | I cannot see myself ever having the use of a robot like this in my life, but it is truly interesting to see. |
| P31 | 2 |  |  | It looks and moves like a baby. I don't want a baby. It's very slow and doesn't seem interesting. And I don't like that it could recognise my face. |
| P32 | 6 |  |  | I can see how it has the potential to become a companion much like an animal , hence the scoring I gave |
| P33 | 2 |  |  | Looks kind of tacky. Movement is so slow and cumbersome. Can't really think of any reason I'd want this around. It can't do anything useful, and it isn't nice to look at. |
| P34 | 5 |  |  | Looks like a very useful multi purpose built robot. However, it seems everything is not pre programmed and you would most likely have to program it yourself |
| P35 | 4 |  |  | I don't know if I would need her |
| P36 | 5 |  |  | Would be interesting to have around |
| P37 | 1 |  |  | I wouldn’t have any use for it. |
| P38 | 1 |  |  | It's responses are algorithmic, canned in nature. It more comparable to a Preying Mantis in intelligence than a dog. |
| P39 | 7 |  |  | He’s just a lil’ guy. I want one now! |
| P40 | 5 |  |  | Its a cool toy, but not a thinking personality |
| P41 | 4 |  |  | This is a great toy! I want one! Don't get me wrong, it is NOT an household companion, but if it's freely programmable, it would be one of the greatest toys I've ever seen. Still, everything I've said regarding the previous two models applies. |
| P42 | 2 |  |  | For a child it would be great toy. Other than that nope |
| P43 | 3 |  |  | I don't think it could really understand my tone or emotion when speaking , I feel like it would act the same if I am sad or happy, and I don't want this in a household companion. On the other hand, it could be useful sometimes to not feel completely alone. |
| P44 | 1 |  |  | It's painful to watch how slowly and jerkily it moves. It has an annoying voice. The vide didn't show anything useful or interesting the robot is able to do |
| P45 | 4 |  |  | It can't really do much, and affection wouldn't really be its kind of thing. Sort of feels like it has lots of features but not so many uses. If I want to chat, then ChatGPT does that already. If I want something helpful, a roomba is better. If I want a companion, then the previous two robots would probably do that better. But at the same time, the proximity seems like it would be comforting, and the wandering around and responding to voice makes it seem a lot more real, like there's really someone else here with me. |
| P46 | 7 |  |  | As I watched the video, NAO can do lots of things as kicking the ball, able to talk with human. It is very interesting for me. If I would be a customer, I would like to buy one of them. |
| P47 | 1 |  |  | As before - robots are not companions for me. They are perhaps entertainment or help, but not companions |
| P48 | 7 |  |  | It seems fun! |
| P49 | 4 |  |  | I gave the neutral response because whilst I don't dislike it (and thus wouldn't be actively against it), I just have no interest in it as a potential household member. |
| P50 | 6 |  |  | Having a more humanoid robot fits what I think of as a 'robot household companion' . It would fulfill a different role to one like Qoobo for example, more of an assistant than a pet |
| P51 | 5 |  |  | Because it looks good |
| P52 | 2 |  |  | It's very 'robotic' in it's slow awkward movements, static plastic face, and humanoid form, so it's not very appealing in an emotional or social capacity, it seems more functional than social, and I don't see uses for it around the house |
| P53 | 5 |  |  | because he can do more things, |
| P54 | 2 |  |  | Cold and unlovable. |
| P55 | 5 |  |  | Some interaction looked fun |
| P56 | 3 |  |  | The eyes changing colour feel like they could turn red, indicating a turn to villainy |
| P57 | 5 |  |  | i could imagine nao being useful to assist with daily life tasks (reminding me of tasks to complete, making lists (e.g. shopping list). It is somewhat good he is stuck to one place,and thus doest not distrub the privacy of my own home. |
| P58 | 4 |  |  | it looks like a human |
| P59 | 7 |  |  | Nao can plays with young children and he is also a friendly robot. |
| P60 | 7 |  |  | from the video that shows its ability to see, speak, move, and dance |
| P61 | 5 |  |  | family friendly |
| P62 | 5 |  |  | I feel normal. |
| P63 | 6 |  |  | I’m feeling comfortable with this robot |
| P64 | 1 |  |  | i dont like humankid robot |
| P65 | 4 |  |  | looks ok but not that interesting to me |
| P67 | 5 |  |  | It's robotic less natural |
| P68 | 2 |  |  | too slow and not cute and not really useful |
| P69 | 7 |  |  | Its look really smart and receptive |
| P70 | 5 |  |  | I love robot |
| P71 | 6 |  |  | It might help me clean my room |
| P72 | 5 |  |  | The robot seems fine but lack of emotions |
| P73 | 2 |  |  | The robot feels like an infant in a grown up's body. Such a disconnect infuriates me for some reason. But since it is made by Softbank, I trust it with all of my money. |
| P74 | 7 |  |  | This one look fun to play with |
| P75 | 1 |  |  | useless |
| P76 | 6 |  |  | Good |
| P77 | 5 |  |  | It feels like it is a younger sibling or a companion. |
| P78 | 7 |  |  | I do want a worker bot if it ever get more fluid |
| P79 | 6 |  |  | Look like an assistance robot or a companion to play with when bored. |
| P80 | 6 |  |  | Does not look harmful and could help in someway |
| P81 | 5 |  |  | It a bit feel neutral |
| P82 | 5 |  |  | It shape like a human very uncanny |
| P83 | 6 |  |  | i think this robot has a lot of interactive functions and its movements feel pretty natural |
| P84 | 6 |  |  | Looks like an assistance robot for a household use |
| P85 | 5 |  |  | I would give this a try it feel fun to play around with, a lot of cool features too. I feel the same way a Cozmo but I just feel that the movements seems slow I would not really prefer this but I would defiantly give it a try |
| P86 | 6 |  |  | I feel like this robot is a smart robot but it definitely need some more function to help human performs tasks more efficiently |
| P87 | 1 |  |  | Too freaky. It lives somewhere in 'Uncanny valley'. |
| P88 | 2 |  |  | I do not think I would have a use for such a robot |
| P89 | 4 |  |  | It seems it is moving awkwardly. Also never had any experience like this before. |
| P90 | 1 |  |  | I think this one looks particularly robotic so I am less attached to it than the animal ones |
| P91 | 2 |  |  | It feels very fake, idk how to explain it |
| P92 | 7 |  |  | Again, had very similar toys in the home that encourage learning and interaction. They do have a short shelf life in terms of engagement with children, which appears to have been the target audience initially |
| P93 | 3 |  |  | I don’t dislike it but I see no use for it |
| P94 | 2 |  |  | this is like a 90s robot.. awful design, speech, movement, everything. Disney animatronics look better than this |
| P95 | 1 |  |  | same as the Roomba |
| P96 | 4 |  |  | If I were to have a robotic companion in my home, I would prefer it wasn’t shaped like a person |
| P97 | 5 |  |  | cute, not my type of thing but cute |
| P98 | 4 |  |  | It’s creepily very smart and to me it’s too much alive and it weird me out a bit |
| P99 | 6 |  |  | It looks like it's in permanent shock. again, a good kids toy. |
| P100 | 4 |  |  | Looks interesting but somehow I feel the more human like robots become the less appealing they are. Much prefer the previous one! |
| P101 | 1 |  |  | it gives me uncanny valley vibes as it seems like its trying to become a 'better human' and i am not a fan of that idea |
| P102 | 3 |  |  | I feel like this is more of a toy than a companion |
| P103 | 1 |  |  | Its intelligence and interactivity seem uncanny to me and I wouldn’t want something like this in my house as for all I know it could be spying on me or plotting against me. |
| P104 | 5 |  |  | Again, I can picture this being a fun toy to interact with and chat to, but I can't picture forming the same kind of attachment as I would to a living animal. |
| P105 | 4 |  |  | I couldn’t review this was as ironically the link for the video asked me to sign in because it thought I might be a robot. |
| P106 | 5 |  |  | Its not that smart but it is a voice |
| P107 | 1 |  |  | Overused design. Again I think this would scare my cat. I think it would get in the way and it’s small I would probably stand on it. The design is similar to small robots I’ve seen in the past. Noting exciting about it |
| P108 | 4 |  |  | only would for a child |
| P109 | 4 |  |  | I think it is cute and kids would like it, but I wouldn't buy one |
| P110 | 6 |  |  | Robots are cool |
| P111 | 5 |  |  | has limited capacity to respond |
| P112 | 1 |  |  | Kids would see it as a toy. |
| P113 | 1 |  |  | I would not use a robot as a companion |
| P114 | 1 |  |  | I really hate the idea of AI and robots becoming part of our day to day lives. While it could be seen as a natural progression of mankind's ... well, progress, there is something in me that truly detests all of this technology, full stop. It's not interesting or cool to me, just uncomfortable and undesirable. |
| P115 | 5 |  |  | Despite being an incredible piece of technology, it lacks the warmth and affection I would want from a companion |
| P116 | 1 |  |  | I have no need of such companionship. |
| P117 | 3 |  |  | I wouldn't want a mechanical robot at home to be honest I find it a bit creepy especially as it mimics the human body. |
| P118 | 2 |  |  | In simulating a human body, despite how impressive it is, it would more more functional if it were on wheels or treads like one of the previous examples. It seems it is designed to be more like a human than useful and functional |
| P119 | 1 |  |  | I don't find a reason to have such a robot as a companion. |
| P120 | 2 |  |  | I find that Nao is too much like Toys I have seen growing up, while more advanced than those toys, it just doesn’t have the immediately personal connection that I'd want in a household companion |
| P121 | 5 |  |  | It's missing a more alive feeling, seems cool but wouldn't be something I'd have interest in for long. |
| P122 | 4 |  |  | The appearance is too mechanical |
| P123 | 4 |  |  | Ehhh seem's cool but again seems like a expensive toy, maybe a cool gift for a kid or something |
| P124 | 4 |  |  | What differs humans and robot is learning from the past mistake, humans constantly take care of emotions and past experience to relate and sympathize with other humans, but a non living organism cannot fully understand that. |
| P125 | 6 |  |  | I like this robot as I think it could be interactive with kids and adults of all ages. |
| P126 | 1 |  |  | I don't care for robots |
| P127 | 1 |  |  | Unsure why but the features of it made me somewhat uneasy. I think it is due to the speaking and the vision |
| P128 | 1 |  |  | I do not see the appeal |
| P129 | 4 |  |  | Still just a robot. This one is more complex and will probably end up looking after me when I'm old. Has a cute face but is still a machine |
| P130 | 1 |  |  | Looks hard to keep clean. Too many little nooks and crannys. Probably be packed with dust in a week |
| P131 | 4 |  |  | not particularly captured by any of its features |
| P132 | 4 |  |  | Still suspicious of intentions of creators |
| P133 | 6 |  |  | The idea doesn’t creep me out but it lack human connection and I don’t like the idea of myself being dependent on anything in my household |
| P134 | 2 |  |  | Same to before - OCD potentially getting worse due to obsessive thoughts about it killing me, expenses, no use for it. |
| P135 | 1 |  |  | i am scared |
| P136 | 1 |  |  | Technically interesting (it’s my field) but functionally irrelevant as shown |
| P137 | 5 |  |  | i like it does what a companion would need to do but looks to robotic for me if you could give it the cuteness of the seal one with the functionality it would be perfect |
| P138 | 2 |  |  | It's a bit boring and slow |
| P139 | 4 |  |  | Not as friendly looking as the previous entries |
| P140 | 7 |  |  | I think it'd be great to have a wee robot companion about the house, you could ask it questions and just have fun with it. |
| P141 | 3 |  |  | I would rather have a pet. |
| P142 | 3 |  |  | idk |
| P143 | 5 |  |  | I think it’s for robot in general, I’m still not ready for a robot companion and I don’t feel the need of a companion. |
| P144 | 1 |  |  | Just does not connect with me at all, on any level. |
| P145 | 2 |  |  | Maybe it was just the video but the robot didn't appear to able to do much other than walk around and grab things, not useful or entertaining |
| P146 | 6 |  |  | It looks fun, but again it's more of a toy than a real companion. My 10 year old would love it, but I think the novelty would quickly wear off as an adult. |
| P147 | 1 |  |  | I’ve no need |
| P148 | 4 |  |  | A 'companion' is a strange word for it. I don't believe this robot to be either alive or dead, but it's more lifelike that the seal or the tail. Could it be useful for the everyday task? Sure. |
| P149 | 4 |  |  | It will be good for children but it is a little unexpressive |
| P150 | 1 |  |  | Junk plastic |
| P151 | 1 |  |  | Same as previous answer. |
| P152 | 2 |  |  | As a companion this robot seems somehow childlike and the idea of a robot child is unsettling. |
| P153 | 1 |  |  | I have directly interacted with NAO robots many times in the past--they never seemed to work as intended, always broken, and surrounded by frustrated and embarrassed people. I have extremely negative associations with them, and also just think the are pointless and fragile toys. |
| P154 | 2 |  |  | Far to classic robotic, almost intimidating |
| P155 | 1 |  |  | Humanoid robots, especially ones that mimic toddlers are creepy. |
| P156 | 7 |  |  | For Nao, I can imagine some tasks which could be reliably delegated to it. For instance, some tasks which are performed with voice recognition and are possible to do with an intelligent voice speaker or virtual agent. Having the presence of a humanoid robot (for me personally), would make it more likely for me to use current available off-the-shelf pseudo-AI type applications (available on phones, voice speakers etc), simply because of the physical presence of the machine. |
| P157 | 6 |  |  | Friendly appearance. Clever design/interactive |
| P158 | 6 |  |  | NAO's lookalike is somewhat lovely and close to humans in a manner that does not disturb or arise any inner feelings of rejection, which might be the case for geminoids. |
| P159 | 1 |  |  | It looks like an early evolution of an autonomous robot in the tradition of old sci-fi: not yet capable of being an effective home co-worker; and without the inner life and personality of a pet. |
| P160 | 4 |  |  | because I like it |
| P161 | 2 |  |  | Slow, less emotionally engaging and too human. This would be a very suitable robot, in the original sense of a mechanical servant, but a poor companion. |
| P162 | 1 |  |  | I see this as less of a companion, more of an interactive toy - it's quite cool! That said, I would hate to have it kicking around my house, it just feels weird |
| P163 | 5 |  |  | Provides entertainment at the least |
| P164 | 5 |  |  | Another fun robot that you can interact with, and it also has a 'life of it's own' as it is able to respond, see, and interact back. |

**Rate how receptive you are to Paro as a potential household companion from 1 – 7. Why did you give this rating?**

| **PID** | **R** | **Paro Rationale** |
| --- | --- | --- |
| P1 | 1 | i personally just hate paro I’m sorry |
| P2 | 6 | I have cuddled with Paro before and I love how responsive it is. It feels like it hits the right balance between not being too complicated (for a social robot anyway), but still displaying enough social signals that I want to continue interacting with it. |
| P3 | 4 | It just feels like a middle ground. I know it is not alive but is modelled after an animal. |
| P4 | 4 | I just became more unsure as I answered things so went neutral |
| P5 | 4 | A stuffed animal can blink or feel a little scary when it moves. |
| P6 | 5 | It is nice and cute, but seems too stagnant, non-moving. I would have to pick it up and put it somewhere. I would probably prefer something that could come to me by itself. |
| P7 | 6 | The way it responds to touch makes it feel more like a real pet |
| P8 | 2 | Responds to pets with a smile! Love it |
| P9 | 7 | Honestly, seals are my favourite animal and have been my whole life so the rating is probably more about that than any robot features it has. I actually had a harp seal stuffed animal growing up that looked really similar to this lil robot guy, so it's also probably a bit of nostalgia. |
| P10 | 7 | Very similar to qoobo, I think it seems very fun as a companion. It has nothing against it, save basic functionality. |
| P11 | 1 | It was cute until it became animated then the creepiness crept in. If it was just a soft toy fine but really it just plays on your emotions with the needy high pitched whine. Plus what is it with the dummy? It's like trying to anthropomorphise a seal. Throw it a toy fish for goodness sake. |
| P12 | 2 | I really can't like it, maybe because of its looking or mechanical behaviour. And it's blinking in a really weird way. |
| P13 | 1 | It’s big, doesn’t do much, and the power cable binkie is downright creepy. |
| P14 | 1 | It looks like a more expensive version of a furby/one of those interactive dolls. As a facsimile of a living animal it is wholly unconvincing. |
| P15 | 3 | Because Paro can't move on its own, it feels like it would provide less of a 'pet-like' experience, and be more of an ornament? I'm also just not that enthusiastic about having a pet seal |
| P16 | 1 | I think the seal is very creepy, the noises, in particular, are very unsettling. |
| P17 | 7 | many of the features displayed (facial expressions, movement, vocalisations, furry coat) remind me pleasantly of cats |
| P18 | 7 | Similar to qoobo, I am very receptive to Paro as a household companion that serves as a form of easy pet, something to cuddle and keep on your lap. While the shape of Paro is maybe a little wieldy and less 'cuddle-able', I think its responsiveness and sweet face make up for that in how appealing it is. |
| P19 | 2 | A toy. It doesn't do anything that I might want it to do. |
| P20 | 1 | I can’t find words to describe how awful this thing is. A total waste of time and resources. Vomit inducing tat. |
| P21 | 4 | A living pet can be unpredictable, which brings with it reactions and actions which require a two way relationship that requires reciprocity. Paro would allow the human to be in control, which would suit an individual who may find caring for a living pet challenging. |
| P22 | 1 | It’s a fluffy electric version of those dolls that used to wet themselves - but without the engagement. I’d be reaching for my Norwegian Sealers Club after 15 minutes. |
| P23 | 6 | It is fluffy and has a face and makes noises which is cute but its movements still look mechanical. |
| P24 | 1 | Not an early adopter. |
| P25 | 5 | It’s cute but seems pretty useless and bothersome. |
| P26 | 5 | Unsure. Seals are very cute! |
| P27 | 1 | It’s cute but it’s supposed to resemble a baby seal, which doesn’t do much, so it doesn’t have many features that I could tell. The noise it makes is annoying little annoying. |
| P28 | 2 | The sound is horrible, but I guess that is the sound the seals make! The movements feel too mechanical and the reactions too simple. I think I'd have a hard time escaping the "machine in here" feeling. It does look cute though. |
| P29 | 1 | This seems like an improvement on a cuddly toy. The sensing and movement could add to the immediate pleasant sensory aspect of it being a soft thing. |
| P30 | 6 | I think something like this would be perfect for older and lonely people |
| P31 | 4 | It doesn't seem like it can do much, and looks a little Uncanny Valley. However it's mostly at least cute, and I like the fur. |
| P32 | 1 | It doesn't do much to be honest, it would be okay to stroke or cuddle. |
| P33 | 4 | It's kind of cute, but I found the noises it was making to be quite annoying. |
| P34 | 3 | The "pet" robot doesn't feel as responsive/ a lot of delays in its mechanicals movement. |
| P35 | 1 | Seems I don't like furry robots |
| P36 | 5 | More like a creature than the Qoobo but more mechanical |
| P37 | 1 | It just seems like a toy. It doesn’t have emotions like a real pet. |
| P38 | 4 | It triggers human emotions by mimicking a baby seal, but it is still just a robot. It's reactions will not change over time. It doesn't recognize individuals. |
| P39 | 6 | While I’d still love to have one, I feel it’s a bit more unpredictable than the others, perhaps because I’m unfamiliar with the behavioural patterns of seals, haha! |
| P40 | 5 | Its a fun toy |
| P41 | 1 | Boring, immobile, predictible. After you've has this thing for a few months (maybe even just days or weeks), you'd be probably so bored that you'd put it back in its box and store it in the attic with all the other over-prized crap you've collected over the years. Why is this even a question? This is a pathetic attempt. Tamagotchi-level. Have you've ever played "Creatures"? That was at least interesting enough to play a few hundred hours with. |
| P42 | 1 | It is hard to take robots seriously, they maybe a good toy to pass time for small children but at the end it is just a robotic device made by humans who was programmed to act accordingly. It’s very hard to be emotionally connected to things which are not alive. I could use the analogy of killing people in real life vs in video games. In real life I can't even imagine doing such a brutal act against another human, whereas I can kill people in video games without feeling much remorse because the former is reality while the latter is just virtual environment or artificial beings. |
| P43 | 5 | Because it has a cute body and it looks like a teddy bear or a toy to play with. |
| P44 | 1 | It's kind of creepy and makes annoying sounds |
| P45 | 6 | I'm concerned about the noise, but it seems quite nice. The way it tilts its head up and closes its eyes when you pet it contributes significantly to my positive feelings for it. That's something the dog I had when I was a kid always did, and I think it's just so wonderful and affectionate. It makes me feel like it would be a nice companion to have around. |
| P46 | 7 | This Paro is very cute!!! It is almost the same as real animal!!! She is very smart and sensitive as when we touch her head, she will close her eyes, It looks very real to me. |
| P47 | 1 | It's a toy, not a companion |
| P48 | 7 | It's cute |
| P49 | 5 | I think it's a nice thing so I'm on the positive side of the scale, though not necessarily something I would want which is why I didn't score it more highly. |
| P50 | 5 | Paro is very cute and could see it as fun household pet, but it looks quite limited in what it can do, compared to a real seal as a reference point. It's obvious that it is a robot/ is artificial when there is a real living thing to compare it too |
| P51 | 4 | Because it seems limited |
| P52 | 5 | There are some potential uncanny valley issues like with Qoobo, where it's close but not close enough to a real animal. Though the added expressivity of the face and voice add extra character to it, so would provide more engaging interactions. |
| P53 | 2 | i don't feel any utility for me, so i would not have it |
| P54 | 2 | Toy like |
| P55 | 5 | It was cute so kind of appealing but still felt my interaction with it would be rather meaningless |
| P56 | 3 | Personally, I do not like the design |
| P57 | 4 | I have been working with PARO as a researcher and thus find that the robot is not to diverse in its interactions. i would require paro to be more interactive and response to imagine having it in my own home. |
| P58 | 7 | it is cute |
| P59 | 7 | I would say that Paro seems like a real animal at the zoo. It can interact with you like the real pet. When we touch Paro's head, he closes his eyes as real animal. he is very cute. I would buy one. |
| P60 | 6 | from the information from the video |
| P61 | 7 | cute |
| P62 | 5 | It's cute. |
| P63 | 6 | It look cute |
| P64 | 1 | why would i want a seal? feels unnecessary |
| P65 | 4 | Very cute but still feels a bit mechanical to me |
| P67 | 5 | it's cute but can be more natural |
| P68 | 6 | If it was given to me for free, I wouldn’t mind it following me around asking for head pats |
| P69 | 5 | Its feel receptive and smart enough |
| P70 | 3 | Not sure |
| P71 | 7 | It might be a good snuggler. |
| P72 | 4 | Because it looks like it has emotions but felt dull sometimes |
| P73 | 2 | The linear dilation of the eyelids and tail weird me out |
| P74 | 4 | It is no different to toy |
| P75 | 7 | I like seal |
| P76 | 6 | Cute |
| P77 | 6 | Since it looks more like an animal/pet, I feel like it can be a companion like a pet. |
| P78 | 7 | look cute |
| P79 | 7 | Looks very cute. Good for healing mind. |
| P80 | 5 | Pets |
| P81 | 5 | i like animal robot |
| P82 | 3 | I still feel like a robot. The movement the respond and it did not have that much interaction |
| P83 | 2 | I feel very uncomfortable looking at this robot |
| P84 | 1 | It is still clearly a non-living thing. Doesn't really look like an actual animal anyways. |
| P85 | 6 | I think this one is nice, it feel more alive with movements in the eyes. I think it also is cute it would be a potential. It would also be nice on the bed with seems like it has a purpose to be a companion pet based on a real world. |
| P86 | 3 | this robot can help with a lonely person because of the responsiveness of the robot it self, but it might not be for me |
| P87 | 3 | Cute facial expression. |
| P88 | 2 | I am not sure that this is something I would like |
| P89 | 4 | Never experienced anything like that. |
| P90 | 6 | I would happily have one in my home |
| P91 | 1 | It just seems like a toy |
| P92 | 6 | . |
| P93 | 1 | Very obviously programmed to respond in certain ways, also useless really. |
| P94 | 5 | Now this! I loove seals. The robot is fluffy, its cute, somewhat interactive. It by NO MEANS could replace a pet, it a more alike to a temporary gimmick and not a long-term solution but its a step in a more "animal like" direction and feels more familiar. |
| P95 | 3 | as a toy it's better, so it interaction is more effective but I still don't see this a pet or like a living thing, the same with the other two robots |
| P96 | 4 | It doesn’t move around or act on its own |
| P97 | 5 | I think he's adorable but I'm not sure my rabbits would like him:) |
| P98 | 1 | It’s very creepy looking to me with the squeaks and the slow eye blinking |
| P99 | 3 | Can't see the use in it again more like a kids toy |
| P100 | 2 | Not for me as a pet, maybe a children's toy though |
| P101 | 5 | i think it could represent all the good things about a pet, the love and affection it could give you but without the person needing the responsibility of actually taking care of a pet (feeding, walking, brushing it etc) but it gives me a sort of uncanny valley effect as i know its not real and just a machine |
| P102 | 4 | I am unsure on whether or not it'd be a suitable companion, as it doesn't provide much in terms of features |
| P103 | 5 | It seems like a cool toy that kids would like and could potentially have benefits for mental health but aside from that I don’t see much other usage or need for it. |
| P104 | 5 | I think it's a cute design (apart from the noises, which sound a bit artificial to me) and makes for an impressive toy. I definitely would have wanted one as a child, but I also think I would have gotten bored of it after a while. I can't imagine it holding up as a substitute for a live pet with a unique personality and with whom you form a reciprocal bond over time. |
| P105 | 4 | Seemed less put together than the other advertisements of the other robots, I feel like I didn’t get to know enough about it to make a full judgment |
| P106 | 5 | Its a skin suit |
| P107 | 2 | A little creepy. It might be nice for old people with dementia as I’ve seen something similar with cats in dementia centres. The design could be better and it could be enhanced by making sounds . |
| P108 | 4 | cute for children |
| P109 | 4 | I think of this as a Childs toy with limited usability which means it will be dumped within 12 months if not less. I think a Child's imagination does not need robotics, they spend enough time plugged in to screens without removing the imagination completely |
| P110 | 5 | It’s cute but a bit weird |
| P111 | 6 | Can respond to actions such as stroking, with actions resembling emotions |
| P112 | 1 | Kids. |
| P114 | 1 | If I had a child, it might make an interesting toy. I probably would've enjoyed a toy like this when I was a child. But I can't consider it a companion, again. |
| P115 | 6 | It looks more lifelike than the first example, but it still can't compete with a real-life companion |
| P116 | 1 | I don't feel the need for one. |
| P117 | 4 | I'm not sure. Maybe for a companion at work rather than at home would be nice! |
| P118 | 1 | It tries too hard to simulate a real animal, it is uncanny to me. It's responses are programmed and therefor do not create an emotional connection for me |
| P119 | 5 | It's because of the familiarity of the stuffed toy + being responsive to me. |
| P120 | 6 | I think Paro is a lovely recreation of something we see in the world at the moment, there was a lot of thought and care put into it for it to be a pleasant interactive robot. |
| P121 | 1 | It looks more like a toy for a child to play with rather than a household companion. |
| P122 | 4 | He is too stiff |
| P123 | 3 | Seem's like a expensive toy, and storage space is low |
| P124 | 1 | if you want to take care of animals, try to do with real ones, spend time with one that helps a living being. |
| P125 | 4 | I think this would be good for all ages. |
| P126 | 1 | I don't like mechanical plushies |
| P127 | 6 | It is far more life like with having responses to people like a real pet would. It feels far less artificial |
| P128 | 2 | More cute than the robot, more relatable |
| P129 | 4 | Kids would like this. But they would still know it's not real. |
| P130 | 1 | Creepy! Far too close to a real creature. Also i would be concerned about children seeing something like this and being more likely to approach a wild animal. |
| P131 | 5 | I worked with a paro at a job and it was a big hit with kids, however he got patted so much he started to smell. i may be biased from this memory. |
| P132 | 2 | Like a toy, useless |
| P133 | 1 | It creeps me out no |
| P134 | 2 | No use, expenses, it’s gonna kill me. |
| P135 | 7 | Because it's cute |
| P136 | 1 | A modern version might be useful in certain therapeutic environments, otherwise irrelevant |
| P137 | 5 | very cute but would need to move about and to feel more life like |
| P138 | 1 | The eyes and movements are a bit odd and slow |
| P139 | 6 | This reminds me of on of my brothers childhood toys, the addition of animatronics does make it more life like but I do wonder if it will effect the texture of the seal. Since you probably can't squeeze it quite the same |
| P140 | 4 | I don't feel as attached to this robot, it feels like it's just been programmed to respond rather than actually be interacting with you. I'd rather have a cat as it is a living breathing version of this type of robot. |
| P141 | 1 | I do not like the fact it is mimicking a baby animal. I find the noises it makes unsettling. |
| P142 | 4 | for a kid |
| P143 | 6 | Look cute |
| P144 | 4 | The fact it has a face, and responds is more attractive. but it is still not the same thing as a living breathing being. |
| P145 | 2 | It was pretty cute, but its behaviour was even more limited than the other robot so not that interesting |
| P146 | 2 | It seems like more of a toy than something that can give the affection, warmth, and love of a real and intelligent pet like a dog or guinea pig. My 10 year |
| P147 | 2 | My daughter had something similar when she was a child, she loved it, I didn’t! |
| P148 | 2 | I don't believe it replaces life. |
| P149 | 3 | It is cute but i don't like the noises that makes. They aren't pleasant |
| P150 | 1 | Spooky! |
| P151 | 1 | Same as previous answer. I also didn't like the mechanical movement of this robot or the repetitive sounds. |
| P152 | 1 | As a toy for children I maybe see in a household. However, as the robot invites you to pet it, it doesn't seem to be live like enough to bring a content feeling for me and instead presents to me as an unnerving feeling. |
| P153 | 1 | Coming from the generation that went crazy for Furbies, this seems very much the same--a fluffy skin over a mechanical toy that does a small number of actions. It seems silly to ask whether it can appear competent. The baby-like appearance definitely makes it seem dumber and sillier, because all babies share that. Something about it's movements make it seem like it is imitating a sick or dying animal, which is pretty off-putting on its own. |
| P154 | 4 | I like it for companionship and could see it working well. However, as it is very animal-like, it feels like it should have more emotion |
| P155 | 1 | Paro is possibly one of the most objectionable examples of companion robots. The idea that would farm out the emotional labour of caring for vulnerable people to a machine is emblematic of everything that is wrong with late capitalist society. Spending time with, and caring for people should be viewed as the most skilled, and highly valued role that anyone can have. Instead it is so undervalued that people cannot afford to do it themselves and corporations want to replace with animatronic teddies. Genuinely disgusts me. |
| P156 | 5 | I certainly would be receptive to having this robot as an additional pet, but I'd also be keen to run some experiments with it and others who may find their interactions comforting and beneficial. |
| P157 | 7 | Would be great for kids, friendly and fun |
| P158 | 2 | I've felt two contradictory feelings about it. On the one hand, I like its appearance, although the set of "behaviours" plus "appearance made me uncomfortable. That might be an uncanny valley due to its closer resemblance to a real pet. |
| P159 | 1 | I haven’t grown up with the experience of taking comfort from plush toys, and the addition of anthropomorphic animation doesn’t appeal. Maybe if I lived alone in a place where I could not have a pet, or adequately care for one, I might become used to it over time. But it seems designed more for novel cuteness, and maybe manipulative neediness, than helpful warmth or comfort. Or for finding and killing fish to cook. |
| P160 | 1 | I think it will be nice to have this robot for some people in need but I prefer having a living pets. |
| P161 | 4 | It cannot move, movement is a big advantage to a companion. It's very cute, and probably good to cuddle, but there's an uncanny valley effect going on that would make me even more aware that it isn't a real animal. I'd also hazard that it may be a bit too predictable in its reactions. |
| P162 | 1 | It feels like a slightly improved toy from my childhood - I think the knowledge that it is a robot prevents me from feeling empathy for it, and therefore it is not very comforting to me.   A crucial part for me of a household companion is their independence & their autonomy. It is a pain when they don't want to spend time with you, but that's the charm, knowing that it is a choice they make to hang out |
| P163 | 5 | Unique and perhaps a niche animal to be based off |
| P164 | 7 | Seems like a robotic pet, it serves a companionship purpose but it is still a robot and cannot be as lively as a real pet or human. |

**Rate how receptive you are to Qoobo as a potential household companion from 1 – 7. Why did you give this rating?**

| **PID** | **R** | **Qoobo Rationale** |
| --- | --- | --- |
| P1 | 1 | it is not "real" -- it is just a computer in a fursuit :) |
| P2 | 5 | I would love cuddling with this robot, but I feel like since it's missing a "head" ad more sophisticated social interactions, I may also eventually grow bored. |
| P3 | 6 | Really good. has animal features, i can use it and it will respond. it does not try to look like a specific animal, so no uncanny valley |
| P4 | 5 | i don’t know where i would use it - more to give to my dog |
| P5 | 2 | It serves little purpose and is more of a decoration. |
| P6 | 5 | It seems a bit passive, probably again because it cannot move by itself. It looks cuddly and cute, I like that and the tail gives a nice and noiseless feedback, which I like. If it could move by itself... I would love that... |
| P7 | 4 | I could see it being quite comforting in certain scenarios, in a similar way to a stress toy. I think because of it's limited functions it would not be a complete household companion, but the functions it does have could have longevity in terms of being a source of comfort. |
| P8 | 1 | From the video, it looks like a cushion with a fake rotating tail |
| P9 | 1 | It's not even cute. If I'm going to have a household companion, I'd like it to at least look whimsical and fun. This just looks like Davy Crockett's cap was necromanced. |
| P10 | 7 | I think it seems very fun. |
| P11 | 1 | It's creepy. If you want something to stroke buy a soft toy. I can understand if someone can't have a pet due to working hours but if you really want a pet a cat is pretty independent and doesn't need as much of your time. It reminds me of a tribble. A bad special effect. The people stroking it as if it's a real animal doesn't even make me laugh. It's in poor taste. Again, buy a soft toy instead. |
| P12 | 3 | It behaves too mechanically, nothing like a pet. I think there's no point in keeping something like this at home. |
| P13 | 2 | It’s like a cool teddy bear or doll - but I have my husband and pets to cuddle, so don’t see the need to have a wee furry mechanical thing too. |
| P14 | 2 | It's an approximation of a cats tail and fur. I suppose it could give some comfort, but it's hardly a substitute for the affection of a living animal. |
| P15 | 5 | I'm not sure. On paper it has the same issues that Paro has for me in that it would just hang out in one place almost like an ornament, but I find something more charming about it. It might be that the video has subtitles from its perspective, which makes it feel more alive? |
| P16 | 1 | I find the movement too unsettling, and the concept makes me uncomfortable. |
| P17 | 6 | Being furry is always a bonus in a household companion! Also like the moving tail. |
| P18 | 7 | I think I would really like petit qoobo as a companion because it seems very comforting like a pet but without the upkeep. I think I would use it a lot as on my lap when I'm working, or to cuddle in bed. |
| P19 | 4 | Obviously more limited that the last robot. I have no need of a toy that wags its tail, no matter how realistic the heartbeat sound is. |
| P20 | 1 | What is wrong with people? It’s like those pet rocks that were a craze for a while. Perhaps useful for those who have issues with social contact, but otherwise, utterly useless! |
| P21 | 5 | I can see that Qoobo would be comforting and reassuring for some, but I prefer a living pet. |
| P22 | 1 | Doesn’t seem to do much except be a cleaner less-demanding cat/rat |
| P23 | 5 | It is fluffy and I can stroke it but it doesn't have a face and does not make noises. I would prefer my real cat. |
| P24 | 1 | Not keen on modern tech as a phone. |
| P25 | 5 | I wouldn’t mind receiving one but it seems not that interactive from the video. It could have a cute face. |
| P26 | 6 | Have used one. I enjoy that it's not resembling an actual animal and is passive. I wonder if it could be given a kind of purring sensation as well? |
| P27 | 1 | It doesn’t have many features. I like that it wags its tail, but I think I’d get bored of it. I’d prefer if it had a face but it would need to not be creepy like a Furbee. |
| P28 | 6 | It's cute and nice. It feels like using technology for good in a world of bad. I'm not sure I would use it or need it since I have pets already, but I appreciate it existing. I'm interested in the technology behind it. |
| P29 | 1 | This seems like a potentially small improvement over a traditional cuddly toy. The movement of the tail is just on the edge of being lifelike enough that I think I would take a long time to be able to turn it out and have it not be a little surprising and distracting. |
| P30 | 4 | Not for myself, but at the retirement home where my mother lives they have a sort of robot cat that purrs and moves and all the old people just love it. |
| P31 | 1 | It looks like a cat with its head cut off. |
| P32 | 5 | It appears to be a comfort thing like a teddy or blanket. I can understand why it would calm people down however cannot be a complete companion as it doesn't do much , then again does it have to ? |
| P33 | 6 | It's cute, it reminds me of toys I enjoyed as a kid (Furby, some robot dog thing) |
| P34 | 1 | Looks like a soft version of pet rock. I wouldn't want this as a pet at all because of the material it is made from. How would one even clean that? |
| P35 | 1 | Uh, no. |
| P36 | 4 | I don't know what value it would add. |
| P37 | 1 | It looks strange. |
| P38 | 1 | Too simplistic. |
| P39 | 7 | Honestly? The tail wagging is adorable. |
| P40 | 4 | Its degree of responsiveness is minimal, the level of feedback is tiny |
| P41 | 1 | Is this a joke? If not, this is one of the most interesting and daring scams I've seen for a while. I was literally laughing out loud watching that video. You simply can't be serious. |
| P42 | 1 | Why would I spend time with a machine that has no real emotions, and it is programmed to certain tasks and react in a certain way. Imagine taking it to a repair shop and it is just dismantled. Very artificial! |
| P43 | 4 | It only could serve as a companion, but nothing to interact with. I didn't see that it has "functions". But it could be fun if I carry it with me all day |
| P44 | 1 | Is that a joke?? It's incredibly stupid and creepy |
| P45 | 5 | I'm not sure I would really be that likely to engage much with it. I also sort of wonder how artificial it would feel to have and treat as a companion which is ironically more of a concern with this than with the roomba. Maybe that's because this is meant to be a companion, so the extent to which it could provide authentic companionship is more of a concern. |
| P46 | 7 | She is very good as zoomorphic robot, if you want to but a pet but afraid of taking care of them. I would say that this is the best option to try to take care of pet as a real life. She is very cute and liveable. |
| P47 | 1 | The same as before - an artificial being is not a companion for me |
| P48 | 1 | I don't like that it doesn't have a face or eyes to mimic emotion. |
| P49 | 5 | As before |
| P50 | 6 | I can see Qoobo being a good substitute for a pet for someone, and portraying it with feelings as it reacts to you |
| P51 | 3 | It doesn't seem very interactive |
| P52 | 3 | It has some similarities to a pet in design and the intended interactions (stroking, reactivity) but it's potential uncanny valley territory where it's close but not close enough. I might rather have a less convincing companion like the previous bot, or a real pet. |
| P53 | 2 | it doesn't do much so i would not have a pet just to touch it and him to move slightly |
| P54 | 1 | barely anthropomorphic. no eyes is a big no. |
| P55 | 4 | Knowing it's not real gives me feelings of indifference |
| P56 | 3 | I personally do not like the design, in particular the lack of a face |
| P57 | 1 | While it is a lovely idea, for me personally the interactions are not varied enough that I would derive a significant benefit from it. I also think that as I am currently living with my partner I would reach out to him instead of a robotic pillow. However, if i was living alone I could imagine it being a nice tactile experience when I am craving a hug or physical company e.g. while watching a movie. |
| P58 | 5 | it looks cute |
| P59 | 7 | If you want to have a pet but you don't have any experience this robot would be the good choices for you because she is very friendly robot to play with. |
| P60 | 6 | Because this looks like a real cat and has a moving tail and heartbeat that make it look so real |
| P61 | 6 | cute but scary |
| P62 | 6 | It's kind cute and weird at the same time |
| P63 | 7 | It look so cute and lively |
| P64 | 1 | i don’t like how it looks and the vibe it gives. why does it not have eyes |
| P65 | 5 | looks comfy but feels weird to me for some reasons |
| P67 | 5 | Because i feel there could be room for improvement as it still feels more artificial |
| P68 | 5 | cute, wouldn’t mind having around |
| P69 | 4 | It’s cute |
| P70 | 4 | I don't like this kind |
| P71 | 2 | What is it even used for? I’m not sure what the application of this robot is. |
| P72 | 3 | There are only tails that mimic the behaviour of animals but beside that it is very mechanical to human |
| P73 | 1 | I would rather buy a pet rock. All the benefits and none of the drawbacks such as needing a charging port |
| P74 | 5 | It looks like I will get bored from it easily |
| P75 | 4 | not necessarily like or dislike |
| P76 | 4 | Depend on the user. |
| P77 | 4 | It doesn't really feel like a pet; it feels more like sort of a gimmick. |
| P78 | 4 | waste of money |
| P79 | 4 | Seems fluffy and soft to hold. |
| P80 | 3 | Doesn't do much |
| P81 | 5 | Feel like it a bit more receptive |
| P82 | 2 | It look very uncanny like a cat without a head that can move only it tail |
| P83 | 6 | I think the robot acts like the real animal |
| P84 | 5 | Looks promising and reacts to the environment even though the tail still look a bit mechanical. |
| P85 | 3 | I think that the it is more of an interactive pet, and I feel you need to be on your phone on one hand or like a gadgets. And I think that it more of a gimmick than a actual replacement, I would think i would be interested in it for a short period and forget about it. |
| P86 | 5 | this robot can give comfort to a person who uses it. it can definitely be useful for someone who is lonely but other than that there's not a lot of function implemented onto the robot it self |
| P87 | 2 | I'd be aware that I was deluding myself into thinking of it a conscious entity. |
| P88 | 2 | It reminds me of a cat, and I am not really a cat person |
| P89 | 1 | It is just awkward. |
| P90 | 7 | I'm not sure, I just like it and find it comforting |
| P91 | 1 | It's a coaster with a tail? |
| P92 | 1 | We can keep pets but for those who are unable too but desire too this can bridge the gap of companionship, comfort & some sense of positive receptive response. I like the idea. |
| P93 | 1 | Completely useless and creepy object |
| P94 | 4 | its.. functionally good. But aesthetically it just reminds me of a decapitated cat.. kinda gives me the ick.. dunno. |
| P95 | 4 | it is the best of the ones seen so far, it not all lifelike or would make me think of it as a pet but I could at least see it's use a toy or self help tool even if I would not use it personally  My main problem is that is clearly a programmed toy, even if it is capable of making it's own assessments, this is not visible |
| P96 | 1 | It makes me think of an alien trying to be a pet, I don’t like that its a pillow with a weird tail that moves very mechanically |
| P97 | 6 | another one I'd like to have keep me company as I work |
| P98 | 1 | To me it’s just a ball of fluff with a tail that moves |
| P99 | 4 | I would like this personally, its bit strange looking, not head or limbs |
| P100 | 1 | I don't have any strong feelings on this as an idea or if others find comfort from this but it would not be attractive to me. Part of the pleasure of owning a pet it the emotional connection that you share it's free will etc which this has none of. |
| P101 | 3 | i don’t like that its just a blob with a tail. if i wanted something furry in my house i can buy a real animal |
| P102 | 5 | I see this robot as a potential companion for those who cannot afford a pet or cannot have them for health reasons.  I also feel like it'd be good for those who need a tactile stimuli |
| P103 | 2 | I don’t really see any personal usage for this robot and it doesn’t seem to have any useful functionality. |
| P104 | 4 | I see this as a therapeutic tool, I think it would be pleasant to touch and the heartbeat rhythm would be calming, but it doesn't come off as "alive", just as a cushion. |
| P105 | 2 | This makes me very uncomfortable and I don’t know why. Maybe because it is literally a tail and a body rather than a whole pet! |
| P106 | 6 | Its comforting |
| P107 | 1 | I could never see myself carrying this in a bag like in the video not use it. It doesn’t look appealing and it would look better if it had a face !! I can’t see this catching on with adults !! Might be scary for children |
| P108 | 2 | would not buy but could be good for after the loss of a pet |
| P109 | 1 | It reminds me of a bizarre sex toy. I wonder if a male made it as a representation of the male organs, to get off on the idea that women may stock it. I am sure there are more realistic robot pets out there. |
| P110 | 6 | It’s like having a pet without the gross bits |
| P111 | 5 | can react with different emotive states depending on how the user interacts with it |
| P112 | 1 | Kids. |
| P113 | 1 | Second verse same as the first |
| P114 | 1 | It's not a living being. I don't consider something like this a "pet" or imagine myself being able to have any emotional attachment to it... I had furbies and robodogs... they're toys, not pets. |
| P115 | 2 | It doesn't actually do very much |
| P116 | 1 | Robots as they are do not appeal to me in terms of companionship. |
| P117 | 6 | I would get that. It's cute and not too creepy. |
| P118 | 1 | It is an artificial animal. I value animal connection because they are built over time and earned through respect. This is a fluffy motor |
| P119 | 2 | It would be useful only to help me sleep. |
| P120 | 6 | I find Qoobo very interesting as a potential household companion as its very unique in that its not immediately comparable to any living creature on this planet, it has the friendly qualities of most common pets that we have currently but its shape and design are soley unique to itself, so I think I would be more receptive to it than previous questions |
| P121 | 4 | It seems like it would be nice for brining comfort to someone but it doesn't seem to feel very alive or interactive. |
| P122 | 2 | no useful |
| P123 | 5 | I can always do with more pillows but having a little pad that talks to me seems kind of strange. |
| P124 | 1 | i don't see the use of it, buy a cat  if you lost your cat, that’s part of the life cycle, embrace it. that what actually makes us "Living" |
| P125 | 2 | I think this is because robots can’t replace dogs in any way. I feel like having this type of robot around kids as it doesn’t seem very safe. |
| P126 | 1 | I do not care for it |
| P127 | 5 | It would be good to have some form of comfort-the heartbeat for example but it doesn’t have the capacity to love you like a real animal would |
| P128 | 1 | Wrong, just so wrong in every way |
| P129 | 4 | This one is fluffy. But still a bunch of wires and circuits. |
| P130 | 4 | Looks like it might make a decent toy for my cats. |
| P131 | 1 | very odd that it doesn't have a face |
| P132 | 1 | Seems silly, a toy for a child |
| P133 | 1 | Hate it again the fur creeps me out |
| P134 | 2 | Expenses - how often am I going to use it compared to using a stagnant stuffed toy which costs nothing in comparison. Additionally, I don’t find it that interesting in comparison to objects I already own that are soft and I can pet soothingly. And I have OCD which could give me intense fears of the robot blowing up or murdering me in my sleep which wouldn’t be pleasant. But if I had the money, space and was mentally stable enough otherwise then I may buy it. But that’s highly unlikely. |
| P135 | 1 | It looks creepy i don't want it. |
| P136 | 1 | Less interactive or advanced than a 25-year-old Tamagotchi |
| P137 | 2 | this thing creep out a bit just seems you got and animal and removed everything but the tail could do with at least some eyes |
| P138 | 1 | I don't like that it has no face |
| P139 | 6 | This is simply more appealing as a life like, I could see this being almost akin to a plush |
| P140 | 6 | I like the soothing aspect of this robot, it feels like it'd be a really nice cute companion. More of a comfort than a practical job-doing robot. |
| P141 | 1 | I do not like the fact it looks like only part of an animal and has fur. |
| P142 | 1 | wtf |
| P143 | 3 | It’s too creepy for me |
| P144 | 1 | Its a piece of mechanics. A machine. |
| P145 | 5 | very cute, i love how it responds to sound and i imagine the heartbeat would be quite comforting however its still pretty limited in what it can do |
| P146 | 1 | It honestly comes across a bit creepy, like decapitated cat that's been reanimated and confusingly wags its tail like a dog, only in a mechanical way. The fact it complains about loud noises and is needy makes it more of a chore to own. |
| P147 | 1 | Don’t really see the point |
| P148 | 1 | Uneasy over it just being a blob. I think the faceless idea takes away from it. What is it imitating? What does it replace? Are pets just a moving tail, in this case? |
| P149 | 2 | It's super cute and fluffy, but not very useful. It's just having a plushie that moves and is soft |
| P150 | 1 | Not for me |
| P151 | 1 | Same answer as before but I also find this robot really creepy! I really don't like that it has no facial features. |
| P152 | 1 | The fur gives it the look of an animals pelt however with only the tail being there it seems very disturbing. |
| P153 | 1 | I genuinely recoiled from the picture of this robot. Even having been around robots, I feel scared of it. The fact that it has no visible eyes means I am waiting for them to emerge. Apparent alive-ness makes it sinister. It could be plotting to take over the world. Please send it back to a sci-fi B movie where it belongs, AS THE VILLAIN. |
| P154 | 5 | It has more of a dedicated purpose for companionship. It feels designed for it |
| P155 | 1 | The point of companionship is the connection and mutual bond with another living creature. The idea of trying to mimic this with a robot is genuinely horrifying to me. Something that you can switch off, or program to your preferences and that cannot have any semblance of autonomy and comes with no moral responsibility should never be described as a companion. These are toys at best, or sociopath creation devices at worst. |
| P156 | 7 | Similar to Paro. I would like the idea of having one around, not for any particular purpose, but more like a pet I would interact with. |
| P157 | 1 | No facial expression to distinguish. Looks like an object rather than an emotional creature |
| P158 | 6 | It might help keep people in company as it resembles a pet with a nice tail. I would like to try it out and experience interactions with it. |
| P159 | 4 | It doesn’t feel relevant to my current life, needs, or routines, but it’s intriguing and charming. Because it doesn’t seem to be trying to be imitating anything else, except maybe a Tribble or furry ornamental cushion, it’s more inviting on its own terms; as a desk or pocket toy that could be subtly comforting and reassuring, with a little of the haptic white noise of a purr or heartbeat. The quirk of appearing alarmed by loud noises is interesting/creepily manipulative, to replicate the feeling of being useful and caring to it. In circumstances with no other person or pet, I would be much more open to trying it than the clumsier mechanical seal - especially if I found myself feeling anxious or adrift. (And if given the choice of trying this or prescription drugs, I would definitely prefer to try this first.) |
| P160 | 1 | same reason of before |
| P161 | 4 | As with the previous examples, it's cute and it's "real" in that it is a physical object prompting real responses, but it's a programmable thing that can neither move nor make independent demands of its own. Also, like the seal and for reasons that I find difficult to articulate, it makes me incredibly sad. Not just downhearted: actual tearing up. I can't say why with any certainty, but at a guess, the primary emotional response appears to be pity. I find myself feeling so sorry for an inanimate object that, for all it mimics life, cannot experience anything. It's so cute and so pathetic. |
| P162 | 2 | This one feels slightly less awful than the previous robot - it's hard to put my finger on it. I think the interactivity, and general pleasant demeanour of it helps. I like that it doesn't have eyes (that I could see), something about that makes it more palatable as a comforting robot, rather than an imitation animal |
| P163 | 3 | Would rather have a real pet with actual feelings and emotions. Anticipate poor responses from general public to carrying a robotic-tailed pillow around. |
| P164 | 6 | The heartbeat and moving tail can give of the feeling of having a real pet, but it is a weird concept. Could be good for people who have recently lost a pet. |

**Rate how receptive you are to Cosmo as a potential household companion from 1 – 7. Why did you give this rating?**

| **PID** | **R** | **Cosmo Rationale** |
| --- | --- | --- |
| P1 | 1 | still not real! |
| P2 | 4 | Knowing Cozmo, I feel like I would grow bored of it quite quickly once I have played with it a few times. |
| P3 | 4 | Just looks too mechanical with no useful functions for everyday life |
| P4 | 4 | I just don’t know if this is a companion in terms of this. |
| P5 | 5 | It can give some proper company. |
| P6 | 6 | Seems like a nice mixture between responses already being in place and personalisability. It can move by itself and is less human-like. I could see this as a companion at home |
| P7 | 5 | I think as it has the ability to react and interact with certain things, it would feel somewhat receptive. However, I think this feeling may not last long as it gave the impression that though it is receptive/interactive, it would become stale/samey after a while. |
| P8 | 1 | Looks like a kids toy, I don't have kids |
| P9 | 3 | I don't like the idea of having a wandering camera in my home, though it might be useful to help explore my under-cabinet space to see how mice are getting into my kitchen. He seems kind of devious and creepy, honestly. |
| P10 | 5 | Similar to the previous robot, I think it comes across more as a toy - although the expressions improve its companion-ability. |
| P11 | 1 | Seriously if you need some stimulation do a jigsaw, read a book or buy some lego. It seems to serve no learning purpose. It's just something to relieve boredom. |
| P12 | 6 | I like it, it's cute and behaving in a clever way. I also like its mini size, easy to carry. |
| P13 | 2 | It’s doing more stuff, but when you get right down to it, not a lot beyond some clever anthropomorphic cartoon reactions to simple situations. Could be a fun toy, but probably a bit delicate for full on interactive imagination? |
| P14 | 4 | I could imagine giving this as a toy to a child and them being entertained for a while. It's not something I would want in my own home though. |
| P15 | 6 | I think actually the fact that I could control the robot makes me less receptive. I was charmed by him in the video up until they started explaining the control /programming aspect, which I think makes him feel less alive. I wouldn't want to be able to author a pet, and if I did that sort of thing I feel it would make them feel much more artificial and diminish any connection I could have with them |
| P16 | 6 | It's very charming, and not trying to emulate a real creature like the previous robot. It's clearly a 'robot'. |
| P17 | 6 | Very appealing as a play companion and for fun interactions |
| P18 | 5 | Cosmo seems like an enjoyable toy, but I'm not sure I would think of it it as an ideal robot companion because I think our interactions would be quiet superficial. |
| P19 | 2 | Another toy. Good fun, but not what I need. |
| P20 | 1 | It’s a glorified toy: no coincidence it’s shown with a child!. |
| P21 | 6 | Cozmo is a useful tool to develop an understanding of how to instruct and interact, which is useful for children and for adults, particularly those who find 1:1 interaction with another human difficult. |
| P22 | 4 | Interesting tech but I’m not sure if I want one |
| P23 | 3 | It is mechanical looking and cannot be stroked. It looks good for children but I do not have children in my house so it would probably be useless. |
| P24 | 1 | Short term fun. No long term benefits. |
| P25 | 7 | Seems like a fun toy, I like the facial expression and creative ways you can interact with it |
| P26 | 4 | I can absolutely see how it may be fun, though not sure I'd personally be interested. |
| P27 | 7 | It’s cute and reminds me of the film Wall-e. It’s little and harmless; seems like a fun toy to play around with. |
| P28 | 2 | Maybe it is the video itself but this has too much "interactive toy for kids" energy more like a Furby, etc, rather than a companion. Trying to show itself as being more intelligent (in terms of behaviour) than it is, but then you'd quickly run into things that obviously make it artificial when it repeats or makes mistakes in a jarring kind of way? I do think it's cool though generally! The animations and movements are nice. |
| P29 | 1 | If this actually works as shown it looks like an interesting toy and educational tool. I am extremely sceptical that it does though and I don't think it would have any use as a household companion. |
| P30 | 1 | Not of any interest for me at all, but think that it could be useful for younger children to learn |
| P31 | 3 | I don't think it's very cute, it just looks like a robot with a fake face |
| P32 | 3 | It is no more than a toy cannot see how it could become a companion |
| P33 | 3 | Seems like an interesting kid's toy. I'm not interested though. |
| P34 | 7 | seems like an awesome robot to have |
| P35 | 4 | Very cute, but I don't think I need him. |
| P36 | 6 | Very cute! |
| P37 | 1 | I prefer a living, fluffy being as a pet. |
| P38 | 5 | Excellent mimicry of emotions and expressiveness. Cute without emulating an animal. Recognizes faces which allows more actual interaction. |
| P39 | 7 | Well, this robot seems just as nice as the other others, but it seems like there’s more of a focus on the ability to program what he does. If this robot has self-determination, the video didn’t do a good job of highlighting it. |
| P40 | 5 | its a fun toy, but not a companion |
| P41 | 1 | "More than a toy!" Yes. A privacy and security nightmare. Also: All the things I've said before apply, too. For this to have any staying power and not land in its box in the attic or in the bin, it would have to show new, emergent behaviours. It would have to be unpredictable, do things it wasn't programmed to do, be even a potential danger (at least on a small scale, like a budgie could pick at you, or a cat could scratch you). Without that, it's just an over-prized remote-controlled toy with a few pre-programmed behavioural features. Isn't it? |
| P42 | 3 | Looks like a good toy for smaller children to make memories and become nostalgic when they grow up. I mean it would be good and enjoyable for having this robot for a week or month and then the excitement would fade off eventually. |
| P43 | 6 | The interaction with the human looks a lot more natural, that the girls actually smiled, because of the movements. This could be something I want in a companion. Though it still looks like a toy |
| P44 | 1 | Most annoying one if the three. Again I didn't see it do anything useful or interesting |
| P45 | 3 | It seems more targeted toward kids, so I'm not sure it would suit me overly well. I'm more so looking for either something useful or something that could fill the place of a pet or romantic partner. |
| P46 | 7 | I would like to get one also for playing fun with because Cozmo is very cut little friend, he even can recognize the dog! He is very smart little boy! |
| P47 | 1 | It's obviously a robot. Why would I need or want a robot as companion? That's not what companionship means to me. |
| P48 | 4 | I'm personally not looking for a household companion to play games which is all this robot seems to do. I think it would be good for children. |
| P49 | 4 | As before |
| P50 | 7 | Cosmo seems to be the most 'lifelike' so far, with it's personality and interaction with the world (albeit preprogrammed) I could see interacting with Cosmo being framed in a social way, like a conversation rather than a one-way talk towards an inanimate robot |
| P51 | 4 | because of its size |
| P52 | 5 | It looks quite fun and expressive through its movements and facial animations, so could be entertaining or endearing to have around at times. |
| P53 | 4 | it is a robot, so it is something artificial. not sure what to think about it |
| P54 | 4 | Pleasant expressive eyes |
| P55 | 5 | Looks like fun and a little cute at the same time, if you have time to play or kids in the house |
| P56 | 5 | I do not think the robot is anything other than a pre-programmed (inc. machine-learned) robot. It looks appealing and fun to play with, but I would not trust it with any task it has not been programmed to handle. |
| P57 | 4 | it is more for kids as it is more play-focused and therefore i would consider it for children but not for myself |
| P58 | 3 | it looks weird |
| P59 | 7 | Cozmo has an emotion as facial expression, so you can see it on display screen. I think it is very cool features and children or all ages allow to be playing with him. |
| P60 | 6 | From the video, it seems like this |
| P61 | 5 | cute |
| P62 | 5 | It's ok. |
| P63 | 7 | It look like it can be a good friend to me |
| P64 | 3 | i don’t feel it’s necessary |
| P65 | 7 | Looks very cute, I want one as well |
| P67 | 6 | Because it's so unique and beautiful! |
| P68 | 7 | Cute useful and clearly robot |
| P69 | 6 | Smart and listen to order/ideas |
| P70 | 4 | Don't sure |
| P71 | 6 | I think it would be fun seeing it run around the house. |
| P72 | 7 | It looks like t has potential to help in household |
| P73 | 4 | I would be unsure if I were to like his company or want to tear it to shreds for being too exhausting to deal with |
| P74 | 7 | look fun to play |
| P75 | 5 | I like things with wheels |
| P76 | 7 | Good |
| P77 | 6 | It feels more like a companion, since it can also move around and do things on its own. |
| P78 | 5 | My friend brought it and from what he shows, I like it |
| P79 | 5 | A good toy to play with and kill some time |
| P80 | 6 | Look cools and have many features |
| P81 | 4 | Unsure |
| P82 | 7 | He is a robot but cartoon like and this is what a kid will love to play with |
| P83 | 7 | this robot looks pretty cool |
| P84 | 6 | Looks like a fun robot for playing around with in the free time |
| P85 | 6 | I think this is more fun around like I could keep on my desk. It feel like I know it is a robot and it is not trying to be a pet which make it more pleasant to be around with. |
| P86 | 6 | this robot is good overall, it can be good for kids while helping them do their tasks and it also has a lot of function implemented into it |
| P87 | 4 | Looks sort of fun but a bit limited in range of interactions. |
| P88 | 4 | I am not sure I am ready for this type of interaction, however, I could see the benefits for children and perhaps the elderly. |
| P89 | 4 | Never experienced anything like that. |
| P90 | 4 | I don't really have any strong feelings about it |
| P91 | 6 | I want 10 |
| P92 | 7 | Have had very similar toys in the home in the past. Can challenge a lot of skills & encourage learning |
| P93 | 2 | It looks like a fun toy, nothing more |
| P94 | 1 | It is very stereotypically "robot-like".. with the annoying noises, loud movement, etc. To me, a good 90% of having a pet comes from the personal physical interaction, their smell, how fluffy they are, petting them, hugging them. This is a piece of plastic, no different to any other plastic, I would rather have a plushie. |
| P95 | 1 | same as Roomba and robot number 2, Cosmo just not a pet |
| P96 | 6 | I love the little mechanical guy, he’s a funky little being |
| P97 | 6 | he's so cute. I'd love to take him into university and study with him on my desk |
| P98 | 4 | I think the cute face and interactions with the user makes you feel happy and want to have one but to me I feel that way but I see it as a toy and would use it once and put it away |
| P99 | 6 | good for the kids, a novelty |
| P100 | 6 | Not a pet substitute for me but looks a dream toy, particularly for a child. Looks a lot of fun! |
| P101 | 3 | its not as cute as the other robot, but it seems more interactive with the games it can play with kids so it can help to stimulate them |
| P102 | 4 | As it such a small robot, I believe it is more an interactive toy to potentially educate children and does not feel like a household companion. |
| P103 | 6 | The robot seems like a cool toy to play around with and can be useful for kids to learn some basic coding skills with. |
| P104 | 6 | My favourite one, I think probably because of the cute, immediately readable facial expressions and because he's not trying to imitate a specific animal or a human - oddly enough that makes him seem more lifelike, maybe because he doesn't immediately invite the same comparisons to a living creature. The advert makes him seem curious and playful, like he has a personality. He reminds me of Wall-E. I still don't know if I would ever opt for Cozmo over a living pet (he doesn't hold a candle to Gizmo) but he is endearing and entertaining to watch. |
| P105 | 6 | Would be fun to see what this companion can do |
| P106 | 7 | I have 2 |
| P107 | 1 | The sounds coming from it I didn’t enjoy  Think it would scare my cat |
| P108 | 4 | wouldn’t get but don’t hate |
| P109 | 4 | I cannot see a use for this robot in my day to day life |
| P110 | 7 | Seems so cool!! |
| P111 | 7 | Can interact in an emotive and can initiate action |
| P112 | 1 | My kids. |
| P113 | 1 | - |
| P114 | 1 | it's a toy, not a companion. |
| P115 | 5 | If having a human or pet as a companion wasn't an option, this would be an alternative, but it can't compare to a living, breathing companion |
| P116 | 2 | I have no need of such companionship. |
| P117 | 3 | Hmm maybe as a child it would be fun but I don't think I'd want one now. Unless it plays with my pet who might feel alone |
| P118 | 6 | Robots are very interesting, and displaying simulated emotions could make them good companions. Yet you cant avoid errors, and a robot that could ever potentially get angry or violent due to technical error is a real danger I am concerned with. |
| P119 | 7 | The robot's personality is very friendly and cute. It would be a pleasant household. Although I personally think to have such a personality, I don't think it's necessary that the robot should have emotions or be conscious at a human level. |
| P120 | 4 | I think while Cozmo while charming and genuinely full of personality in its expressions and design I would struggle on having them as a household companion due to, from what the video tells me that it uses a camera for it's sight and that is something I wouldn't personally want living in my own household |
| P121 | 5 | It seems like more of a fun toy, perhaps useful for very specific times. Though I don't see it being useful or desirable daily. |
| P122 | 7 | its very useful and funny |
| P123 | 6 | Seem's like a good tool to introduce kids into coding. |
| P124 | 7 | As person who doesn't like pets, I would still like to have this my home as a toy to play with. maybe making it to do simple tasks like passing the remote or drink from kitchen to sofa. |
| P125 | 1 | I do not like this robot as it does not give off the energy as a pet would at all. |
| P126 | 2 | I don't care for robots but this one is mildly cute |
| P127 | 7 | It has a cuter appearance than the other robot, making it less intimidating I suppose. |
| P128 | 3 | Fun and interactive |
| P129 | 4 | It's a nice wee robot toy. I don't need my emotional needs met by a toy |
| P130 | 1 | It's a child's toy. Not something I'm in the market for. It also looks like something a kid would play with for a day or two then relegate it to a shelf. |
| P131 | 7 | this is like a Tamagotchi, not trying to be anything but a bit of fun, love it |
| P132 | 4 | Might entertain children |
| P133 | 1 | Seems more decoration geared than anything else |
| P134 | 2 | I have no use for it. My OCD may trick me into believing it would kill me in various ways. It looks cute but that doesn’t mean I need it or want it. |
| P135 | 5 | Could be fun toy to give to my younger sibling. |
| P136 | 1 | Good kids’ teaching aid/toy, otherwise irrelevant |
| P137 | 4 | felt to small and more of toy |
| P138 | 5 | It has cute expressions |
| P139 | 4 | I'm simply no more or less likely to invest in a mechanical companion vs an organic one |
| P140 | 7 | I love these guys! I've seen them a few times, such a lovely cute wee companion to have. I'd love one of these guys around the house, so many way to interact with them and they really seem like they're learning as you get to know them. |
| P141 | 5 | It is not humanoid, more like an animal/pet |
| P142 | 2 | i don't need one and i am afraid for my private data |
| P143 | 6 | Look fun and interesting |
| P144 | 1 | I am just not into tech. And this is a piece of tech. |
| P145 | 5 | Very lively, feels like theirs actually something to interact with and i imagine kids would love it, however, once the gimmick wears off it would be pretty uninteresting |
| P146 | 5 | This isn't really a companion for an adult. It's more of an educational and fun toy. Again, I would be happy to have this in my house, but mostly for the education and entertainment of my children. |
| P147 | 2 | I wouldn’t want one, but might have considered it as a learning tool (coding)/plaything for my don when he was younger (18 now) |
| P148 | 4 | It's cool, sure. It has expressions, but I don't think that means it has emotions. These are artificial, and they're programmed in. |
| P149 | 6 | As a toy, it's very cute and funny. And It seems to not just give entertainment but also teach |
| P150 | 1 | It’s a toy |
| P151 | 1 | I think robots are built to be functional but that their use as a companion has a very limited scope. |
| P152 | 5 | the small stature and facial expressions lends to the cuteness of this robot that somehow makes it seem more acceptable as a household companion. |
| P153 | 4 | This robot seems to be more actively interacting with an responding to its environment than Paro and NAO. Self-locomotion makes a very big difference. Something about the smallness also makes it seem like it might be sneaky, mischievous, and might cause trouble. It's more appealing by being obviously a machine instead of imitating an animal or humanoid. |
| P154 | 4 | Feels still to robotic but its more friendly looking |
| P155 | 1 | There is no possible robot design that would be a convincing substitute for genuine companionship provided by actual pets. |
| P156 | 7 | I would enjoy playing with this Robot with my son and cats. Similarly, would also be fun to use in outreach activities. |
| P157 | 7 | Friendly design, very interactive, fun and creative |
| P158 | 6 | That's nice! You can empathise with it, as you can see through its "eyes." Its interactions are lively and kind. However, the lookalike is not as close to a human as the previous "pet-shaped " robot. |
| P159 | 1 | It’s not designed for me. I can see how it could intensify a child’s curiosity and exploration of their world inside a home. But it would be more annoying than companionable for me. |
| P160 | 7 | I love it ca also be very nice for the kids to have it |
| P161 | 4 | It's cute and interactive, but it feigns friendliness, liveliness, etc (it also can't be "dead" as that implies being formerly alive, more accurately it is "lifeless"). I'm not sure I'd gain anything approaching companionship from a "being" that can be programmed. I think it's the level of control I have over the robot that makes me regard it as less than a companion. Being able to see behind its eyes feels more akin to possession (in the demonic sense), and I can no more update the programming/behaviour of a houseplant than a companion. It might bring me joy, but that would be entirely in my own head. |
| P162 | 4 | I think this robot reminds me of Wall-E - it's cute! I still do not think I would like it as a household companion, but I would consider having one alongside my pets. It feels like more of a nice distraction to have around your working environment |
| P163 | 6 | Entertainment purposes |
| P164 | 5 | It seems like great fun for kids and adults, it is quite lifelike in some aspects as it reacts to different actions and 'has' emotions. I don't think it could be a sole companion to keep someone happy and lively as it is essentially a toy. I think human interaction is important for humanity to thrive, but not everyone lives with another person. It can be a good companion if there is no option for pets or plants. Although it is just a 'toy' it wouldn't serve the same amount of companionship as a pet or another human. |

**Rate how receptive you are to Roomba as a potential household companion from 1 – 7. Why did you give this rating?**

| **PID** | **R4** | **Roomba Rationale** |
| --- | --- | --- |
| P1 | 1 | just electronics |
| P2 | 1 | Roomba's can't do social interactions. It seems like it would be very boring as a pet. |
| P3 | 5 | It saves me some vacuuming time! helpful. |
| P4 | 7 | I like the Roomba it cleans for me. |
| P5 | 7 | This is very helpful for cleaning. |
| P6 | 5 | I would love a Roomba... but not necessarily as a companion, but as a little worker doing something for me I very much dislike doing myself. It seems a bit too focused on a single purpose for a companion |
| P7 | 1 | It doesn't really respond to any stimuli except for when it bumps into things, so I don't think it would work well as a household companion. |
| P8 | 5 | Saves time having to hoover |
| P9 | 5 | Roomba seems like it has a task to do and then it does it. It's probably more reliable than some of the people I work with. |
| P10 | 1 | Roomba feels extremely basic, more like a tool than anything. |
| P11 | 5 | I was thinking this would be a great replacement for hoovering. My only concern was towards the end of the commercial it said that you had to control it. Initially the advert gave the impression that it was detecting where to go and vacuum. |
| P12 | 6 | Yeah I like it and sure I'll buy something like this to help me clean my room. It's nothing about companion though. |
| P13 | 3 | I understand they are better than they were, but until they are fantastic, and i know my dogs will love them, Roombas are not something I’m interested in. |
| P14 | 5 | It's a vacuum cleaner. If I thought it would be useful I would be fine buying one, but it's a tool, not a 'companion'. |
| P15 | 5 | Firstly, it would provide me a tangible benefit beyond any companionship, which makes me more inclined towards it. But I think also it would be quite nice to have it bumbling around the house, it would add a degree of life to the environment that I think would be good. I could imagine if I had one it would end up with a nickname and googly eyes |
| P16 | 3 | I don't see the point in robot vacuums, I think they might be a waste of resources. |
| P17 | 5 | competent (well designed and programmed) mechanical device but not really a companion! |
| P18 | 6 | I'm definitely receptive to it is a functional companion in a 'robot servant' sort of way, but I'm not sure as a true companion as it doesn't seem very socially interactive. |
| P19 | 5 | A useful tool with significant programming to allow it to function in an environment that changes. Happy to have it about the place, but doubt my preferred living arrangements would suit the robot. Would I adapt my space for it? Doubtful. |
| P20 | 4 | I like the idea of a labour saving device, but experience tells me it will break down, get clogged up, and eventually either break or become obsolete |
| P21 | 6 | Roomba cleans, which is something I would rather not do! In addition to the cleaning function it would also entertain the cats. |
| P22 | 1 | It’s a low functioning device |
| P23 | 2 | It looks like a machine and would be useless to me |
| P24 | 4 | Like Daleks, I suspect it might not handle stairs too well. |
| P25 | 5 | It’s just a hoover, useful but hardly a companion. |
| P26 | 6 | I already own one! |
| P27 | 6 | I wouldn’t call it a companion but it’s useful and I’d definitely have one. |
| P28 | 4 | It feels insane to feel this way following Anki but... I do feel like I could end up feeling a bit attached to a Roomba? It would reliably do one thing (vacuuming) well and I can see myself putting stickers or something on it to make it feel more friendly. |
| P29 | 5 | Useful and therefore a good household companion on some level. Amusingly stupid in a way that is similar to pets sometimes with an extremely good humour to investment ratio |
| P30 | 4 | Would be nice if I had a bigger place |
| P31 | 6 | Roomba isn't trying to pretend to be something it isn't. It's not pretending to have emotions and pretending to be alive; it's a mechanical robot, and that can be cute by itself. |
| P32 | 3 | I already have a similar product and have real difficulty with calling it a companion it is no more than a tool to hoover flooring / carpets |
| P33 | 5 | Could be useful in certain circumstances. Could see myself using one if I had a suitable home. |
| P34 | 6 | purpose built robot. Seems like it can do it work just fine. |
| P35 | 7 | If I had a roomba I would call her Ivanka and cherish her. |
| P36 | 6 | Serves a simple and easy purpose |
| P37 | 6 | Practical for the household. |
| P38 | 1 | It's a machine. It accomplishes tasks, but does not react intelligently to it's environment. All responses are algorithmic. |
| P39 | 7 | I’ve had a roomba in the past, and I loved it. Wouldn’t mind having one again. |
| P40 | 6 | Its a useful tool, not a thinking companion |
| P41 | 1 | Eh, waste of energy and space. Another thing in the house that can break, has to be maintained and fed (with electricity) and probably gets under your feet constantly. All the other points still apply. But, honestly, on a purely usefulness-based scale, I would this rank second only to the toy-robot. |
| P42 | 7 | I think robots should mainly make a humans life easier. That is why we see a lot of these interactive bots and fade away with overtime meanwhile automotive robots or surgical robots are growing and their potential is being fulfilled. The Roomba is exactly the latter one for which I am all in for. |
| P43 | 4 | I think it could have the job done, but there's no significant interaction with the human |
| P44 | 4 | I'd probably get a robot vacuum if my flat didn't have thresholds between all the rooms that present insurmountable barriers. |
| P45 | 6 | It seems like a very nice thing to have around the house, and it would definitely be helpful. With that said, I worry a little bit that I would trip over it, and configuring it seems like it might be a little bit difficult, since I have quite a lot of stairs in my house. |
| P46 | 7 | This robot is very helpful for cleaning and be able to help us as responsibility to our house. It is very beneficial for the person who is lovely to clean a house with assistant. He is also very smart as charging on his own by knowing his own place to be. |
| P47 | 1 | This is a machine helping with tasks - very welcome on this level. But this is not a companion. I do not socialise with household machines |
| P48 | 7 | I have a Roomba and I sometimes speak to it as if it can hear and understand me! |
| P49 | 4 | Very ambivalent about it - not against it, but don't really want one either. |
| P50 | 4 | This seems more of a functional robot, and there isn't anything to form a bond with |
| P51 | 5 | For its usefulness |
| P52 | 1 | It's more of a tool with a purpose (cleaning) rather than a companion, there aren't enough social or emotional cues, and it's all hard, dark plastic and metal. |
| P53 | 6 | i would have because he has an utility, i don't want to interact with him. i just need something that makes things for me |
| P54 | 1 | Appliance not companion. |
| P55 | 6 | Such a practical thing to have in the house! Would save me time |
| P56 | 6 | Roombas are useful. |
| P57 | 5 | It is very useful as it can take over a repetitive and unpleasant household task that I don’t enjoy doing. However, one major drawback is that one has to potentially reconsider the furniture and set up of the home, which would be a big constraint. |
| P58 | 6 | it help me clean the house |
| P59 | 7 | Roomba bot is the cleaning machine to help you clean the rooms and he has lots of responsibility. |
| P60 | 1 | I rate from my feeling and idea |
| P61 | 7 | good for cleaning |
| P62 | 5 | Very convenient! |
| P63 | 5 | It can help me clean the floor |
| P64 | 7 | its a machine. i don’t need a companion |
| P65 | 7 | My mom also have this at home so I definitely know its usefulness |
| P67 | 4 | Can be better |
| P68 | 4 | i see as a tool |
| P69 | 2 | It just do what its design to do |
| P70 | 5 | My house has this kind of cleaner robot before. |
| P71 | 4 | This would definitely help me clean my room. |
| P72 | 4 | Robot works like a simple mechanical things that does not looks like organic being or have any emotions |
| P73 | 5 | I would want this to clean my house and do just that. |
| P74 | 6 | good to have it for house cleaner |
| P75 | 7 | because i like my house clean |
| P76 | 4 | It is just a Roomba. |
| P77 | 2 | It is more like a tool or a machine than a companion. It is like a normal vacuum cleaner, but just automated. |
| P78 | 2 | because voombot goes woom, but honestly it just exist and not sentient. |
| P79 | 6 | Good for cleaning the floor and doesn't look intimidating. Feel more like a small pet |
| P80 | 7 | Useful and don't have mush risk |
| P81 | 6 | i like the design |
| P82 | 5 | It a robot that design for cleaning purpose it does not need to have that much of interaction and friendly look to it. |
| P83 | 6 | Its a robot that does its function, which is cleaning dusts in your house. Every household should have it |
| P84 | 3 | Not really a companion, but more like a tool |
| P85 | 7 | This one has a specific purpose for us, to make a daily live easier. I think this rating is good because I would definitely have one. It also seems like it is built for something not trying to be something which is I prefer on a robot. |
| P86 | 7 | this is a useful robot that a household can actually use in their house. It is a useful item, its not like the other kind of a robot but it is certainly useful and smart enough to clean your house |
| P87 | 7 | I have one. Does a really good job. |
| P88 | 5 | As a functional tool yes, I could see the benefits |
| P89 | 4 | Not a companion but maybe a useful tool. |
| P90 | 5 | It has a defined household use |
| P91 | 5 | It's not particularly alive, but it'd be handy to have in the house due to my chronic pain |
| P92 | 7 | Have one. Simply a mechanical tool that does a physical job i am unable to do. |
| P93 | 5 | I would value its ability to complete a necessary task for me. |
| P94 | 4 | This is more of a functional/household robot, you aren't meant to build a connection with a roomba. It is there to accompany you as a service. |
| P95 | 1 | it's just a cleaning tool, it does not engage with it's environment or self as I would want from a pet |
| P96 | 4 | He has a function and doesn’t try to be a pet or a human, similarly to the previous little cube guy |
| P97 | 4 | I dislike the idea of a companion having a job. |
| P98 | 5 | It doesn’t have a face and trying to be like an animal or human and it looks like the job it was designed to do |
| P99 | 4 | i would rather just hoover myself and know it done right, plus my dogs would see it as a toy |
| P100 | 5 | I don't see this as a pet replacement at all but as an automated useful household tool it could be a nice idea. Not sure how it would deal with stairs though! |
| P101 | 7 | it isn’t seen as a pet substitute, but as a cleaning helper |
| P102 | 4 | As a companion it is good for doing tasks, however it's not something that I would create a bond with |
| P103 | 6 | Unlike the previous robots that were more designed as kids toys, the roomba has practical use for cleaning the house and if it functions as intended, it could be very useful for doing tasks that would otherwise require your time. |
| P104 | 2 | The pervasiveness of memes surrounding Roombas has habituated me slightly to the idea of the roomba as a living creature, and the rapidly wagging brush does remind me somewhat of a dog's tail, but ultimately I view it more as a useful tool than as a companion. |
| P105 | 6 | Wouldn’t be intrusive and would help me with chores. |
| P106 | 5 | I have one but its not a friend |
| P107 | 1 | Can’t hold enough dust and dirt and needs to be emptied often . |
| P108 | 6 | seems useful |
| P109 | 7 | I have a vacuum robot. As long as it is programmed correctly they are useful. |
| P110 | 7 | Hoovers for me and avoids things |
| P111 | 1 | It can't express, or listen or another way interact bidirectionally with a person |
| P112 | 1 | My children would not be a fan. |
| P113 | 1 | That is a vacuum cleaner be fr |
| P114 | 1 | Same as previously, this isn't a companion, it's an appliance. and I don't like the idea of robot appliances. |
| P115 | 6 | It appeals to me innate laziness and hatred of housework, but it's not exactly companion-material |
| P116 | 7 | I'd consider one for the practical purposes. |
| P117 | 5 | I'd say I would choose it because it vacuums the floor! But if it was a fluffy vacuum I'd 100% take it |
| P118 | 7 | It is functional, it doesn't simulate things like emotion with room for catastrophic error. It does a job and that’s it |
| P119 | 7 | It's useful and doing the job I need. |
| P120 | 5 | I am more positive than negative with it being a household companion as I can see enjoying its presence while it does its task but its not something I would be as intimate with |
| P121 | 4 | It's useful and helps with a very specific chore, aside from that it doesn't have much use. |
| P122 | 7 | very useful |
| P123 | 6 | Makes me vacuum less and anything that helps keep my house clean with minimal work from is is nice. |
| P124 | 7 | Helpful to make life easier |
| P125 | 7 | I like this robot is great for people that don’t have enough time and have a busy schedule. I also think it’s good for people who have businesses. |
| P126 | 6 | It's decent at cleaning, saves the job hoovering |
| P127 | 5 | It’s helpful, very artificial and I suppose I wouldn’t really see it as a robot. Just a household appliance |
| P128 | 1 | It is a hoover not a companion |
| P129 | 7 | The robot cleans up my messes. Great. Much better than my dog who I often have to vacuum up after. Unfortunately my dog is anti-robots and will try to eat it.  I think she just doesn't like wheels though |
| P130 | 7 | I think i have this model of roomba. It generally works well until the battery fails |
| P131 | 7 | its a useful tool depending on the brand |
| P132 | 6 | Provides useful service |
| P133 | 6 | Seems useful but doesn’t seem that much of a ‘alive’ robot |
| P134 | 4 | I’m more likely to buy this one as it has a purpose I would use it for. However - expenses, OCD, is it not just easier to keep the vacuum we have!. |
| P135 | 3 | I like cleaning myself to make sure everything is clean. |
| P136 | 6 | I have one. Limited but useful. Ratings of 3 indicate that the question is irrelevant |
| P137 | 3 | its a smart hoover not really a companion |
| P138 | 5 | I had a similar robot which I liked |
| P139 | 5 | It seems to have solid utility but lacks any real quality's of a companion. That said if I did obtain one I would likely add eyes, or similar humanising features. |
| P140 | 7 | it's a cute wee robot, knows how to do it's job very well and would be nice having it wandering around. It doesn't speak but I'd probably speak to it anyway, like say "Hi Roomba" when I saw it going about it's business. |
| P141 | 1 | It has a specific use I do not need. |
| P142 | 7 | so useful |
| P143 | 7 | I don’t like to clean. |
| P144 | 1 | Its a plastic object. |
| P145 | 7 | Actually useful, designed to do one thing very well and it would be pretty entertaining to watch |
| P146 | 2 | I own one of these, but it's not a companion, it's a tool. Hence me giving it a 2 as how receptive I am to it being a companion. To me a companion is someone (or something) the shares a mutual wish to spend time with one another, is supportive (emotionally and physically), helpful, etc. While this is a helpful robot in terms of keeping your house clean, it isn't a companion. If you were to ask me how receptive I am in owning one, I would have rated it a 7, but as a companion it terrible. |
| P147 | 1 | I don’t need one, quite happy with my vacuum |
| P148 | 6 | Useful as a companion in the sense of helping with household tasks. It has no lifelike features, though. It's not designed to be a companion in the sense of a pet. It's still useful to have, though. |
| P149 | 4 | I don't see it as a companion but as a useful cleaning machine |
| P150 | 1 | I’d prefer a proper vacuum |
| P151 | 1 | Same as previous answer. |
| P152 | 5 | I feel like a lot of these are already integrated into some households, it feels somewhat okay but the nature of letting a robot do these household jobs seems surreal. |
| P153 | 4 | I feel more liking and confidence about this robot because it has one job, and mostly does that competently. It has NO pretence of having a personality or being cute. No one needs to like it, or wonder if it is kind, or whatever--the same way you don't worry about an ordinary vacuum. Unlike the other robots in this lineup, it has a definable purpose and that is very positive. |
| P154 | 3 | It has a task of cleaning already. Not very suitable for companionship |
| P155 | 4 | Using robots to complete simple, clearly defined tasks is perfectly acceptable. The question asks if I would accept it as a "household companion" - if this means would I have it in my house, then yes. If this means would I consider it as a companion, then no. |
| P156 | 7 | Mostly for the value of what its main function would be, but I'd be willing to use it for other tasks it could be capable of performing, similarly to my comments on Nao i.e. if it had voice recognition and could do things like set reminders, check if I have any new email, when my next meeting is etc. |
| P157 | 7 | Clever design, completes household task with little intervention |
| P158 | 4 | Roomba can't be considered an actual "companion." It lacks a pet-like shape to be perceived as such. In addition, its task is simple and mechanical, which does not entail a specific companionship quality. |
| P159 | 2 | It might be functionally helpful, in very limited areas of the house, but doesn’t show any interpersonal qualities or character traits that feel like companionship |
| P160 | 7 | this is very nice to have because it can help |
| P161 | 2 | As with the humanoid robot, this is a servant, not a companion. It cannot be either responsible or irresponsible, knowledgeable or ignorant, incompetent or competent, it lacks the capacity for all of these things. One might as well pack bond with a dustbin. |
| P162 | 2 | I am not averse to having this robot in my house, as it is performing a specific task that is labour intensive. I think that I would also grow to enjoy seeing it going about it's business.   I've seen people online putting googly eyes on their roombas, I would probably do this also to lighten up it's presence.   But again, it is not a companion to me, it's a tool to perform a task |
| P163 | 5 | Because it’s not directly imitating a pet, it’s easier to detach any expectations of emotion, and just treat it as a robot. |
| P164 | 2 | This robot is not interactive, it is a cleaning tool. It doesn't have companionship qualities as it serves no reactions or emotional responses. |
