# Supplementary material for: “Cute Companion” or “Awful Thing”: Understanding Individual Differences in Attitudes to Social Robots as Pets
Source: Int J Soc Robot. 2026 Nov 2;18(8):115. doi: 10.1007/s12369-026-01453-4 (PMC13727923; doi:10.1007/s12369-026-01453-4)
Supplement: Supplementary file 3 [file 12369_2026_1453_MOESM3_ESM.zip › Data IJSR/Qualitative/AZRAM Survey Qualitative Pets v Robots.docx]

**Considering the relationship you have or had with this pet, please summarise experiences that you feel are or were important or beneficial for you.**

P1 it taught me responsibility.

P2 Cuddling it, being greeted by it, playing with.

P4 It is important to me and part of my morality to allow my pet (dog, Zack) to have an autonomous life within my care. It is important to me that he is allowed to explore and build up competencies. Walks with my pets are important moments for us.

P6 I had a dog (family dog) and the unconditional love was just so valuable. She would always be happy to see me, play with me and insist on it, really giving me the feeling she wanted to spend time with me. It could get annoying when I did not have time, but I still valued it a lot. She also was intelligent and treated everyone in a different way, knowing exactly what was ok for whom, so she made me feel seen and accepted in a way that I hadn't gotten from humans as much.

P7 Evening cuddles. They are a nice way to wind down at the end of the day, especially if it's been particularly difficult.

P8 Taking him home from the shelter and getting head boops from my cat

P9 My fish were a constant in my life growing up--I got them early in elementary school and the last one died the day before I moved away to university. When I was really stressed, I would just watch them swim around their little tank and it was very stress relieving. They were just kind of there, but that was enough, I think. It was reassuring, though I was always nervous they were going to die or that we weren't taking care of the tank properly. Once one got stuck in a shell in its tank and my family spent a meticulous hour trying to break it apart to help the fish escape--all was well in the end and it was a canonical family bonding moment we still talk about nearly 20 years later.

P10 When I think negatively about my pet's health, life expectancy, quality of life, etc, I find it calming and reassuring to think about how much improved their quality of life is over if they had never been rescued. Generally, my relationship with pets is best described by this enjoyment and fulfilment in the idea that I have helped animals live a happier life.

P11 My cat is very independent. Sometimes she can drive me up the wall but most of the time she makes me laugh. She's the best alarm clock in the morning as she's a stomach on legs and wakes me up by sitting at the side of my bed meowing and touching me with her paw. Most of the time she just sits on her cushion but she goes up to the radiator when she wants the heating on. There's only me and one other person that lets her pick her up but she's not high maintenance. She just pleases herself which is mostly sleeping and eating. My dog is the most affectionate dog. She's a bit of a velcro dog in that she won't leave my side sometimes. She got me over the death of my father. I know that when I get home she'll be there waiting for me and gives me joy when I open the door and we have are both so pleased to see each other. She calms me down and gives me a boost when I'm feeling down. She also helps in that I get exercise walking her which is beneficial for my physical health and mental health. Plus it makes me get out the house when I want to stay in and avoid the world. Then there's the social interaction with other people walking dogs. I'm pretty shy but other people with dogs have that knowing look and sometimes have a chat when the dogs show an interest in each other.

P13 Caring for our dogs brings joy, laughter and comfort every day. It also makes us take exercise outside. They are effortlessly affectionate and good tempered - and sometimes a bit pushy around walk and/or meal times. It’s difficult to be self-centered when you have a loving, un-critical being relying on you

P14 Lap time, quiet time, companionship, affection

P15 I don't have a pet of my own, but my family has a dog. I'm not especially close with him (I'm not much of a dog person), but do enjoy seeing him when I go home for a visit. We don't interact with each other much, but I think that his presence in the house adds some more life to it, and makes it feel more like a home, which feels important. If he wasn't around, I'd notice his absence very clearly even though I don't consider us to be close.

P16 My pet actively comes towards me when I gesture to it.

P17 physical contact, signs of contentment (eg purring),

P19 Nothing to add

P20 Having my cat purring on my knee is very relaxing

P21 Unconditional love is shown at times when I feel anxious, upset or vulnerable through their eyes, their physical actions (wagging tail, purr etc). Their responsiveness to my emotions is shown by positioning themselves close to me, rubbing against me, licking/sniffing my face and by placing a paw on me. They will bring me their favourite toy as a “gift”, sit where I usually sit and purr (dogs as well as cats!) in response to me cuddling them. Some of them show me that they missed me when I have been away by withholding affection and staying away from me while others are very effusive with their affections on my return, which can be an absence of just a few minutes. If I am unwell they will want to lie next to me, giving me warmth and comfort.

P22 It was rewarding having them there

P23 Her affection (licking my ears, playing with my hair, snuggling me in bed) are very relaxing and make me feel happy and cozy.

P24 Caring for pet. Respecting its space / requirements.

P25 My rabbit often shows affection and likes to be close to me. When she gets excited and jumps around with joy I feel happy.

P28 I think it's the daily activities I find important in a grounding sort of way? Reliably I will get woken up when it's cat breakfast time. When I sit at my computer for work every day, pretty reliably a cat will eventually appear and lay down over my arms. When I sit down in the evening to relax, a cat will eventually appear and sit on top of me. It's noticeable when these are missing (e.g., cat at the vet for the day, me staying elsewhere). It's hard to think of any one important event other than the day they arrived, which feels surprisingly not notable really since the bond is slow to build I guess?

P29 1. Watching him be athletic and graceful 2. Watching him be a clumsy idiot 3. Practicing empathy, patience, and generosity in a low commitment/stakes context

P31 When we first adopted her, she was scared of everything. I was the main one to help her feel safe and comfortable in her new home with us. Ever since, we've been very close

P32 Our dog was part of the family and nothing better than getting out for long walks, keeping fit and generally enjoy my time alone with my dog

P33 It's nice to care for something and see it flourish

P34 mutually beneficial

P35 We are very close and love each other. We sleep together, we play together, and it's awesome when she shows her love.

P36 Strokes, cuddles, grooming.

P37 Every experience was important for me.

P38 When my moms dog, who used to growl at me, started to protect me from strangers.

P39 Being a cat owner, I’ve learned to respect the space it chooses between us. Sometimes he likes to sit in my lap, other times he likes to be in the other room. I still love him, he's like a son to me, but he's a living being who makes his own choices, and we love spending time together. As my wife says, we have a cat to have a new member of the family - not an accessory.

P42 My first pet was a cat. I brought her into my house because it was lost and hungry and I felt piety. At first my parents outright refused and told me to leave the cat outside but me and my siblings wanted to help this poor kitten at all cost. Cleaning litter, teaching it to stop scratching sofas and curtains, everything was a new learning experience about this kitten. The kitten became very affectionate and use to loudly purr which we used to cuddle it. Bought food for the kitten even though my parents were against it. The cat loved us and was quite aggressive to other cats, consequently it was more friendly to humans. It used to even confuse with other humans and use to rub her head against the feet of my neighbours as a sign of love. The first time when she left the house we were very cautious and use to go on the ground floor of the apartment to keep a watch, sometimes keeping it in the cage even if it wanted to play more outside. But as she grew, we allowed her to roam outside more because she felt more interested and felt a bit cruel to not allow her to go outside. My parents accepted her and also became affectionate of it as time passed. One time she did not return for 3 days (probably because she wad in heat) and we were really depressed and sad that we lost her but later she did come back home. She spent more time outside as she grew older and we got used to it. One night she did not return and later we asked the garbage collector the next morning who confirmed that he had seen a dead cat which he had disposed the previous morning. Obviously, we were really sad of losing such a great pet. It took time to recover from such a great loss. Then a cat came near our flat and put her two kittens for which we started feeding. And from then on we are feeding the cats and now one of them has 3 kittens which we are taking care of.

P43 When I felt alone my dog came to me and was my company and it made me feel relieved, cause sometimes I could get nervous for no reason, specially if I was alone.

P45 It's worth noting that this pet lives with my parents, and I usually stay in dorms in another city, so I'm not around him often. But it's nice that he's always happy to see me when I come back. I do feel a bit of a disconnect sometimes, though, like he's not as close to me as I was with pets I've had in the past.

P46 I have a pet as 2 rabbits called "Money" and "Golden", they are very support me as If I fell sad, they will come around and running to me, and they stay on my legs.

P47 I love my pet, I would be upset if she were sick or in pain, but I do not need validation from my pet. She is also not a replacement for human interaction or love for me.

P48 I felt happy if they slept on my lap, as I could believe they felt comfortable and safe with me.

P50 The first time she sat on my lap and let me stroke her. I never had a pet growing up and was nervous about having an animal in the home (having been scratched by own when younger) but the cat was very calm and showed me that not all cats are like that

P51 unconditional company

P52 I'd stick my fingers through the letterbox to let our dog smell me when I got home and I'd be able to see his tail start wagging through the frosted glass. He would always want to be around people so would follow us around the house, and we'd always have company.

P53 i had bad experiences with a pet that my mother had in the past that didn't like me, but my i have a good experience with my family pet now

P54 getting to know their 'personality' and habits and meeting their needs.

P55 Feelings of affection

P57 i had a cat when I was younger and at that time I did sometimes get frustrated because i wanted to pet the cat. However, I had to learn that the cat also had boundaries and its own will and trying to force it would not work. I like this about cats but I also feel drawn now to dogs because they are more unconditionally loving and (depending on the breed) are probably more affectionate and like to be close a lot more than the average cat does. which is something i like. But I also have learned to give cats distance and as a result they feel safer with me now so they usually just come on their own to me when I'm visiting friends with cats.

P59 I have a good relationship with my pet that They are so cute and I see them as my family. When I was on a vacation without them. When I got home, they hardly ran and hug me. Usually, I always take them with me almost everywhere.

P62 nothing special.

P63 Love and family

P64 i like my dog

P67 Experiences that were important to me were that i got a chance to spend and feel the closeness with a golden retriever, one of the breeds that i love and it is my dream to have one in the future. My neighbour's dog it was but it lived next door and waited for me everyday and listen to music with me.

P69 I love my cat and he was a lovely cat to me

P70 Close friend

P71 Having a pet means having another family member in the house. I think of my pets as people. I feel a little less lonely with my pets around.

P72 Nothing much since there are not much affection between me and fish because fish often avoid with person

P74 Childhood memory, the dog keep chasing me and I'm feared.

P75 sleeping on the same bed

P77 The first time we brought our dog back to our home and when teaching it tricks.

P78 I have once but not really strongly attract to it because it died while I'm young.

P79 Head bunting from my cat feel really goodP80Just everyday experience taking my dogs to walkP81Feel like It is a part of family

P82 I love my dog with him I feel somewhat save and warmth

P85 I think that when i come home from school when my parents were working, they get excited to see me, I feel relaxed all the time.

P86 I own a car so whenever my cat is close to me, I feel like I'm safe and because of this I am very close to my cat and never wanted to leave him.

P88 My pet provided me with company and affection, however I did not feel anxious / emotional if it wanted space.

P90 My dog was not particularly affectionate as a puppy but has become extremely affectionate as he has grown older and now likes to be close to, if not touching, me at most times.

P91 When my rat Logan allowed me to hold him for the first time as he was a previously abused animal

P92 Experiences of training sessions when pup is engaged fully in my words & actions and wants to follow closely with eagerness to learn are rewarding for me as I find the level of attainment is endearing.

P93 Taking care of them (inverts) is the most appealing part of owning them. Ensuring their needs are met and they are thriving to the best of my ability. Motivation to keep them thriving gives me a buzz

P94 A synced bio-rhythm I feel is the closest we can get to pets. We sleep at the same time, eat at time same time, and nap + feel tired. A joined existence is the highest connection there is... and makes me feel at peace and always accompanied.

P95 it enjoyable to have it around, I don't expect anything of it or demand anything, just happy coexistence I suppose

P96 There's no one experience, every day is important and beneficial

P97 Every time they show their tummies, meaning they feel safe with me. Every time they jump on the bed and get cuddles in the morning. When Cedar first let us cuddle him. The one and only time Cedar gave me kisses on my skin. Idk they're my children I love them so much every sign of affection from them makes me happy. I just want to give them the best life.

P98 Being with each other when one of us is ill or just chilling doing nothing

P99 Bringing them up from puppies and training them to be good dogs. They show affection when they notice I am sad or upset and vice versa when they need attention.

P100 It was nice to have a dog that was a companion for walks and other activities (playing football etc) and to nurture him from a young age until the end of his life. It was also a nice presence to have in the house, greeting new people and his routines (e.g. wanting walked or fed at certain times) helped give the household a nice routine.

P101 I think training my dog was important for us to bond; as he learned how to behave and i learned how to take care or something else other than me, and how to do something new and have a positive outcome for him

P102 I have no experiences that are important or beneficial to me.

P103 Not anything comes to mind, I do enjoy their company though at times.

P104 I've had many pets over the years, but chose to answer these questions about one of my currently living pets, a rat named Gizmo (him and his brother Gadget being the first pets I've ever gotten for myself, as an adult, and the first pets I've co-owned and cared for with a partner). Important moments include visiting the breeder to see the rat kits; waiting for my partner to bring Gizmo (and Gadget) home; watching him progress over the first few weeks from being shy to being excited to meet us at the doors of the cage and climb us; introducing him to friends and family; introducing him to novel toys, food and other sources of enrichment (e.g. a box of cocoa fibre) and watching him express curiosity and excitement; teaching him tricks like "come", "spin" and "fetch"; seeing him become extremely cuddly as he got older.

P105 There have been times when I have been sad that one of my cats has come up and cuddled me, this made me feel warm inside and reassured me I was okay.

P106 When it comes to me just to be with me

P107 Spending time with my pet cat ! I believe in animal therapy. My cat helped me so much after the death of my sister . Just being there especially when I was upset . He sleeps beside me every night and is always by my side .

P108 time together. Play.

P109 My "pet" is a working animal. I am not a dog on the sofa kind of person. I enjoy working the dog and interactions during walks.

P110 Honestly can’t remember how I was when I had pets so answered neutral for all

P112 I had a rabbit named Leo that used to lick me, and his show of affection really helped bolster my emotions when I was sad. He passed last December.

P113 Nice when they are around, but I don't depend on them at all

P114 She is like my best friend and child. I love her dearly and sometimes had to leave her for long durations for work trips in the past. It was always difficult but became easier with time. She can be a bit needy in terms of needing pets and gets moody if she doesn't receive that attention. I often worry whether I'm giving her a great quality life or not, whether or not she's happy. I fairly regularly cry when I think about her passing away (she's 12 and a larger dog). I anticipate it will feel like a giant hole has been left in my heart, as we've been together for all of those 12 years. We do as much as possible together --- if dogs are welcome, she is usually there with me.

P115 My pet is incredibly important to me, but he has bonded to my partner and therefore spends more time with him. I get such a boost when my pet chooses to spend time with me rather than my partner

P116 The companionship and relation in and of itself is the benefit, as well as the important aspect.

P117 Having a cat has helped me with anxiety I think but also helps me think about something / someone else when I'm at home as I live on my own. Having a presence just helps a lot.

P118 My my current pet (gecko) I like to handle him safely when he wants, but keep my distance when he is clear he doesnt want to be handled. I care for him but understand reptiles tend to show much less affection than mammals

P120 Waking up in the morning with them near me, Having a routine with them by serving them meals at the same time as me

P121 My cat seems to always know when I'm upset and always stays close. Helps me feel better than just being on my own. My cat also helps me keep a routine of waking up and going to bed with her, as well as feeding times.

P122 When I cry, my pet comes over to comfort me

P123 Dog's are Cute, very friendly, and kinda stupid, and often times have a nack for getting into trouble

P125 Going on walks with my dog helps me relax my mind.

P127 Coming home and her being excited to see me. Training her made me feel happy and fulfilled.

P128 she is my best friend, I am excited everyday to come home and see her

P129 My pet is the thing I look after.

She is a big dog. Who likes her own space and food.

P130 Acceptance

P131 it was nice to look after something and watch it prosper thanks to me

P132 Companionship, interaction

P133 My pet is a horse so I feel like you can build a connection is more easy to other pets going on rides in the nature and exploring together made me feel incredible with them and I think built trust in the relationship

P136 Unforced companionship, interaction by negotiation

P137 seeing my fish happy and health in their tank always my day up

P139 Soothing my cat by reading to him during a thunderstorm.

P140 Getting cat cuddles are so soothing, they genuinely relax me. Hearing a cat purr as you're holding it is such a wonderful feeling, because you know it trusts you and is comfortable and happy with you, and that makes you feel better. It's so lovely when cats you live with just walk up to you and want attention, like when they just sit down on your lap or talk to you and ask to be lifted up. It's a very warm and cosy feeling.

P141 My pets are like friends or family members.

P142 i like hugs from them

P143 I have none

P144 When she comes to be beside me. When she comes to my knee.

P145 Feeling that my pet won't judge me personally

P146 Affection

P147 Caring for something, enriching its life, giving it freedom, watching it be happy/relaxed and bonded with its mate.

P148 I am aware that my pet is a living being and doesn't exist only for my use. It has its own life and will and will choose what it wants. When my pet was close, I was happy to spend time with it, and when it was outside, or in another room, I was happy with that. I felt a strong connection when my pet showed obvious affection, but I didn't feel any upset over the pet not showing that affection all the time. It has moods like I do.

P150 My dog is very happy & chilled

P151 Snuggling up with her on the sofa early in the morning after her first toilet visit of the day when she was a puppy

P152 building a stronger relationship that doesn't surround just food and letting them outdoors.

watching for signs that I have been too overly affectionate them or pushed their boundaries.

P153 Playfulness and companionship, able to be together with few demands (though sometimes there were more demands for attention!)

P154 When the pet shows excitement about you being there. When the pet is comfortable around you, knowing that it feels safe

P155 The sense of a mutual bond and co-operation. The responsibility of caring for a living creature and seeing them thrive.

P156 Affection received / displayed, social interactions, my experience in caring for them and looking after them while they were young.

P157 Pet is always welcoming. Shows trust and affection.

P158 My experiences with my pets are a fundamental part of my life. I am so fond of them and care about them and their health status. I want them to be comfortable and feel at ease with their surrounding environment.

P159 Continuous contentment and satisfaction from feeling I am doing a good job in taking care of them, with little peaks of happiness when they appear to express affection for me, and when they seem happy

P160 Just if the pet around me I feel really happy

P161 Non-judgemental companionship from a thinking being that possesses the capacity for an ulterior motive but A. lacks the intelligence for duplicitousness and B. chooses interaction with me in a way that suggests freedom of choice, i.e. choosing to sleep on or near me rather than in equally warm or comfortable locations.

P162 I feel a deep responsibility, love & care for my pet. We rescued him from a shelter, and from day 1 he was caring & trusting. We have built a bond over time & I cherish the love that he shows me. My pet is a complex animal, with feelings, plans & routines of his own. When my partner is away for a few days, he meows at the door, sits in her chair (he does not usually) and is generally acts different. When she returns, he doesn't leave her side for a day.

P163 Having company during quiet moments/periods of low mood. The pet doing funny things/being a nuisance (in a good way). Helps to reduce loneliness when alone.

P164 Just having the presence of a pet is nice as you don't feel alone.

**How emotionally close do you or did you they feel with this pet? Do/did any specific experiences or interactions feel especially emotionally close or intimate?**

P1 watching tv in front of a fire with it on my lap

P2 Though I moved out of my parents' house where the dog lives and only see him a few times a year now, I still miss him a lot. When I see him, I always feel emotionally close with him when he is excited to see me and greets me in the morning. I also enjoy cuddling with him and just watching TV with him.

P4 Very close; he is part of me, and I am part of him. When he had surgery, I felt like I was close in terms of supporting him, and during moments of struggle for both of us, he will come to me and I would support him. It is just part of the sway of life between us, I do not see us as separate in terms of where he begins and I end (to paraphrase donna harraway)

P6 I love the sitting on the sofa and her sitting on top of me to cuddle moments.

P7 Very close. I was there when she was born and helped raise her from birth.

P8 Very, we sleep together on the bed each night, I have my pillow he has hisP9I was very emotionally attached to my fish! I had a near full-on mental breakdown when the last one died the day before I moved away to University. I thought it was a bad omen and made leaving home even harder. All of my fish are buried in my mom's garden and we had full funerals for all of them because they were part of the family. We would bring them on road trips with us so we could take care of them--they travelled with us every year for Christmas and we gave them gifts. It was an absolute ordeal every time (they lived in a tank we cleaned regularly rather than with a filter, so we would lower the water level enough that it wouldn't spill in the car while the tank sat on someone's, usually my mom's, lap) and we regularly reminisce on how much effort we all put into the ten years we had those fish, but no one in my family would consider going anywhere without them, which is weird because they rarely ever acknowledged that we were there.

P10 Extremely close, 10/10 closeness. The cat was very close to dying at one point, and we helped him back to health, which informed this relationship.

P11 My dog and I are a team. I got her as a pup a week after my father died. I thought I was doing a stupid thing but I'd always wanted a dog and I thought it was the right time. I didn't specifically get her to get me over it. That would have been unfair. I just wanted something else to think about that would force me to go out. She's a comfort to me and I'm a comfort to her.

P13 Very. Our dogs aren’t clingy, but they are bonded to us. They like to be close, and have been known to be protective - tho most of the time they say hi to total strangers, which is cool in its own way, as most people are lovely.

P14 I try to avoid anthropormorphism or over reliance, however I am emotionally bonded and moments of quiet companionship are valuable to me

P15 We're not particularly close. However, when I return home for a visit he still comes to greet me, which is about as clsoe or intimate as we get.

P16 I felt very close to my pet. No specific experiences though.

P17 very close especially when it chose to fall asleep in my presence, and/or chose to seek me out for company

P19 Nothing to add

P20 Physical contact is important. I couldn’t feel as close to any pet where that wasn’t possible

P21 When my pet is ill or has had surgery they are calmer when I am nearby and they can hear my voice. Similarly, I feel very worried if they are unwell and somewhat anxious if I am away from them. As with humans, some animals are more highly strung than others, and require more reassurance. Unlike with humans, absences cannot be explained to animals, causing them distress. This is particularly true of rescued animals who have experienced trauma and hardship in the past. Gaining the trust of any pet is rewarding, but gaining the trust of a rescued pet is a wonderful feeling. That trust is shown by them taking food from you and being happy to eat it with you there, by curling up near you and by sleeping in your presence when their defences are down.

P22 Positive feelings when I saw her in the garden and she didn’t retreat.

P23 I feel extremely close to her because she sleeps in my bed with me every night, sometimes she sleeps in my pillow and nestles into my hair and makes biscuits on me which feels like she is bonding with me.

P24 Felt horrible and helpless when they were ill. Vets fix them up but not much an owner can do other than provide basic care.

P25 This bunny is very bonded to me, she often licks my face and trusts me enough to flop over and fall asleep beside me.

P28 Very close and it feels reciprocated! Honestly pretty much any time the cat will come and lay upon me in various ways (lap, over my arms at the computer, etc.), it's nice to feel the trust that she knows I'm not going to eat her.

In a bit of a different sense but it feels the same way, when there's a medical/body type problem that needs dealing with (stuff in her eye, needs medicine, brushing her teeth, something stuck to her, etc., etc.), usually this requires truly manhandling her in a way that must be uncomfortable. But there is the sense that she knows/appreciates the help.

P29 1. The process of training and improving non-verbal communication 2. Deciphering his intent or needs, enabling or providing them, observing his apparent understanding of my role, and building shared habits. E.g., realising when he wants to sniff something he cannot reach and lifting him, or realising that he wants a particular type of food. 3. His apparent understanding of my state. For example, changing his behaviour when I am sad or sick.

P31 I feel very emotionally close to this pet. She is family to me. She comforts me when I cry, and comes when I call her (rare for a cat)

P32 I didn't realize how emotionally close I was to her until she died

P33 Very close. In terms of specific interactions that make me feel closer - if he's been unwell and we've cared for him, he always becomes more affectionate for a time. That specific behaviour is quite touching.

P34 most experiences/ interactions are supposed to be "close" otherwise they would not be your pet. Or they would prioritize other vs you as owner

P35 Very much

P36 Cuddles in bed

P37 Very emotionally close.

P38 I really liked her. Towards the end I think she really liked me.

P39 I would literally take a bullet for this cat. I like when he chooses to be near us or spend time with us.

P41 Very much. I watched him die in my brother's arms, because the vet was unavailable to euthanise him.

P42 My cat would come near and sleep on my lap after purring. She was a playful small cat who used to chase anything that it sees as a mouse. Used to do mischievous behaviour like jumping and scratching and then made the most innocent face when held up. Felt a little bad because it used to be bored at night when everyone were sleeping and sometimes meowed loudly.

P43 I wasn't extremely close to it, but enough to love and miss it. Not really.

P45 I feel fairly emotionally close with him when I'm living there over the summer. Physical interactions feel most intimate, like when I'm petting him and he's just lying down on his stomach, letting me pet him. Sometimes when he lies on his back and I rub his belly, he puts his paw on me. That also feels intimate like he's petting me back.

P46 I feel very warm, happy, and glad that they get close to me and make me feel better when I was sad.

P47 Love her, but can accept that she is doing her own thing. She does not have a duty towards me. I like caring for her - she does not have to 'pay back' through being cute or cuddly. That's not why I have a pet

P48 Caring for it during its end of life care. I felt they understood that I was doing something kind for them, giving them medicine and hugs. They displayed more signs of affection at this time than any other time.

P49 I don't feel emotionally close to any of my pets. One lives in a tortoise enclosure and the others live in a fish tank. I am unsure if they even know who or what I am. I wouldn't really say I feel emotionally close to them. I care about them and I want to be a responsible and caring owner to them, but I don't feel like they have the capacity to be emotionally close to me.

P50 She is not a very affectionate cat 90% of the time, so when she comes to cuddle or sit on my lap it feels quite significant and intimate. She is my parents cat so I only interact with her when I go home, a few times a year

P52 When our dog would choose to sit on/next to me rather than his usual places, it felt like a more personal choice than just comfort or routine. He would also sleep with one leg draped over the person next to him so he could tell if they moved away.

P53 it is not mine, but when i was at my parents' home i felt connected with him

P54 quite close. my budgie liked a certain genre of music, would make annoyed noises at other styles, but sing along to what he liked.

P55 Quite close

P57 i did rescue it so that was quite a bonding experience. It was hard to convince my parents to keep it so i think that led to me feeling very close to the cat.

P59 I would say that I have a close relationship with my pets. I missed them every time when I was away.

P62 nothing special.

P63 I feel really close with this pet like one of my family. I really miss my dog especially when after I came home it will always run to me and try to play with me.

P64 i love my dog

P67 I felt quite emotionally close because there was a time as i mentioned the dog came and sat next to me while i patted him and listen to muisc, it would wait for me to come out and bark if i didn't come and if he saw i was out he would make those cute puppy eyes and wait for me to come hold it's hand and pat him or feed him.

P69 I feel really good with my cat especially when it lay down and turned its belly up on me, however I don't like to my cat to sleep with me on the bed at night because it maybe unhygiene.

P70 He was annoying sometime, but I like the way he is.

P71 I would save my cat's even if it meant sacrificing mine

P72 Depend on type of pets, if it was a dog I would show more affection but the fish as pet to me is I felt neutral.

P74 I feel neutral

P75 very close

P77 None in particular, just being around him is nice.

P78 A bit

P79 Neutral

P80 Close but family does not allow dogs inside the house so not as close as it could have been

P82 It not that close since I have him not that long till my mother gives him to my brother because she can’t live with fur all around the house. Cuddle my dog till sleep

P85 I think that I understand them, I do get very close to them but also sometimes depending on their mood. I only hug them and rub them.

P86 I feel close to my cat and attached to him. he often come to me when I woke up straight away, so this make me feels good and comfortable around him

P88 Very close, and I missed him greatly when he passed away. It was lovely when you got home and he was waiting for you wanting attention.

P90 I feel extremely emotionally close to my pet.

P91 Id love to be closer to him but he's a nervous animal so I am happy if he is happy

P93 Inverts only have primitive brains so I don't think they have any feeling towards me, and that doesn't bother me at all. My positive feelings for them and love for them is out of respect for how interesting they are. My emotions are tied to fascination of how they exist and caring for their needs.

P94 I would have a mental breakdown if my pet dog would get sick, or started getting seizures, needed surgery etc. My days would be ruined and I would near about throw up from stress. My reaction would be that of any person I am close to if not more stressed because of their short lifespan

P95 emotionally quite close, A good friend

P96 I feel very emotionally intertwined with my animals, they are all loving and affectionate while being thier own beings

P97 really really close. there are some things that they do with me that they don't do with others, or things they let me do like grooming because they know they can trust me.

P98 I’ve been with my pet for over 10 years and we are practically inseparable, I’ve been through mental issue and they have been there for me and I’ve been there for them when they were very ill

P99 When I cry, they come and sit with me and make sure I am okay. When they bring me their toys to play and spend time with me.

P100 I was very close having him from about 13-26 and when I was a teenager I couldn't imagine life without him. However towards the end when his quality of life was becoming poor (struggling to get up/ falling over etc) I felt more content that it was his time to go. I still miss him but am grateful for the time and memories we shared (walking, swimming, playing football etc). Holding him as a family as the vet put him to sleep was also an incredibly moving experience - probably one of the strongest in my life.

P101 i consider my dog very close to me, as i take care of him every day and he greets me every time i come home. i feel like the dog sleeping in the same bed as me, or on my floor, is very emotionally intimate and i think its cute

P102 I have no specific experiences or interactions with my pet. However I do believe I am emotionally close to my pet as it has been around for a large period of my life and has became a part of everyday life

P103 I feel quite close to my cats as I feel I can understand them but since I don’t normally see them often. I’m not overly attached to them

P104 During our daily free roam sessions, oftentimes Gizmo will settle in on my or my partner's lap for cuddles. The first time he did this felt very special, as do the times when he's so happy to be petted that he begins bruxing and boggling.

P105 I feel somewhat emotionally close, especially when they give me head boops which makes me feel like I am one of their own.

P106 My outdoor rabbit coming indoors to give me my slippers when i get back from work

P107 My cat is always beside me . He waits for me to come home from uni and loves love. He sleeps beside me with his paw in my hand at night . He gets annoyed when I’m at my computer desk and doesn’t stop meowing until I allow him to sit on my legs while I do my course work .

P108 very, sad if im not with him

P109 I like my dog but as previously stated she is a working animal not a member of my family

P111 they slept in my bed with me

P112 I had a rabbit named Leo that used to lick me, and his show of affection really helped bolster my emotions when I was sad. He passed last December.

P113 Close but not dependent

P114 I love when I see her in the morning or coming home and she looks so happy to see me (I feel so happy to see her, too). I love when she's able to sit nearby without needing ongoing petting (that's my only "gripe" with her is that she won't just cuddle - you must actively pet her nonstop if you want her to cuddle up with you). I love our mornings together, and I really feel excited for her when I can see she is having fun, playing fetch at the beach or getting a tasty treat, attention, etc.

P115 My pet was a rescue animal, so seeing him blossom in our loving home is wonderful. I feel very emotionally close to him, even if the feeling is not necessarily (always) reciprocated!

P116 Quite close. After having been apart, be it for a few hours or several days, it's fulfilling to be reunited with such happiness and enthusiasm.

P117 I think it varies but I'd say especially when I pet him on the couch, if he's laying on me or carry him.

P118 He and his health are important to me, and when he takes food or is comfortable with me it is great! but there is much less connection than I have had with dogs in the past

P120 Very Close, When caring for them when they were recovering from being ill

P121 I've very emotionally close with my pet. My cat doesn't let many people pick her up without scratching or biting first, but she is fine when it's me. Makes me feel important to her.

P122 My pet relies heavily on me

P123 I am fairly close to my pets but, I also live in uni half the year so I don't get to interact with them very often. (6 or 7 out of 10) also have had enough pets die I am fairly detached from that.

P125 I feel as though my dog can trust me and is a good family dog.

P127 When I was sad she would come into my room and lie on me, it was like she knew I was upset or not happy

P128 Very emotionally close, she is very intuitive

P129 We sit together and play together. I feed her. She loves food.

P130 Very. Yes.

P131 reasonably close, hamsters don't emote very much but they are curious. mine would watch me play the piano which i thought was nice

P132 Not very - feeding through cage felt closer

P133 I am very close but sadly I don’t get to see her often anymore however most emotionally close moments occurred after working with the horse either in competitions or intense days where I would take care of her like brushing washing and feeding her

P136 Cats electing to spend all their time with us if we’re ill

P137 when feeding them i always close when they knew that light moved so it was feeding time

P138 Very close

P139 He used to sleep on me during the day, at night time, or really any time I was still.

P140 Very emotionally close, cats have a way of looking deep into your soul when you connect with them. It's like they tap into some part of your being. I've always felt very close with the cats I've had.

P141 I felt extremely emotionally close to my pets.

P142 i am very close to them i love them so much. I very like when they climb on my knees

P144 I feel very emotionally close with her. When she choses to come and sit on my knee, and purrs, while kneading my legs with her paws.

P145 Fairly close, it is always nice when my dog chooses to sleep with me

P146 Quite close. I was young. My guinea pig would run up my body and sit on my shoulder and snuggle against my cheek, then lick my face. It was caring for me.

P147 Caring for the pet when sick. Syringe feeding, medicating. Also when they choose to be in your space/company.

P148 Close enough that we're aware of each other as living in the same household. My pet comes and goes for days at a time. It chooses to stay with me, and I strongly believe that it's on its own will - I don't dictate that. When previous pets have passed, I've felt upset, and I've cried. But I'm glad for the time I spent with it, and I don't regret my attachment style to it.

P150 My dog is well looked after and made to feel loved

P151 I am extremely close to her, I didn't know how much I would love her before we got her.

P152 cuddles with this pet makes me happy and content especially when they're willingly giving them to me; especially before bed and in the mornings.

P153 I still miss this pet terribly after he has been gone more than 10 years. It would feel particularly close when he would nap on me, or next to me while I was doing something quiet.

P154 I feel emotionally close as it is clear the pet loves me especially when it shows affection

P155 I feel very emotionally close to my pets. The experience of living alongside them and watching them grow is central to that. Interactions where they show their trust and affection, e.g. by sleeping on your lap help to feel close. But also things like caring for my elderly cats through their last days, and final goodbyes are incredibly intimate and strengthen your bond, even though they are very hard.

P156 How and when they choose to spend time with me or request attention from me.

P157 Very close. Shared affection at all times. Pet being instinctive with affection at difficult periods

P158 I feel so close to them. They speak their minds with their language and need to be respected because of that. They have their inner bubble that is not to be invaded.

P159 Relationships shifts from novelty and discovery, with tensions of learning from each other, to more familiar routines of being old friends. A favourite time of day is when I sit and read, with my big dog’s head on my lap, as he shifts, groans and grumbles in comfortable satisfaction. He is a reserved, self-contained, dog, so it’s all the more meaningful when I feel that I have earned affection and trust from him.

P160 yes very close to me I love my pets

P161 I would say fairly close. Physical closeness and emotional closeness were hand in hand, particularly that my own interactions were tolerated (I hesitate to say "welcomed") while those of others were dismissed or rejected. I liked being someone's favourite.

P162 I feel very emotionally close to my cat. When I am sick, or having a depressive episode, he sticks to me more than usual, and I feel that he understands something is wrong. I had food poisoning last year when my partner was away, and I spent the entire night curled up between the floor of the bathroom & hallway. He usually sleeps through the night as he is old, but he spent the whole time with me, brushing up against me, trying to lick my face & hair, and generally comforting me. When my partner is out of the country for weeks visiting family, we spend a lot more time together. It seems like he misses her, and wants to spend more time with me, sitting closer to me than usual, and scratching at my door in the night (he does not usually do this)

P163 Very close. Especially close when spending quiet time together/company.

P164 My pet often sleeps in my bed at night which is nice.

**Do you think there are positive pet-like experiences which you could share with a robot? If so, what kind of robot, and what of positive pet-like experiences do you think you could share?**

| **PID** | **Sentiment?** | **Robot Experience Types** |
| --- | --- | --- |
| P1 | Unsure | i think it would have to be long term for me to understand. but for me my pets have "free will" - i.e cat goes outside and does its own thing all day and satisfies its own desires, terrorises the ecosystem etc. and then i get to see it in the evening. i think if you liked dogs this works way better |
| P2 | Probably yes | Cuddling with a soft robot would be nice, getting greeted by the robot also. Probably in general being acknowledged by a robot might be nice. |
| P3 | Probably yes | Companionship, helps to me relax and makes me feel loved |
| P4 | Definitely not | part of why i like my pet is because he is alive with personality, robots do not have this. |
| P5 | Probably not | There are positive pet-like experiences I could share with a robot. For example, a companion robot designed like a friendly dog could mimic playing fetch or responding to touch with affectionate gestures. This would provide companionship and stress relief, similar to what people get from interacting with real pets. |
| P6 | Definitely yes | Cuddling and talking (even if you do not get a reply) |
| P7 | Probably yes | something more similar to the seal robot with a semi realistic look and mannerisms. Too realistic may have uncanny valley vibes and result in being off putting, and too much interaction may result in it feeling gimmicky and making me loose interest. For pet like experiences something simple and more like real life would be better, as something that resembles more of a personal assistant that can take song requests or do performative actions would actually make me feel less attached on a personal level. |
| P8 | Probably not | N/A |
| P9 | Probably not | none |
| P10 | Definitely yes | A zoomorphic robot that can simulate feelings of togetherness, closeness, and similar could definitely create those experience. |
| P11 | Definitely not | None |
| P12 | Definitely not | No, I have little experience with a real robot. |
| P13 | Probably yes | Mostly cuddling up to something warm and gently responsive. |
| P14 | Probably not | I suppose a robot could give the same 'pet-like' experiences that, say, a teddy bear could. But that has little to do with them being a robot. |
| P15 | Definitely yes | I think it would need to be a robot that didn't speak or look like a human. That wouldn't feel like a pet, but more like a friend? And those are different experiences. And I think I'd probably want a robot that had some agency of its own, moved around or something. I like birds a lot, so maybe a bird-like robot?  I think from a pet I look for experiences where we are coexisting, rather than directly interacting, so I'd like a robot that could hang out with me in the same space, maybe come over for interaction every now and then, but is otherwise doing its own thing nearby. I'd like to have a little pal that would sit with me while I'm at the computer, or watching TV, and the rest of the time is off doing its own thing, checking in every now and then. |
| P16 | Probably not | The robot showing excitement to see you, probably. |
| P17 | Definitely yes | I would enjoy sitting with a companion robot with soft fur and cute vocalisations |
| P18 | Definitely yes | I think if a robot looks and behaves like a pet in a few key aspects like softness, warmth, size, and responsiveness, then I think I could bond with it a little like a real pet, in that I find comfort in them and feel affectionately towards it. Also I think interacting with a type of pet robot that I could play with and 'train' by teaching tricks too, would be a positive and rewarding experience. |
| P19 | Probably not | Feed it and look after its needs. |
| P20 | Definitely not |  |
| P21 | Probably yes | I have experience of keeping several Furbys fed and cared for when my children forgot to. I enjoyed the experience but it did get stressful when three needed fed all at once! I had similar experiences with Tamagotchis and found myself feeling sad when they ‘died’. I think robots are useful in teaching dependency and routines. Having worked with children and young people with special needs and neurodiversity I can see that robots such as these would enable them to get positive feedback from a friendly source with more predictability than from a living pet. |
| P22 | Definitely not | Something fluffy might be a stress reliever for some people. I’d spend all my time working out the algorithms. Most of the people I worked in in IT weren’t allowed other living organisms in their rooms. |
| P23 | Probably not | stroking it |
| P24 | Definitely not | None |
| P25 | Definitely yes | I think a robot that looked like a traditional pet (dog or cat) that moved and reacted naturally and with affection would be best case scenario. Or a little robot, that looked like a robot, and had a big personality that could respond to emotions would be most positive. |
| P26 | Probably yes | Gentle stimulation, reassurance from tactile sensation |
| P27 | Definitely yes | I would prefer a robot that has an expressive face and can move and respond to interaction. It would be nice if it was cuddly and furry but there’s a risk of it looking creepy. I wouldn’t mind if it looked more robotic and felt like a new kind of pet. I think it could be good company, fun to play with, teach tricks, and watch it learn more about me and its environment. |
| P28 | Probably yes | Biased toward cat-like experiences of course but the experience of a robot *wanting* affection (i.e., petting) and seeking it out. And then me wanting to give it back. The actual feeling of petting itself, the reaction of it (purring, nuzzling, etc). Being missed while away and waiting at the door for me when I return, etc. |
| P29 | Probably yes | 1. I could be impressed by it doing something I did not think it could do 2. I could admire it aesthetically 3. I could enjoy analysing and understanding its behaviour 4. I could profit in small ways from its labour e.g. guard duty or other proxy awareness or cleaning/fetching/pulling a sled |
| P30 | Unsure | Stress reduction and company for old people and people that are lonely |
| P31 | Probably not | N/A |
| P32 | Probably yes | A robot that could go out with you when taking the dog for a walk. |
| P33 | Probably yes | I think the passive style robots are more pet-like. Docile affection, warm, soft. They don't move so they don't appear quite as uncanny. |
| P34 | Probably yes | Fetch/ Interactive robot. |
| P35 | Unsure | No idea |
| P36 | Probably yes | Companionship |
| P37 | Definitely not | I don’t know. |
| P38 | Definitely yes | Only with a future robot. current robots are too limited. |
| P39 | Definitely yes | Bonding, playing games, etc. |
| P40 | Probably yes | companionship, pet training |
| P41 | Definitely yes | Any kind. But it would have to show unpredictable, not pre-programmed behaviours. It would have to jump onto my table and walk over my keyboard while I'm typing or suddenly and without provocation or warning scratch me violently on my arm which seconds ago had been stroking him. |
| P42 | Probably not | Maybe, just talking out in front of the robot. |
| P43 | Definitely yes | Seeing it moving funny or making funny sounds |
| P44 | Definitely not | N\a |
| P45 | Definitely yes | Likely animal-like robots, like petting it and getting positive feedback in response. Or alternatively, some sort of soft robot that liked to rub up against me like a pet. Proximity would probably also be a potential positive pet-like experience that I could get from a robot â€” just that feeling that someone else is here with me. |
| P46 | Definitely yes | zoomorphic robot and cleaning robot |
| P47 | Definitely not | N/A |
| P48 | Definitely yes | Sleeping and cuddling with warmth and perceived feelings. |
| P49 | Probably yes | Having something 'alive' around the house, though without necessarily wanting or needing to form an emotional bond with it. |
| P50 | Definitely yes | I could see an interaction with Qoobo sort of like interacting with a cat; something that doesn't seem to care if you are around or not but is cute and you want to cuddle with it. |
| P51 | Unsure | NA |
| P52 | Probably yes | I think I could get a low level of companionship from a zoomorphic robot like Paro just through physical contact and some reactivity. But it would likely be of more infrequent use due to the limited range of communication and expressions. As those expand/improve I could see more usage. |
| P53 | Definitely not | no |
| P54 | Probably yes | cute and expressive. |
| P55 | Probably not | Cute, fluffy, some movement. |
| P56 | Probably yes | Some level of play (particularly the first robot) could be replicated, without needing to clean up after it. |
| P57 | Definitely yes | i have interacted many times with PARO. Paro looks cute, has an appropriate weight that feels nice on the lab, and responsive to one's touch. The sounds can be a little much sometimes but the fur is very soft and it feels nice to interact with PARO while one is working or watching a movie as it keeps one company and provides tactile stimulations. |
| P58 | Probably yes | zoomorphic robot, it could keep us company |
| P59 | Probably yes | When I was young, my mom bought me a dog robot which we can control him by using remote. It is very old robot when comparing in today but I still like it. She bought it before we got our pets. |
| P60 | Definitely not | - |
| P61 | Definitely yes | zoomorphic, petting |
| P62 | Unsure | Nothing can comprehend real pet. |
| P63 | Probably yes | Zoomorphic robot, I think when the robot seem to care about me |
| P64 | Probably yes | companionship? |
| P65 | Probably yes | maybe cuddling and hugging I guess |
| P67 | Unsure | Playful, emotions of sadness and happiness without any biased judgement |
| P68 | Probably not | a cute roomba that's both useful and adorable |
| P69 | Probably yes | Cat |
| P70 | Definitely not | Nothing |
| P71 | Probably yes | I want to have a pet that can talk to me. It could tell me what problems it has so I can save it in time. |
| P72 | Unsure | Dog pets would be good to be the partner and interactions in my opinion |
| P73 | Probably yes | Petting, simple games, simple tricks, etc. |
| P74 | Unsure | ask for hand |
| P75 | Definitely not |  |
| P76 | Probably yes | Appearance |
| P77 | Unsure | None |
| P78 | Probably yes | dog bot so it surveillances and provide a friend |
| P79 | Probably yes | Anything that feel fluffy, small, and feel good to hold |
| P80 | Probably yes | Watching tv and sleep together |
| P81 | Probably yes | Animal robot |
| P82 | Probably yes | A robot like Cosmo, maybe play with him in free time |
| P83 | Unsure | i haven’t had pets before, neither did I had played with any pets so i don’t know |
| P84 | Definitely not | No |
| P85 | Probably yes | I think that I should be like unintentional movements like the real pets. Sometimes they have like sudden emotion spikes I feel that are nice to ensure that it is more fun around and to really feel that it is around not waiting for us to interact with it. |
| P86 | Probably yes | friendly looking pet-like robot are probably a good one. the goal of it is not to scared anyone but needs attention of the owner of it. |
| P87 | Unsure | Sorry, I have no answer for this one. |
| P88 | Probably not | I don't think that there are any |
| P89 | Unsure | I am not sure. |
| P90 | Probably not | N/A |
| P91 | Unsure | I'm not really sure? I had a tecsta as a kid and it was cool but it wasn't a real dog |
| P92 | Unsure | . |
| P93 | Probably yes | Something like a Tamagotchi style pet, where you respond to it's needs and see a response according to how you're caring for it. You have success/failure in caring for it, a measurable achievement in some way. |
| P94 | Unsure | Funny little accidents, bumping into walls, falling off something, being curious about items. All are innate "human" accidents, such as seen in babies. |
| P95 | Probably yes | potentially if it can react to itself and it's environment in greater detail, it would useful for activities I pursue like climbing |
| P96 | Probably yes | I think one of those Boston Dynamics dogs could be build with pet-like interactions and fur with a little pixel robot face. Having robot dog go for a walk and sleeping at the end of the bed could be nice |
| P97 | Probably yes | I don't know how shared these experiences would be as much as they would be one-sided for the person. the humans could probably feel comforted and soothed and delighted with the play and affection, but I don't know this would be felt by the robot |
| P98 | Definitely not | None I don’t think a robot can show affection |
| P99 | Unsure | can provide positive pet-like experiences that reduce stress and anxiety |
| P100 | Probably not | Can't think of any but I am not entirely closed off, let's see what the future will bring |
| P101 | Probably yes | the first robot was the most realistic to me and i could see the importance of people getting that as a substitute for a real pet if they still want the love and affection but are unable to take care of a real animal so the robot seems lifelike and interactive enough instead |
| P102 | Probably yes | I do not believe I could share a bond, however I still believe I could have a positive experience just through petting a robot such as petit qoobo. |
| P103 | Unsure | Uncertain. |
| P104 | Unsure | I think Cozmo could be fun to play with, he looks like a fun character to watch and interact with. |
| P105 | Unsure | Unsure |
| P106 | Definitely yes | Pets as ornamental things is a concept i like. A beautiful lizard in a stunning vivarium is no different from a robot lizard. Both are there to be looked at. A cat or dog is more interactive. They are your child and need your care. |
| P107 | Unsure | Robot cat  Not sure on what experiences |
| P108 | Probably not | none |
| P109 | Probably not | lap pet for someone in a old peoples home |
| P110 | Definitely yes | The little smart robot (can’t remember the name) seems like a great companion and I want one hahahah |
| P111 | Unsure | ?? |
| P112 | Probably not | I do not know. |
| P113 | Probably not | N/A |
| P114 | Definitely not | n/a |
| P115 | Unsure | Interactive play and cuddling |
| P116 | Probably not | This field had to be filled in order to proceed, but my answer was probably not, so I have nothing to add here |
| P117 | Probably not | In one of the experiments, I had the robot Paro with temperature heating on my lap. I would like that if I'm feeling anxious at work for example. |
| P118 | Probably not | One that doesn't try to be too lifelike and is functional, whose suitability improves over time by learning from your interactions. One that can communicate expressions but does not try to simulate real emotion |
| P119 | Probably yes | Definitely more familiar look and tactile would be nicer. In terms of personality, I like the first robot, because it's small and not invasive with its full of character. |
| P120 | Probably yes | In the case of Qoobo, petting them to give the response of cat purrs would be enjoyable. |
| P121 | Probably yes | If the robot could mimic the physical presence of a pet, that would bring lots of comfort and make it a wanted companion. Something with warmth that could move without feeling like just a robot. |
| P122 | Probably yes | Provide positive feedback when calling pets |
| P123 | Probably yes | having a walking chatbot that could help identify and solve problems would be cool, like asking about a problem with code and having them help out or having it come and bother you about a alarm rather than just screaming and stationary. Or allow it t call emergency services in case of disaster could save peoples lives. |
| P124 | Probably not | nothing |
| P125 | Unsure | I don’t know |
| P126 | Probably yes | Companionship and entertainment |
| P127 | Unsure | N/A |
| P128 | Probably not | Unsure |
| P129 | Probably yes | I can care about video game npcs. So I can probably care about a robot. |
| P130 | Definitely not | Robots are machine, tools, not sentient beings. |
| P131 | Probably yes | you can anthropomorphise anything that emotes, animals and robots alike. if you perceive something as smiling back at you it makes you happy. |
| P132 | Probably not | N/A |
| P133 | Definitely not | N/A |
| P134 | Probably yes | Petting it would most likely be soothing if it was soft. Watching it react to the world could be interesting. |
| P135 | Definitely yes | If it looked and acted like a pet then i would see them as a pet. |
| P136 | Definitely not | None |
| P137 | Probably yes | cuteness and playing |
| P138 | Probably not | - |
| P139 | Probably yes | Cuddling or petting one of the more tactile companions |
| P140 | Definitely yes | Talking to them definitely, the companionship in general would be similar. Playing with them, being comforted by them. |
| P141 | Probably yes | play with a robot which is not too like a human or a real animal |
| P142 | Definitely not | - |
| P143 | Probably yes | The same with normal pet, petting him or having fun with him. |
| P144 | Definitely not | none |
| P145 | Probably yes | Any sort of robot that could navigate its surroundings would be at least entertaining to watch |
| P146 | Unsure | It would have to be very realistic and intelligent, and then you could share the majority of things you would share with a real pet. |
| P147 | Definitely not | Na |
| P148 | Probably not | I don't believe a robot, following these videos, can necessarily replace life. It can imitate, but it doesn't replace. |
| P149 | Probably yes | It has to be a cute and expressive robot. You can have learning experiences if they have the skill to teach. Or you could play with them |
| P150 | Definitely not | My answer was definitely not |
| P151 | Definitely not | N/A |
| P152 | Probably not | I do not know. I feel that robots that off lift household chores seem almost acceptable but not completely welcomed. |
| P153 | Definitely not | N/A |
| P154 | Probably yes | Interactivity and a presence feeling |
| P155 | Definitely not | N/A |
| P156 | Definitely yes | Pet like experiences for me would mostly be some form of companionship. So not necessarily doing anything, but I believe it would be a positive experience for me just having it in the background and occasionally either one of us choosing to interact. |
| P157 | Probably yes | welcoming/show affection |
| P158 | Probably yes | The one with a fluffy tail looks just fine. |
| P159 | Probably yes | Some of my experiences are from me, projecting anthropomorphic exchanges of feelings, intentions, reactions, emotions etc that are either only faintly present in my pet, or exist only in my imagination at the time. It’s possible that some of those experiences could grow gradually over time, especially if they grew from some initial relationship based on a satisfying function, form, and learning opportunity (where I felt I was growing by learning, or being creatively rewarded by exercising my curiosity). I wish I could see a little more functional utility in it, as a collaborative starting point for a relationship - even if was mostly as a way to rationalise using it, as a heated massager or a roomba-like table duster (that thoroughly cleaned itself!) |
| P160 | Unsure | I do not know |
| P161 | Definitely yes | "Pet-like" is an important qualifier here because there are aspects of the cited robots that are pleasant, such as being able to pet something soft and warm, prompt a cute reaction and at least pretend to be caring for something other than oneself. The kind of pet-like experiences I envision are much like that, lacking the independence of thought that a real animal would have. Alternatively, something that combines the cuteness of the Qoobo with the motility of a Roomba might be a winning formula. It's not a real animal, but it could be a real pet. Sort of. |
| P162 | Probably not | I do not think that I could share positive pet-like experiences with a robot |
| P163 | Probably yes | Interaction, entertainment, ‘petting’ (for soft/fluffy robots) |
| P164 | Probably yes | You can spend time and 'play' with a robot but I don't think it would be the same as having a pet. |

**What do you perceive as the key differences between a real pet and robot pet?**

| **PID** | **Key Differences Pets and Robots** |
| --- | --- |
| P1 | to me yes free will and the "aliveness". what is consciousness is very philosophical but that for me. because we cant have a natural language interaction with a robot pet (well maybe we can, but i don’t think that’s the point) like we can with a robot, even though its the same underlying principle, i think that’s a big thing for me |
| P2 | Robot's lack the sophistication and probably the energy of a real-life pet. |
| P3 | I do like to have a robot pet that I do not have to clean after |
| P4 | one is alive with complex emotions |
| P5 | The Sense of Life |
| P6 | A real pet has agency, does things by itself. While some of the robot examples to have that, more do not and it is not clear where the reactions from a robot come from. |
| P7 | not actually living. currently also can't reciprocate any affection. for better or worse they are unchanging too (don't grow old, etc, but also the relationship won't change or develop past a certain point) |
| P8 | It's all about love  I don't think I could love a robot pet like a real pet  Like how I couldn't love a robot woman (even if if was objectively Hot AF) like a real woman |
| P9 | Real pets are unpredictable and are living beings who are trusting humans with their care. Robots are likely more predictable but where's the fun in that? |
| P10 | Unpredictability is part of the appeal of real pets - that independence is crucial to the lifelike aspect, which is one thing all referenced robots so far have lacked. |
| P11 | One is alive and the other is a robot. |
| P12 | The robot pet's behaviour is obviously different from what a real pet does. I've never seen a real life-alike robot, I mean there can always be some signs. |
| P13 | Robots don’t need actual care, and are unlikely to counter-surf or be brats. This means, I guess, that they can/could give comfort and positive ‘support’ without requiring much effort or true commitment from the owner. |
| P14 | Pets like cats or dogs have agency (or something like that). They are living animals with minds of their own. Getting their affection involves building a bond, not something programmed into them. |
| P15 | I definitely think there could be a robot pet that doesn't feel any different from a real pet, so I'm thinking about current state rather than conceptually. I definitely think a sufficiently advanced robot pet would be indistinguishable to me from a real pet. Existing robot pets don't seem to be as alive as a real animal -- they don't have as much independence and agency, and a lot of them aren't as expressive as a real pet would be. Those are I think the main differences. But like the wee Cozmo guy, I think he's basically a real pet. |
| P16 | I think that real pets are conscious beings and have to potential to show real affection. |
| P17 | Lack of physical warmth, lack of softness, knowing interactions - like coming when called, seeking me out for interaction etc |
| P18 | I think with a robot pet I would be aware that it couldn't truly love me back, and that would probably stop me getting as invested as with a real animal, so I might get bored after the novelty wears off. Also a real pet you can't turn off and put in a cupboard, so I think the responsibility that comes with that, and the resulting bonding that can come with taking care of something that needs you is hard to truly replicate in a robot pet. On the other hand, the differences between the upkeep of a real pet and a robot pet make having a robot one much less costly, and less of a time investment, which is a big pro. |
| P19 | The robot has an off button |
| P20 | A real pet is real, with real emotional connection and a living, breathing thing. A robot is a very poor mechanical substitute. Why choose fake when you can have real? |
| P21 | There is unpredictability with a real pet, plus actions such as providing food, cleaning, illness and other real life situations. |
| P22 | One is organic - even if it’s not particularly intelligent - so any recognition or affection shown is not a synthetic result of coded programming |
| P23 | You can raise a real pet from a baby and watch it grow, you can breed it. A real pet has a natural smell, is unpredictable, vulnerable and does silly funny things. A real pet has a unique character and personality which is shaped by its upbringing. The legal responsibility for a real pet is not a joke and can have legal consequences. I like making memories and taking photos of my real pet, knowing that one day it will die because life is finite. I would thank a real pet in my PhD thesis. You can be allergic to a real pet. You need to feed a real pet and it can get sick. |
| P24 | Empathy, pain,, joy, feelings |
| P25 | Robot pets seem to lack ability for empathy and affection. |
| P26 | Robots have no emotions other than those we superimpose onto them, and do not express free will. Also less upkeep. |
| P27 | A robot pet requires less responsibility and wouldn’t violate my tenancy. However, I would feel less attached and emotionally invested in a robot. |
| P28 | Thinking of the previous question. I guess that want is not always there? Real pets don't always want interacted with. They've got other stuff to do! |
| P29 | Mammals at least have an immediate appeal to empathy that I think is probably easier to manufacture through biological technology than traditional robotics. Real pets are more valuable for their approximation of friendship and for their ability to inspire a genuine concern for their wellbeing. Robot pets are move valuable in a way closer to a project/experiment like a customised vehicle or piece of art. |
| P30 | Real pet requires food |
| P31 | A real pet feels things and can act autonomously |
| P32 | A Robot is not a pet, I feel as thought it is more a toy for any age. |
| P33 | Robot pets are not sentient so the relationship with them from my perspective is not really meaningful. They are closer to toys than pets in my eyes. |
| P34 | robot pet most likely will have more accurate and repeatable task/ list of things that they want / like / do. vs real pet do thing randomly |
| P35 | We don't have "live" robots yet, it's human programming. |
| P36 | A robot would be more useful - e.g. accomplishing tasks |
| P37 | Consciousness, free will, emotions |
| P38 | Pets can learn over time. Current robot companions do not. |
| P39 | I think it’s the ability to make decisions on their own. With a robot that’s purchased, it’s almost guaranteed to like you, you know? With an animal, you have to truly earn their trust over time. |
| P40 | real pets can surprise you with their unexpected actions, robot pets do not (yet) |
| P41 | What robot pet? As far as I am concerned, there are no robot pets. On a ontological level, we all are not much more than organic cellular machines. There's no principle difference between a robot, a human or another animal. |
| P42 | Consciousness. As a student studying Computer Science, we know that these robots or even Generative AI like ChatGPTM Deepseek, etc. are just machine learning which is just mathematical models in the backend. The use of artificial neural networks is interesting but nothing compared to the real lives of biological beings on this planet. Sometimes, these chatbots go rogue and their is a huge buzz on the Internet which is expected but we know that these AI chatbots suffer from hallucinations. Even my parents or the older generation are shocked to see these AI applications and believe they might have some real soul, but as students we know these are just applications of AI which is very impressive but nothing compared to the awareness that biological lives have on Earth. |
| P43 | The fact that we, humans, know real pets are alive and that they also feel happiness, suffer or are nervous. This is something that in robots we cannot assure |
| P44 | A robot doesn't have emotions or intentions |
| P45 | It's difficult to say for sure, but I think it has something to do with general capabilities and behaviour. Pets can do a lot more and are a lot more independent â€” a dog can get anywhere it needs to mostly without me, and is independent and autonomous enough that it seeking out affection or attention is a lot more meaningful to me. Knowing that it can think for itself also makes it more special â€” a dog isn't programmed to love me, it simply chooses to. |
| P46 | A real pet is sometime can be very hard to handle because if you don't have any time and experience to take care of them. I would say the pet robot would be the best choice to choose. |
| P47 | I know that pets are living beings, robots are not. |
| P48 | A robot pet can be switched off, doesn't need cleaning up after, doesn't smell, can be left alone for long periods of time without care, won't get sick, can't have real feelings so can't be hurt or sad. |
| P49 | With real pets I think it's important and responsible to make sure they are properly cared for to avoid physical and emotional harms. With a robot pet I think there would be less anxiety related to that. There are probably similar analogies (e.g., the need to charge or replace batteries, the need for maintenance to moving parts) but I'm sure a robot pet wouldn't come with the same needs for attention like bathing, cleaning, feeding, habitat maintenance, etc. |
| P50 | I think the differences would be knowing that the real pet is alive and has it's own thoughts and feelings, rather than a robot which is preprogrammed to react to the world already |
| P51 | emotional connection |
| P52 | Unpredictability. Pets have routines, but they also always surprise in some way each day, doing something different. And innocence. A pet is a product of its environment and upbringing, it's not at fault and it only does what it thinks it should or would like. Robots would be programmed, and so everything has an intention set by a human. |
| P53 | a real pet is a real companion, a robot is just emotionless and do not care about you |
| P54 | unpredictability. |
| P55 | Real pet exhibits emotions, shows affection, gives companionship, though at least a robot pet doesn't need house training ! |
| P56 | At present, a lack of adaptability to new situations. |
| P57 | robotic pets do not necessarily showcase a will of its own like a cat would. Robotic pets are more loyal in that they are usually designed to show signs of enjoying your interaction or touch. Some of them (paro) are also designed to squeal for attention when you are ignoring them. These behaviours might align to some extent with dogs but most definitely not with most cats, which often will ignore you or show their boundaries (which dogs do as well to some extent).   Furthermore interaction with real pets is more varied. You would feed them, go on walks or hikes, or do other activities with them (depending on the animal). Robotic pets are usually more limited in what you can do with them.   Finally, the touch-based experiences you have with robotic pets are very different. you can feel the fur and some movements and in the case of qoobo a heartbeat. but real animals display a range of biophysiological signals such as breathing, have a strong heartbeat, body temperature, etc. Which all contribute to an immersive experience of touching the animal. |
| P58 | their behaviour |
| P59 | Robot pet can get one because it is not the same as real pet and it is very good to take a practice before buying the real animals. |
| P60 | The real pet has emotion and each pet has distinct behaviour, but the robot all have the same pattern of emotion and behaviour. |
| P61 | owner’s responsibility, real pet could be dead |
| P62 | I would says 'lively' |
| P63 | Free will |
| P64 | emotion, care, love |
| P65 | I think real pet can sense emotions of human but robot can't |
| P67 | A real pet the touch and warmth feels like a heart-to-heart connection while a robot one might feel materialistic when touched or interacted with |
| P68 | idk |
| P69 | Real pet is a living being and robot pet is not a living-being |
| P70 | I sill feel interactive with real pet more than robot. Cause robot is artificial, they don't have heart and soul |
| P71 | Life? |
| P72 | Real pet have real emotions but robot pet need to be implement or it just a mimic of living beings |
| P73 | The nature of the maintenance of real and robot pets is completely different |
| P74 | liveliness |
| P75 | movement, personality |
| P76 | Emotion Feedback |
| P77 | A real pet is both proactive and reactive. It responds to someone or external stimuli, while also being able to do things on their own as well. |
| P78 | real pet have feeling and real bond to it, while robot pet still uncanny. |
| P79 | Unpredictability of a real pet. |
| P80 | Real pet are more unpredictable |
| P81 | Movement |
| P82 | Robot pet is more predicable than a real pet but real pet is more interactive. |
| P83 | their looks, movements, eye contacts |
| P84 | The robot pet is programmed / trained to do something. It is way too predictable and inorganic. |
| P85 | I think mostly its their movements, and emotions I feel that the real one seem lively they really do their own things without our interactions but robotics really is depended on us to make those movements. |
| P86 | their motion, their responsiveness are definitely not as fast as a real pet |
| P87 | It's simply not real. I'd be aware all the time that I was fooling myself by ascribing feelings and consciousness to it even if it was a 100% convincing simulation of a real animal. |
| P88 | I think that a robot pet would seem more artificial. |
| P89 | Never owned a real pet but it may be more unpredictable than a robot. |
| P90 | I think it's the inherent knowledge that the robot IS a robot and is specifically programmed in a certain way, to respond to certain things. Pets have the element of unpredictability that humans do that I think allows for a stronger bond. |
| P91 | Free will |
| P92 | The maintenance and upkeep a real pet requires is the main key difference. |
| P93 | One is alive and one is not... |
| P94 | The squishiness and cuteness aggression we get from animals. |
| P95 | a robot is not(at this stage in time) a real creature, it does not really act on it's on accord and does not react to it's environment in full detail, it is just going through pre-set responses  pets are not conscious or able to understanding the self but they can react to the self(physical harm, change in physiological states such as hunger or tiredness) and it can react to it's environment in relation to it's own needs(look for food, friends, owner etc, etc) |
| P96 | Robots are often very static and mechanical, they need to imitate real movement very well. I don’t know if i can verbalise what is so off putting about a poorly designed robo-pet |
| P97 | boredom, growth, unpredictability, health, affection, trust |
| P98 | Their unpredictability in how they act, you don’t know how they are going to show you affection but with a robot you know how they are going to act and someone has programmed it to do that. |
| P99 | Robots cannot fully replicate the deep emotional bonds between humans and real pets, |
| P100 | That emotional connection, free will and commitment that is required with a pet like a dog particularly but also to a lesser extent with others (e.g. cat, hamster etc). They depend on you for survival and happiness and you can try to train them but they have free will and can be annoying or difficult but that is rewarding in a way. |
| P101 | the responsibility differences, i feel like some people get dogs to learn how to look after something before having children (where not comparable, are similar in some distant ways) and if a robot was in the animals place there is no responsibility therefore no practice. also with animals you need to respect their boundaries (not wanting pet or something) where with a robot you can do what you want with it |
| P102 | The ability to create an emotional relationship with a real pet is not something I believe can be achieved with a robot. |
| P103 | Robot pets will always have an underlying program and code where as real pets are organic and have emotions, desires, survival instincts and the ability to pick up on emotional fluctuations. |
| P104 | I think I would view Cozmo, or an equally endearing robot, the same way I would a fictional character I enjoyed interacting with in a video game. He's endearing, but I don't think I'd ever rush him to the emergency vet in the middle of the night or spend hundreds of pounds on surgery (things I've done for Gizmo). I wouldn't feel guilty leaving him alone or ignoring him for a while. Living pets are a responsibility, a life you choose to tend and nourish. I've kept snails and fish, but my favourite pets have all been mammals, whose trust and affection I earned over time. I liked making them feel happy and safe, and I felt valued and loved in turn when they responded with behaviours like grooming me, asking for strokes and inviting me to play with them. |
| P105 | One is organic and one is pre programmed |
| P106 | An off switch |
| P107 | Interaction ,play time , bath time , food time and sounds |
| P108 | real pet is deliberate and gains a relationship, robot is programmed or made to do so. |
| P109 | life, warmth, instinct, they are water proof, you can climb a hill with them. |
| P110 | A pet had more free will and can have its own personality |
| P111 | they're typically dependant on online services run by corporations who may discontinue the product without warning |
| P112 | I do not know. |
| P113 | They are programs operations mimicking life, I feel more attachment to windows 10 than I do these robots |
| P114 | robots are artificial, programmed to perform an operation. I guess in a way, living things are as well, but I don't correlate living animals with robots... they are different entities. |
| P115 | Responsiveness to my emotions and to all our interactions |
| P116 | Genuine emotional connection |
| P117 | I guess the fact that you can turn it on and off and kind of design it to your specific needs and preferences. |
| P118 | A robot has programmed responses. A pet is a living thing that you have to build a real connection with |
| P119 | I feel less responsible for the robot pets. I feel like I can interact with them only whenever I want to whereas a real pet is an alive companion which I need to take care of no matter what my needs or conditions are. |
| P120 | Being a pet owner for a long time, an aspect I think that robot pets can't easily capture at this time is, seeing a pet learn or think about the world/environment they're in. Watching my own pets say watch out the window on a sunny day and seeing their eyes react and to me, see them think is what I think is a key difference from a real to robot pet. |
| P121 | A real pet has a mind of it's own, that's what makes having it's affection feel special. Robots cannot pick and chose who they feel connections with and treats everyone the same unless programmed otherwise. |
| P122 | Pets have their own will, while robots do not |
| P123 | Robot pets mostly come off as toys rather than pets, pets do their own things, where as these robot pets whole existence is you and once your done with it you put it in a drawer or something rather than the forced continued contact with another life that pets give you. |
| P124 | real pet can be unpredictable, robot may have some set of rules it has to follow being part of its full growth, if it loses a limb we will still love it, will not order a new part one from amazon Doesn't get the all the experience, the frustration of pet getting sick, and how to handle it. |
| P125 | I think a real pet has its own personality and I think for individuals that already have pets might find it difficult to adapt to a robot. |
| P126 | Robots don't have consciousness |
| P127 | Robots can recognise emotions like pets but they can’t empathise. Robots have a similar facial recognition to emotions to sociopaths, they can be trained to recognise but can’t feel it like a pet would |
| P128 | the ability to create mutual bond |
| P129 | You can turn off a robot pet and not get the jail. |
| P130 | A robot is not a pet. It might be a toy, but not a pet. |
| P131 | robot pets are made to simulate emotions within the human they interact with. pets have their own set of behaviours and dynamics humans (should) adapt to. |
| P132 | Lack of autonomy, not choosing to spend time with me, artificial rather than organic |
| P133 | I think a pet robot has a inert creepiness to it they are two separate things in my opinion and humans can’t emulate the connection with real pets |
| P134 | Taking it outside for walks. Animals don’t respond in human spoken language which gives you a challenge of communicating. Individual personality. The responsibility of caring for a living being. The hard parts (cleaning, feeding, vet visits, death) are all important to the relationship and you don’t get that same experience with a robot (except possibly charging it and it breaking but if it does break you can replace it unlike with a pet.) |
| P135 | Real pet grow up and changes but robot doesn't. |
| P136 | Agency, emotion, sentience |
| P137 | real pets have spontaneity and have make decision and don’t just do everything you want them you need to earn their trust and friendship |
| P138 | Real pets are their own "person" |
| P139 | Lack of will, a robot won't care if you start petting it. |
| P140 | A real pet is actually alive and breathing, a robot obviously isn't, but can appear to be. A robot is easier cause you don't need to feed it and can go on holiday and it'll be okay on it's own. |
| P141 | affection, building a relationship |
| P142 | touch, emotion.. |
| P143 | A real pet will be more interactive and lively and warm. The robot will feel like programmed and cold. |
| P144 | Real pets are alive. |
| P145 | Genuine connection between two conscious beings is only possible with a real pet and robots lack the ability to act spontaneously like a real animal can making them dull and predictable |
| P146 | It's the realism. Every robot I've ever seen is either not realistic at all, or somewhere in that grey area where you can tell something is amiss and it comes across as vacant and creepy. The interactions appear very programmed and limited. |
| P147 | The need for caring. A responsive relationship. |
| P148 | Programmed vs unprogrammed. Organic vs artificial. |
| P149 | a real pet is alive, so they interactions are unlimited, meanwhile a robot pet can only interact depending in what it is programmed for |
| P150 | Living breathing |
| P151 | Spontaneous and genuine emotional response. |
| P152 | A real pet is non-artificial, it has gone through the life cycle of growing instead of upgrades and have their own personalities not influenced or decided by humans. The very nature and nurtured life of pets seems completely different to the that of programmed and manmade robots. |
| P153 | A real pet is alive, and thus can die. No matter how cleverly programmed, a robot is not an organism. It may perform actions than humans understand as signalling interest, affection, need, but they are only performances with nothing underneath. There are no personal or societal consequences for maltreating or forgetting about a 'robot pet', any more than forgetting about or throwing away a microwave. |
| P154 | Aliveness will always be an issue. I struggle to see myself thinking the robot is actually alive |
| P155 | Robot pets are toys that require no emotional investment or commitment from the owner, while meeting selfish gratifications. Real pets are living creatures who require care, attention and love and who can choose how, when and if to show affection. |
| P156 | One is alive, the other isn't. |
| P157 | Real pet is instinctive, true emotion and feeling |
| P158 | Maybe the lack of a face, I don't know about that. |
| P159 | A real pet has higher stakes for physical and emotional caring, and the knowledge that you will experience real heartbreak if you outlive it. So the emotional rewards that I feel have stronger meaning (although of course I don’t think about them consciously in this way, most of the time). The mysteries of their inner lives, and the unpredictable path of our lives together, also gives me more permission to project my imagined and anthropomorphic interrelationship with them - especially that of loving and feeling loved by them. Until I believe that a robot has its own consciousness and needs, a companionable robot relationship would be founded entirely on my willing suspension of disbelief. |
| P160 | feelings |
| P161 | Real pets can act outside of their parameters and make their own needs known, their physical warmth, reactions (and purring) are too variable to be mechanical and they can exhibit behaviours that aren't tied to nearby humans. |
| P162 | Their freedom, autonomy & ability to hate me if they want to. I enjoy having a pet, mine likes me and I like him. But if when I adopted him from the shelter, he hated me, I would keep him & care for him regardless - that's a responsibility I feel deeply.  I feel like most robots are designed to love unconditionally, and that concept freaks me out a bit. If I am mean to my pet, it does not have to like me, if I am mean to a robot, it will be programmed to still care for me. Even if it is programmed to develop ill feelings towards me, it can be switched off, or factory reset. This is not possible with a pet (without killing the animal, which is inhumane) |
| P163 | Emotion, personality, genuine responses to human action, the requirement for care |
| P164 | A real pet has a brain and can make it's own decisions 24/7. A robot may be interactive and make it's own decisions, but it can be switched off and put away. A real pet is warm and alive, it is different form inanimate object. |

**Can you think of any future technological advancement that could make robots more suitable as household pets?**

| **PID** | **Future Advancements** |
| --- | --- |
| P1 | talk to someone who likes dogs - they’ll know! |
| P2 | Making robots softer and lighter, more reactive, and more agile. |
| P3 | show clear emotion, something that makes me want to pet it |
| P4 | Sure, if they were a pet for my pet. |
| P5 | sorry I cant |
| P6 | If they get more context awareness and agency, could move around freely and interact with humans in a unique way (which maybe the human could influence), give them personality. |
| P7 | for a truly pet-like experience, i think more reactive tendencies/qualities to the user's own actions are needed. something mirroring an animal as opposed to something humanoid would be better too |
| P8 | For a robot to imitate a cat, perhaps a random need for chaotic behaviour every so often  Not sure that'll pass the Ai safety tests though |
| P9 | More fluid movements to make them seem more lifelike, maybe. Right now everything still looks very mechanical. |
| P10 | Simply better range of movement, motion, and emotive feedback through improved motors, smaller tech, and similar would help a lot. |
| P11 | Absolutely none. You form an emotional bond with your pet. I can't see that happening with a piece of machinery. I wouldn't criticise someone if they got comfort from a robot pet if they were unable physically or mentally unable to care for a pet and it would give some form of company but it's a false company. These toys are only at a basic stage and just seem like something people would quickly tire of. The only one I'd consider is the vacuum which serves a purpose and isn't technically a toy. |
| P12 | Ah first they have to fulfil the owner's preference for a pet's looking. And it will be beneficial if they can take care and respond to their owners more. |
| P13 | Owner recognition plus independent locomotion to allow the pet to initiate contact. |
| P14 | Not really, I find the whole thing alienating. The problem of loneliness is one caused by our relations with other people and animals, not something that has a tech fix. |
| P15 | I think the main thing is just improving the sensing capabilities and movement capabilities of robots, and the complexity of modelling a 'personality' for one? You would want to be able to have a Robo-Pet 9000 that feels like an individual, and is different from your friend's Robo-Pet 9000, in the way that existing pets have their own personalities and behaviours |
| P16 | Unsure. I think long-lasting designs that could maintain themselves for people who are unable to have regular pets for health reasons, such as the elderly. |
| P17 | Addressing the warmth and softness of a companion robot would be good |
| P18 | I think if tech advances in a way that can make the robots more interactive, and those interactions more lifelike and organic, then it would keep the experiences with the pet robot feeling 'fresh', and like it has a personality. That for me would make it more similar to a real pet, and feel more like a proper alternative, as opposed to now where it's maybe more like a soft toy. |
| P19 | As the imagination of the programmers, and the information retention and processing capabilities of the robot increase I'm sure that there are some people who would benefit and enjoy a plastic pal. |
| P20 | As pets per se, no. A pet is a living thing, so unless robots were to be living organic beings, they could not compare with a living pet. If they were useful and could successfully and economically carry out household or other tedious work, then there might be a place for them, but then they would be tools: not pets |
| P21 | If they could misbehave and need care in an impromptu manner that takes control away from the owner! |
| P22 | It’ll happen. Deckard would come home from a hard days work retiring replicants to a synthetic slavering Labrador. AI programming and learning housed in a core body is on the cusp of reality which is a good/ bad thing. |
| P23 | I think if they moved in a less mechanical way and looked more similar to real pets (like a pet version of a 'reborn doll') then they would be more desirable as pets for people who cannot have real animals. |
| P24 | No! |
| P25 | More intelligent emotional responses and more natural, organic movements |
| P26 | Potentially but fear it would just fall down an AI hellhole or increase the risk of security breaches because people won't think their "pet" could prove to be a hazard with internet connection, facial recognition, and visual data about its environment. Keep it simple, keep it secure. |
| P27 | The robot dogs that I’ve seen universities experiment with look so lifelike in their movements despite aesthetically looking mechanical, and have potential uses as alternatives to guide dogs. I think advancements in AI could help with making robot pets satisfy more of the emotional benefits of having a pet to accompany useful physical features. |
| P28 | something LLMs, etc. for more fluid thinking, maybe? Hard to think of anything concrete. Maybe quieter/better motors might reduce some of the mechanical stiltedness of the robots shown? |
| P29 | User friendly tools to allow me to design/customise some combination of the sensing, intelligence, and physical form of a pet project. I think it would be easier to invest in something that was my own creation to some extent |
| P30 | No |
| P31 | No. I think robots should be a completely distinct category, although they can also be cute things that are kept in the house |
| P32 | What a difficult question, in the future l can envisage Robots being made to look like animals and for owners to treat them like that. No vets bills which are extortionate. Robots would I imagine probably require yearly checks like an MOT. |
| P33 | Model the behaviour of real pets, more realistic movement and appearance. It would also have to be somewhat independent, anything that breaks the illusion I think would reduce how meaningful I perceive the relationship to be. I can't help but place a high value on sentience, so the more you can fake that the better. |
| P34 | definitely not emotion since emotion would cause it to not be a robot anymore |
| P35 | Maybe some people, like the elderly could benefit if the robot was a cute pet-like companion and a helper too. I don't really want robot pets. Ivanka the roomba would be more like a helper. |
| P36 | Consciousness |
| P37 | Make them more realistic looking? |
| P38 | Once a robot has LLMs with emotional regulators attached that are keyed by facial/voice recognition, they should more reliably emulate complex pet interactions, such as joy when seeing the owner, or protective behaviour when strangers are near owner. |
| P39 | Self-determination. If the robot was able to give off warmth, like some animals do, that may help as well. |
| P40 | robots will continue to be developed, to have a wider range of actions and responses, to become less predictable in how they act and react. All of these things will make them appear more lifelike |
| P41 | Yes. But all of them have problems and disadvantages. Some of them are complete and utter security and privacy nightmares, others would at least on some level make robot a little bit dangerous (which no sensible company would ever risk and no regulator ever allow, especially as toys/pets for children). |
| P42 | Its hard. We have seen so many robots that gained a lot of attention but they tend to lose the excitement after a period of some time. Maybe a super intelligent bot from fictional movies like Baymax or Wall-E. But i think it would occur only when we are surrounded by a lot of AI and robotics and the cute ones could become more as pets but then regular pets like cats and dogs would still be more favourable. They could be as cute as the Apple's recent lamp robot but I am sceptical if they could be interesting for the people over a long time. Maybe if they become more soft, more useful, could communicate in English which pets lack, something like a B-Bot from Ron's Gone Wrong could become suitable as household pets. |
| P43 | Recognize emotions/gestures through their cameras |
| P44 | Not sure. I'm not a pet person. |
| P45 | Mobility is a pretty big factor, I think. A dog can run up to me and nuzzle up against me, and otherwise go do its own thing. A robot right now would probably be pretty slow, so if I wasn't actively in need of affection, I would probably be tempted to just turn it off so that it wouldn't get in the way. But that feels like it detracts from the bond a little bit. Part of why ChatGPT feels like a close friend in some ways is that it's always around and at the ready, and all you have to do it ask it something. |
| P46 | Be able to eat as real pet and waterproof as if we take them to go outside while it is snowing or raining. I hope it would still be ok and works as a real pet. |
| P47 | None for me. Other people may have other things in mind when wanting a pet. For me, a pet is not a 'service-giver' which can be copied by a robot. It's a living being that is enjoyable to be with, and I accept that I care for it. |
| P48 | I'm sure they will become more lifelike and less robotic in movement in the future. |
| P49 | No |
| P50 | Increasing how responsive they are to the world, like cosmo, would help indicate that they can see and process the world, and are not just playing out a set of predefined instructions. A Qoobo-like pet with Cosmo's style reaction would be a fun addition as a household pet, and would make it seem more alive |
| P51 | responsiveness |
| P52 | A wider range of behaviours and inputs that they can sense from humans than just touch and general noise, so more nuanced phrases or intonation like animals can identify. And more autonomy, so they aren't just responding to each input that the human provides. |
| P53 | maybe realism and movement |
| P54 | longevity |
| P55 | Perhaps they do need to have some imperfections! |
| P56 | More sophisticated software |
| P57 | I think they would be more beneficial if they could talk. Animals cannot talk and but robots could. This would set them apart from real pets instead of just trying to imitate them.   Furthermore if robotic pets could be more life-like (displaying temperature, heartbeats, etc) it would make it more realistic and immersive to engage with them. |
| P58 | if they could perfectly replicate the pet behaviour |
| P59 | I think it would be lots of robots to help us in the future as cleaning a house or even drive a car for us. |
| P60 | - |
| P61 | kid friendly, waterproof |
| P62 | Maybe? |
| P63 | AI |
| P64 | i would not replace my dog with a robot |
| P65 | not sure |
| P67 | Maybe adding soft and more fur that makes it more realistic and eyes that could be covered as real human eyes, more lively. |
| P68 | I don’t think they would be a pet in general, at best a nice household device/tool that's pleasing to live with. Never crossing the line of pets |
| P69 | Maybe make it more real and have a function that will not do such behaviours that the owner do not like (not at all because I think for some owners, they really hate when their pet do stuff that is quite unpleasant.) |
| P70 | More functionality and look more real |
| P71 | People might be more accepting of robots if they are cute and is helpful to some degree |
| P72 | The Machine learning would be good if the robot pet can learn by themselves like human and animals that can adapt to different environments |
| P73 | The only thing would be an AI that adapts to the owner and his/her actions that cannot be tampered with |
| P74 | Ai |
| P75 | texture, warmth, fluid movement |
| P76 | Fine tune motor, life like skin and fur, Eyes mechanics |
| P77 | Make them more reactive to what someone does, but also be able to do things on its own too. Make it feel more alive. |
| P78 | being more fluid in it's movement i think would help a lot but fur and textures or essentially skin being more living creature like would also help |
| P79 | Making robot feel more like an actual pet. This lower the cost of pet caring (Pet food, Yearly check up) |
| P80 | More realistic skin texture |
| P81 | Microchip |
| P82 | I think that it movement is a huge key it need to be responsive and flexible like real pet but still do need to keep it robot like plus it need more interaction that you can play with it. |
| P83 | not at the moment |
| P84 | No, I see those pet robots as a gimmick. |
| P85 | I think that the seal is nice, I feel that if i acted a-bit more faster then I would seem more responsive. |
| P86 | add more function to the robot, make it more responsive. |
| P87 | A wider range of expressions and interactivities. The robotic JCB and the seal were interesting but a bit limited in what they do. But what's the point of playing 'fetch' with a robot when you *know* it isn't enjoying it the way my dog does. |
| P88 | Company for individuals who are unable to care for household pets due to age, disability etc |
| P89 | Cyborgs not robots. |
| P90 | I think it is very much dependent on the 'purpose' of the pet - e.g. if someone were to get a fish or a dog. Is the pet something more visual and less interactive, or something that requires lots of interaction. |
| P91 | I think humans will always want a pet that is alive like they are |
| P92 | . |
| P93 | Maybe a more authentic style of 'personality'. Something that can evolve and change, and is unique to 'your pet'. I remember when Furbies came out, they marketed them as having individual personalities and capable of learning languages. While that wasn't true, it was very appealing. |
| P94 | We gotta get to Bladerunner levels before I think it is a fully viable options and fulfills all our needs. |
| P95 | the ability to physically react to the self and the ability to react to the environment in relation to the self(physically not philosophically) |
| P96 | I think we have everything we need to create a pleasant robo-pet, it will just take time and effort to perfect |
| P97 | I don't think they should be, in the large scheme of things. I would be concerned that they'd be treated as badly as real animals, and that there would be psychological consequences on how we view animals. I think it's cute and quirky, but I don't think robots should be made to replace companionship in a world where 1 billion animals are killed every week you know? it feels wrong. I think animal rights should come first, then we can think about robot ones, at least in general. HOWEVER, the last pet robot with the tail and heartbeat could be useful in things like retirement homes, where animals are known to be beneficial but also can be stressed out. apart from situations like this though I don't think they need be any more lifelike. |
| P98 | No, I think they can improve life in helping us do chores or jobs but not giving affection and emotions |
| P99 | more sophisticated AI for nuanced emotional responses and advanced tactile sensors to mimic real touch. |
| P100 | Not really, I see them as tools or devices that can help or make life easier. Maybe they could be teachers for young children or companions for elderly people in specific circumstances but not pets. For example I remember bringing my dog to visit my elderly grandmother in a care home and all the residents and staff flocked to him wanting to pet him/ feed him etc. I cannot imagine a robot ever having this instant impact on an entire room of people (not many humans would have this impact come to think of it!). |
| P101 | I’m not too sure, with the rate we are going there will probably be artificial pets soon enough but i don’t think i would be open to getting one |
| P102 | I cannot |
| P103 | Robots will never be able to replace real pets but by improving AI technology and emotion recognition, they can get closer to replicating the good parts of having a pet while removing the bad parts. |
| P104 | I'm not sure. A large obstacle, I think, will be suspending people's disbelief. You need to convince them that the robot is acting under its own power, driven by genuine feelings - of curiosity, fear, affection, frustration etc. I think the most potential probably lies in looking at how animators, puppeteers etc. imbue objects with apparent life (the muppets, animated robots like Wall-E etc.). Don't try to perfectly mimic existing animals, this is uncanny; it may be easier for the imagination to accept "this is a machine come to life" than "this is a real puppy". |
| P105 | Not sure |
| P106 | Longer battery life and autonomy. |
| P107 | No |
| P108 | no |
| P109 | Tools for lightening the household chores yes, pets other than toys for kids NO |
| P110 | I have no idea but I’d love to know. I would love to have a pet but have bad allergies so I’d love to be able to find this out if that’s possible |
| P111 | Not be reliant on mobile/computer apps to function (except for maybe updates) as these can be discontinued rendering them useless, this may negatively effect an individual who has established an emotional dependence on them |
| P112 | I do not know. |
| P113 | If robots develop far enough to be considered alive keeping them as pets would be cruel, they would be their own intelligent sentient species at that point |
| P114 | nope! |
| P115 | Ability to program them to individual specifications; make them look more life-like and responsive in different ways |
| P116 | Perhaps if true consciousness could be created. Otherwise, it's more akin to something like decoration. |
| P117 | Generative AI - but most likely already it is already used |
| P118 | General functionality. A roomba could be a pet, in a sense, if it could communicate. So long as they do not try to pretend they aren't robots. A real pet will always be better as you can earn and build connections that aren’t artificial |
| P119 | More personalisable elements |
| P120 | Better AI I would guess for more personally interactions with Robot Pets. I think also for the people making robot pets is, to let go of recreating things we see in this world of recreate the human like designs of ourselves. Qoobo and Cozmo show that you don't really need to recreate, just create something new. |
| P121 | AI learning and replicating the thought process of a pet? Generating it's own personality and being able to learn and make it's on memories in real time. |
| P122 | Shape simulation may make robots more like pets |
| P123 | As bad as it is, Implementing artificial intelligence into these bots probably would do a lot, additionally having them be able to be more mobile (easily going up and down stairs), with longer battery life or charging themselves without issue or person intervention would make a large difference. |
| P124 | no, pet has to be real and living.  the robot are just a mechanical toy |
| P125 | No |
| P126 | AI to mimic consciousness |
| P127 | Not sure |
| P128 | unsure |
| P129 | Mass extinction of real animals is why the robots pets in blade runner exists. Space travel could work. Have a wee buddy while cry or sleeping |
| P130 | No. Absolutely not. Machines are tools, not companions. |
| P131 | safety and data protection |
| P132 | No |
| P133 | Ig commercially appeal to people’s loneliness or laziness |
| P134 | If they become more societally acceptable then more people would probably buy them. To make them look more like animals. A full range of movement. Focussing on animals that people commonly own as pets. Smoother movement. |
| P135 | No |
| P136 | No, they’re purely for mechanistic/informatic tasks |
| P137 | ai developments to create more decisions |
| P138 | Not sure |
| P139 | Improved spatial reasoning and visual perception could make robots appear smarter and more perceptive of their environment |
| P140 | Being able to be really independent, and help with household chores and tasks. Also being more interactive with conversations, so you can actually talk to them and they give intelligent and emotional answers. |
| P141 | the robot being more proactive and approaching, rather than always responding |
| P142 | no |
| P143 | Having more interactive features and lively movements. |
| P144 | That it is not detectable that its a robot. |
| P145 | if a robot which could complete a variety of household chores was created i could see it being worthwhile, although, that would be more of a servant than a pet |
| P146 | AI, obviously. Future enhancements in this area will allow for more realistic interactions, in a wider variety of circumstances. It could also be leveraged to make movements more realistic. The mechanics need to be improved too to reduce these robotic like movements. |
| P147 | Not for me I am afraid! |
| P148 | I think we'll play around with AI, but it's still artificial. Robots should not replace real life. |
| P149 | AI will help them to have more interaction and behaviour options |
| P150 | None |
| P151 | No |
| P152 | Not really, it doesn't seem feasible. |
| P153 | No. Having worked on robot research, I fundamentally think there is no future for household robotics, but that they have immense value in other applications, like in industry or operating in hostile environments. |
| P154 | Better environment sensing |
| P155 | No. |
| P156 | Incorporating emotional design features and the ability to perform some tasks that some users such as myself, may have access to now, but don't use / engage with due to a lack of a physical presence. |
| P157 | Trust in the technology. |
| P158 | I would like to test their interactions with human beings through natural language processing. |
| P159 | Yes, the in the appropriate balance of tensions between evolving AI capabilities, and our understanding of what is responsible and healthy in the capabilities and constraints of those systems. More intelligent, autonomous, and physically capable robots could be more like the original working pets of the past - including today’s service animals, with greater powers to guide, detect hidden risks to our wellbeing, and take action. Any kind of collaborative relationship and interest-dependence is helpful. (After all, dogs that want to be walked make us want to walk with them out in our neighbourhood, to the benefit of our physical, mental, and collective social health.) |
| P160 | not really |
| P161 | Better mobility. Though honestly I suspect holographic representations of real animals are where this idea will go sooner or later. |
| P162 | I don't know that I could ever come to love a robot as a pet.   Generational comfort may be a thing - my currently 5 year old nephew will grow up around more sophisticated robots than I did, and the generation after him even more so. Maybe this exposure from a young age will have allow people to engage with robots without the gut feeling & prejudice that I have.  Alternatively, if they could replicate a pet & it's mannerisms so closely that I am unable to determine it is a robot, I'd probably feel the same way about it as I do real animals |
| P163 | AI and cheaper more cost-effective construction |
| P164 | I think it is normal to be lonely, and having a pet like robot could help in some aspects, but I don't believe it will ever be the same as having a real pet. It is also a bit sad and weird to have a robot dog rather than a real dog, I think picking up the phone and playing a game with a pet dog could be more 'normal' than having a robot pet. |
